# SLIM: A Short‐Linked, Highly Redox‐Stable Trityl Label for High‐Sensitivity In‐Cell EPR Distance Measurements

**DOI:** 10.1002/anie.202004452

**Published:** 2020-04-30

**Authors:** Nico Fleck, Caspar A. Heubach, Tobias Hett, Florian R. Haege, Pawel P. Bawol, Helmut Baltruschat, Olav Schiemann

**Affiliations:** ^1^ Institute of Physical and Theoretical Chemistry University of Bonn Wegelerstr. 12 53115 Bonn Germany; ^2^ Institute of Physical and Theoretical Chemistry University of Bonn Römerstr. 164 53117 Bonn Germany

**Keywords:** EPR spectroscopy, in-cell measurements, radicals, spin labeling, trityl radical

## Abstract

The understanding of biomolecular function is coupled to knowledge about the structure and dynamics of these biomolecules, preferably acquired under native conditions. In this regard, pulsed dipolar EPR spectroscopy (PDS) in conjunction with site‐directed spin labeling (SDSL) is an important method in the toolbox of biophysical chemistry. However, the currently available spin labels have diverse deficiencies for in‐cell applications, for example, low radical stability or long bioconjugation linkers. In this work, a synthesis strategy is introduced for the derivatization of trityl radicals with a maleimide‐functionalized methylene group. The resulting trityl spin label, called SLIM, yields narrow distance distributions, enables highly sensitive distance measurements down to concentrations of 90 nm, and shows high stability against reduction. Using this label, the guanine‐nucleotide dissociation inhibitor (GDI) domain of *Yersinia* outer protein O (YopO) is shown to change its conformation within eukaryotic cells.

## Graphical abstract

**SLIM fit**: A trityl label with a short linker and high reduction stability, called SLIM, was synthesized. It enables highly sensitive pulsed dipolar EPR measurements down to low nanomolar concentrations and yields narrow distance distributions. Its exemplary use in in‐cell measurements showed that YopO preferably adopts one of the two conformations found in vitro.

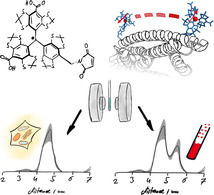

## Introduction

**Figure 1:**
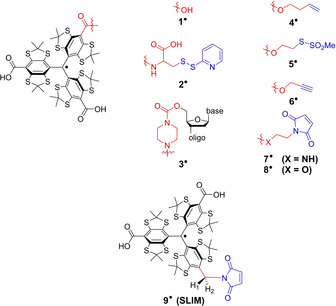
Finland trityl **1^.^**, various trityl labels (**2^.^**–**8^.^**) reported in the literature, and the new trityl label **9^.^** (SLIM). For the sake of clarity, the radical basis is depicted in black, the linker in red, and the bioconjugation site in blue.

Carbon‐centered trityl radicals have emerged as important molecules for in‐vivo imaging,^1^ oximetry,^2^, ^3^ pH‐sensing.^3^ and as polarizing agents in dynamic nuclear polarization (DNP)^4^, ^5^ experiments. Additionally, the so‐called Finland trityl **1^.^** 6 (Figure 1) has been used for synthesizing trityl‐based spin labels **2^.^**–**8^.^** 7, 8, 9, 10, 11 out of which **2^.^** 7 and **3^.^** 8 paved the way for biomolecular structure determination at physiological temperatures using pulsed dipolar electron‐paramagnetic‐resonance spectroscopy (PDS).^12^, ^13^ Furthermore, trityl labels **4^.^** and **7^.^** have been shown to be suitable for PDS measurements within cells.^9^, ^14^ Advantages of trityl labels are their long relaxation times *T*_M_ at room temperature,^15^ their single‐line EPR spectra yielding large signal‐to‐noise ratios (SNR),^16^ their spin state of *S*=1/2 , which makes data analysis simple,^17^ and their increased reduction stability compared to *gem*‐dimethylnitroxides allowing for in‐cell measurements.^9^, ^14^ Although such in‐cell measurements are possible, the currently used trityls are still reduced within cells.^18^ In contrast, Gd^III^‐based spin labels are inert to reduction within cells, but, depending on the particular type of the complex, the Gd^III^ ion may be exchanged for metal ions present in the cell.^19^ The relaxation times *T*_M_ of Gd^III^ can be shorter or longer than those of trityls, depending on the utilized ligand,^14^, ^20^ and the electron‐spin state of *S*=7/2 imposes challenges on data analysis.^21^, ^22^ Thus, in order to keep the trityl core but to make it more suitable for in‐cell measurements, its redox properties have to be tuned, possibly by exchanging the electron‐withdrawing carboxy substituents with electron‐donating groups. Furthermore, the currently used synthesis strategy for introducing the bioconjugation group via esterification (**4^.^**–**6^.^**, **8^.^**)^9^, ^11^ or amidation (**2^.^**, **3^.^**, **7^.^**)^7^, ^8^, ^10^ of the carboxylic groups (Figure 1) leads to long, flexible linkers that make the PDS‐derived distance distributions broad and, in some cases, multimodal.^11^ This, in turn, renders the interpretation of such distance distributions error‐prone. Last but not least, the label should not be cleaved from the biomolecule under in‐cell conditions, rendering the ester connectivity of the bioconjugation group to the trityl core^23^ and the disulfide bridge forming a methanethiosulfonate group^24^ unsuitable. With respect to the latter, the thioether‐forming maleimide group has been confirmed to be advantageous.^25^

Therefore, the work presented herein introduces a synthesis by which the maleimide group is coupled to the trityl core via just one methylene group leading to the label **9^.^**, called SLIM (short‐linked maleimide), which provides narrow distance distributions, increased stability against reduction, high labeling efficiencies, and large signal‐to‐noise ratios in PDS measurements.

## Results and Discussion

### Synthesis and Characterization

**Scheme 1:**
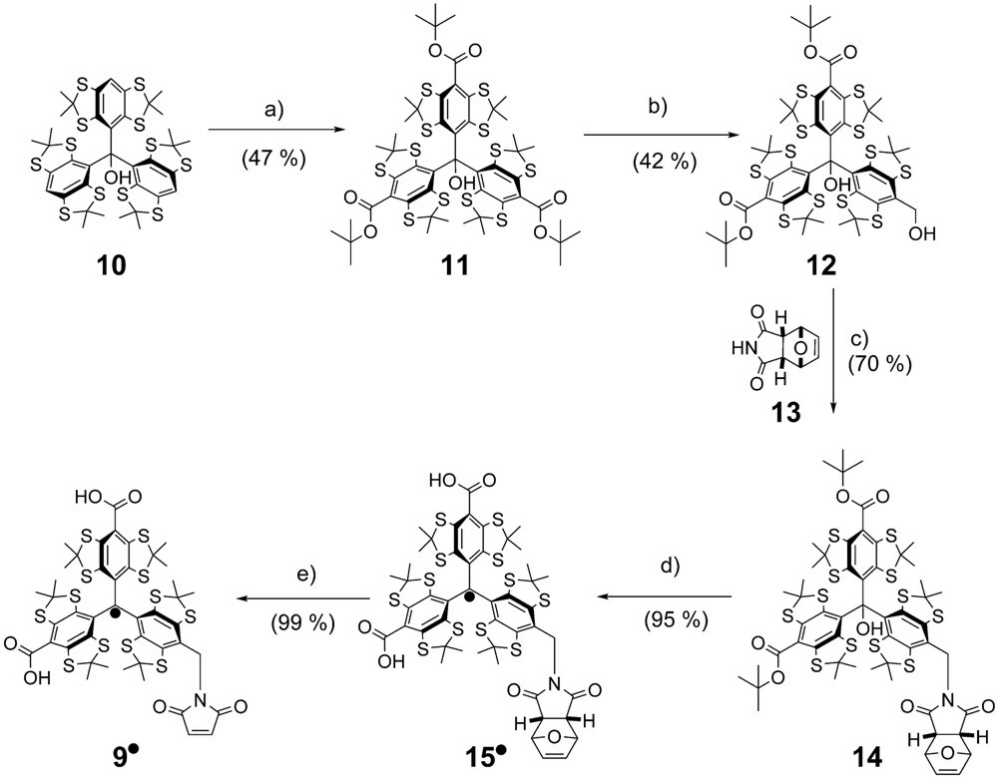
Synthesis of SLIM **9^.^**. a) 1) *n*‐BuLi, TMEDA, Et_2_O, rt, 0.5 h; 2) *N*‐*tert*‐butoxycarbonylpyridinium *tert*‐butanolate, Et_2_O, 24 h. b) LiAlH_4_, THF, rt, 1 h. c) Ph_3_P, diethyl azodicarboxylate, THF, 0 °C, 0.5 h. d) 1) CF_3_COOH, CH_2_Cl_2_, rt, 2 h; 2) SnCl_2_, THF, 0.3 h. e) CH_3_CN, 60 °C, 24 h.

The synthesis of SLIM **9^.^** is shown in Scheme 1 and starts from trityl alcohol **10**, which can be obtained from 1,2,4,5‐tetrachlorobenzene in three steps.^9^ Subsequent deprotonation and treatment with activated Boc‐anhydride afforded the threefold ester **11** by adapting a recent protocol of Hintz et al.^26^ Statistical reduction of one ester moiety with LiAlH_4_ broke the *C*_3_ symmetry and lead to **12** in a yield of 42 % (58 % based on recovered starting material **11**).

**Figure 2:**
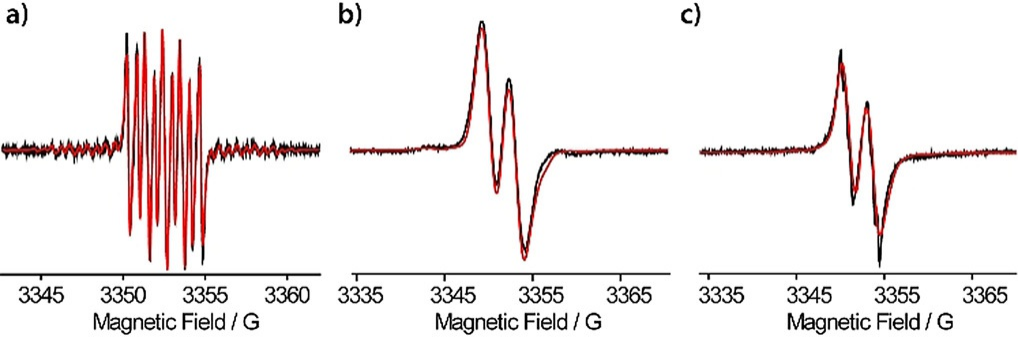
Cw X‐band EPR spectra of **9^.^** in a PBS buffer a) at 298 K, b) at 100 K, and c) of **9^.^** bound to the single‐cysteine mutant YopO N624C in a PBS buffer at 298 K.

In the next step, the required C−N bond on the way to **9^.^** was formed in a Mitsunobu reaction^27^ between **12** and **13** leading to **14** in a yield of 70 %. The excellent Michael‐acceptor properties of maleimides required the protection of their C=C bond in form of the Diels–Alder adduct **13** in order to prevent side reactions with Ph_3_P,^28^ which is needed as a reagent in the Mitsunobu transformation. The *endo*‐isomer of **13** 29 was chosen over the *exo*‐isomer, because it provides sufficient retro‐Diels–Alder reactivity already at 60 °C (see Supporting Information, Section 2.2.4) instead of 150 °C, which is necessary for the cleavage of the *exo*‐adduct of **13**.^30^, ^31^ The deprotection at 60 °C is compatible with the thermal stability of the radical center,^32^ which is crucial for the final deprotection to **9^.^**. However, first, the *t*‐butyl esters in **14** are cleaved by triflouroacetic acid concomitant to the abstraction of the hydroxyl group. This leads to tritylium ion **15^+^**, which is then reduced in situ with tin(II) chloride to **15^.^**. Finally, **9^.^** was obtained by simply heating **15^.^** to 60 °C overnight leading to a quantitative deprotection of the maleimide. Relative to starting compound **10**, the overall yield of the five‐step synthesis was 13 %. The identity and purity of **9^.^** was confirmed by high‐resolution mass spectrometry and HPLC (see Supporting Information, Sections 2.3.2–2.3.3). For further characterization, a continuous‐wave (cw) X‐band EPR spectrum of **9^.^** in a PBS buffer (PBS=phosphate‐buffered saline) was recorded at room temperature (Figure 2 a). The spectrum displays nine major lines due to hyperfine coupling of the electron spin to the imido nitrogen atom (*A*_N_=1.71 MHz) and the two benzylic hydrogen atoms (*A*_H1_=6.00 MHz, *A*_H2_=2.96 MHz). The fact that the hyperfine‐coupling constants of H1 and H2 (Figure 1) differ from each other is also seen in DFT calculations and can be attributed to the helical chirality of the trityl scaffold (see Supporting Information, Section 8.1). Freezing the sample to 100 K resulted in the EPR spectrum shown in Figure 2 b with a splitting between both lines of 7.44 MHz, which is, in large parts, governed by the hyperfine coupling to H1. Thus, and in contrast to the Finland trityl derivatives **2^.^**–**8^.^**, SLIM **9^.^** does not give rise to a single line in the frozen state. However, the spectral width of ≈10 G is still, on the one hand, narrow enough to permit full excitation with conventional rectangular pulses and, on the other hand, broad enough to also enable PELDOR experiments.

### Redox Stability

With respect to in‐cell measurements, the stability of a spin label against reduction is important. As shown in the literature,^33^, ^34^, ^35^ the *para*‐substituents of trityl radicals hold a strong influence on the electrode potentials. Generally, the carbanion T^−^ is stabilized by electron withdrawing groups, such as esters or amides, resulting in an increase of the reduction potential. This implies that all spin labels obtained by esterification or amidation of **1^.^** are more prone to reduction than **1^.^** itself. In contrast, the imidomethylene substituent in **9^.^** rather acts as an electron‐donating group, destabilizing the corresponding carbanion and restraining the reduction compared to **1^.^**. Indeed, this behavior is seen in the cyclovoltammograms (Figure S28, Supporting Information). The reduction potential of **9^.^** is lowered by 46 mV compared to **1^.^**, furnishing it with an increased stability towards reduction. Due to the higher reactivity of the corresponding carbanion **9** ^−^ towards H^+^, its reduction is less reversible than for **1^.^**, as seen when using slower scan rates (see Supporting Information, Section 4.1). In contrast to the reduction, the oxidation of **9^.^** is slightly promoted by 26 mV compared to **1^.^**. Nonetheless, no oxidative degradation was observed under ambient conditions.

**Figure 3:**
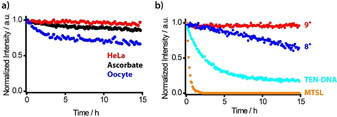
a) Plot of the EPR intensities (double integral) vs. time for 200 μm **9^.^** (red), **8^.^** (blue), tetraethyl nitroxide **S5** (cyan), and MTSL (orange) in a PBS buffer containing 4.75 mm sodium ascorbate, each corresponding to a 24‐fold molar excess of ascorbate. b) Plot of the EPR intensities (double integral) against time for **9^.^** conjugated to YopO N624C (50 μm) in HeLa‐lysate (red), 4.75 mm ascorbate (black), and *Xenopus laevis* oocyte lysate (blue). The initial intensities were comparable, and the dead time was below 6 min in each case. Label **S5** was conjugated to DNA to provide sufficient water solubility.

In order to probe the in‐cell persistence of **9^.^**, its cw‐EPR‐signal intensity was monitored over time under several commonly used and in‐cell‐related conditions.^18^, ^36^, ^37^ In a 4.75 mm ascorbate solution (Figure 3 a), **9^.^** does not decay at all, whereas trityl label **8^.^** decays to 62 % within 15 h, the *gem*‐diethyl label **S5** bound to DNA (see Supporting Information, Section 3.1) is reduced to 18 % in the same time and the *gem*‐dimethyl label MTSL is completely reduced within 1.5 h.

In another step, **9^.^** was conjugated to *Yersinia* outer protein O (YopO) mutant N624C and the labeled protein was added to a 4.75 mm solution of ascorbate, HeLa cell lysate, and oocyte lysate. As can be seen in Figure 3 b, also under these conditions, **9^.^** is reduced only marginally in the case of ascorbate and HeLa lysate. Even within *Xenopus laevis* oocyte lysate, the most reducing cell lysate tested herein, only a decay to 71 % is observed after 15 h. Label **9^.^** is thus considerably more stable than the *gem*‐dimethyl nitroxides and at least on par with the best *gem*‐diethyl nitroxides according to literature reports.^36^, ^37^

### Spin Labeling

Successful spin labeling requires high site‐selectivity and high labeling efficiency. In order to probe for the first aspect, the cysteine‐free mutant of YopO^38^, ^39^ was incubated with **9^.^** under typical labeling conditions.^11^ MALDI‐MS showed the mass for the unlabeled protein only, indicating that no other amino acid is covalently labeled by **9^.^** (see Supporting Information, Section 3.3.5). Non‐covalent labeling and the presence of inseparable aggregates of **9^.^** were tested for by using UV/Vis spectroscopy. The UV/Vis spectrum after labeling shows a weak absorption band at 464 nm (see Supporting Information, Section 3.3.4) indicating that 6.9±0.6 % of non‐bound **9^.^** are present in the sample relative to the protein. This behavior of trityls is known^10^, ^11^, ^40^ and, in this case, actually quite effectively diminished by the labeling protocol.

The efficiency of the bioconjugation was subsequently examined using the single‐cysteine YopO mutant N624C. ESI‐MS (see Supporting Information, Section 3.3.5) confirmed that only one label is bound. The labeling efficiency was estimated to be quantitative based on ESI‐MS and 94±9 % based on UV/Vis and EPR spin‐counting experiments. Interestingly, **9^.^** covalently bound to YopO yields a room‐temperature cw X‐band EPR spectrum similar to that of **9^.^** free in the frozen state, which can be simulated by only slightly adjusting the EPR parameters of **9^.^** at 100 K (Figure 2 and Supporting Information, Section 5). Thus, the slow rotation of **9^.^** bound to a protein brings the label into the rigid limit and enables the distinction of bound label from unbound label.

### Distance Measurements

**Figure 4:**
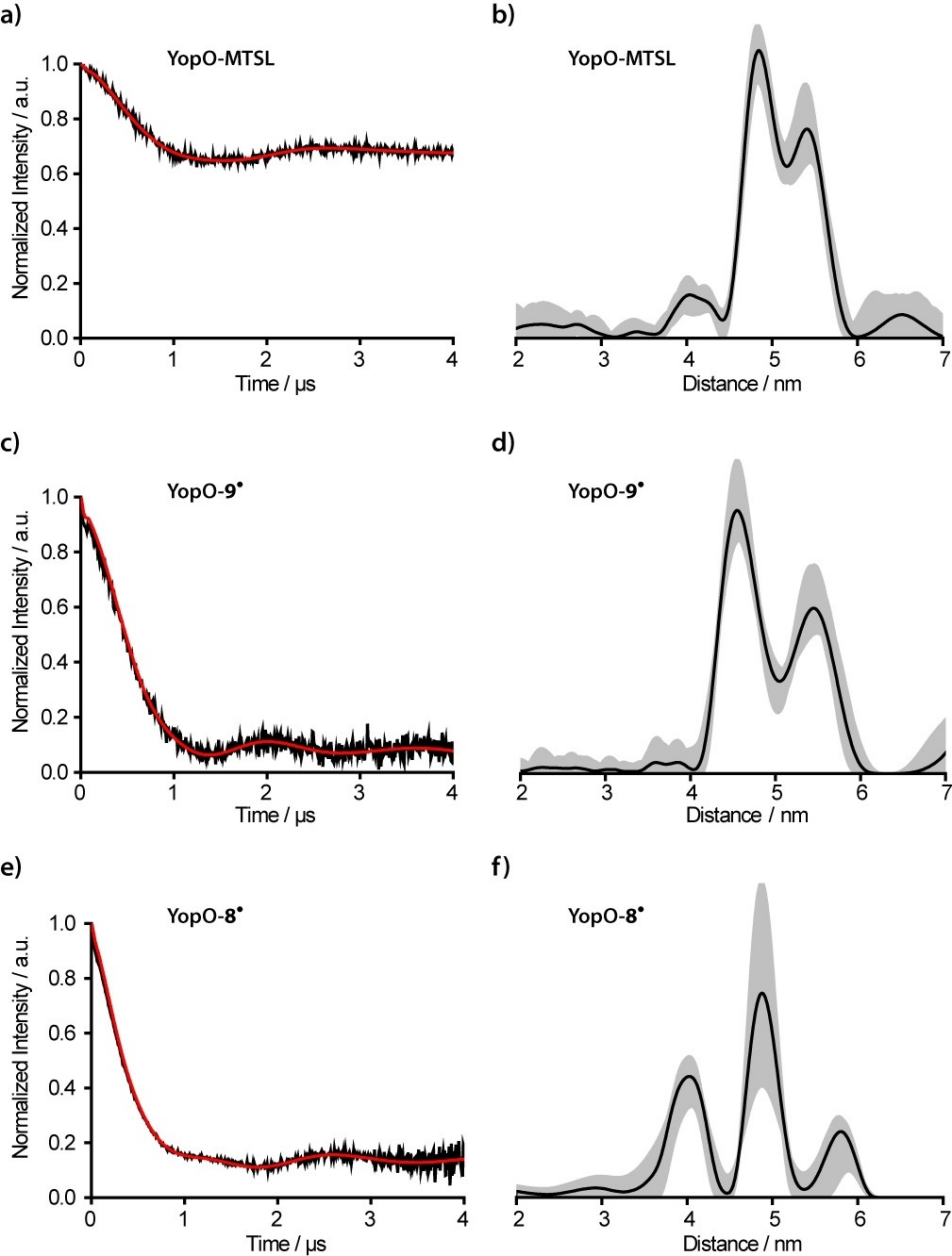
PDS experiments on mutant Y588C/N624C‐YopO labeled with a), b) MTSL (PELDOR), c), d) **9^.^** (DQC), and e), f) **8^.^** (DQC). Background‐corrected time traces (black) are given along with their fits (red) in (a,c,e), and the resulting distance distributions are provided in (b,d,f) in black with the corresponding DeerAnalysis validation shown as grey shaded areas.

In a next step, the effect of the reduced linker length on PDS derived distance distributions was assessed on the double‐cysteine mutant YopO Y588C/N624C (see Supporting Information, Section 3.3.1) by labeling it with **9^.^**, **8^.^**, and MTSL. The resulting doubly labeled constructs YopO‐**9^.^**, YopO‐**8^.^**, and YopO‐MTSL were characterized (see Supporting Information, Section 3.3.4) and subjected to double‐quantum coherence (DQC)^41^, ^42^ and pulsed electron–electron double‐resonance (PELDOR)^43^, ^44^, ^45^ experiments whose background‐corrected time traces are shown in Figure 4 for original time traces, see the Supporting Information, Section 7.5). The PELDOR time trace of YopO‐MTSL (Figure 4 a) exhibits the typical modulation depth of 32 % for Q‐band PELDOR and a SNR of 248 h^−1/2^. The corresponding distance distribution shows a bimodal distribution, which was seen before for other MTSL‐YopO mutants involving α‐helix 14 in the guanine‐nucleotide dissociation inhibitor (GDI) domain.^11^ For YopO‐**9^.^**, the narrow spectral width of the trityl signal called for a DQC experiment, which almost tripled the modulation depth to 87 % and the SNR to 674 h^−1/2^ (Figure 4 c). This high SNR prompted us to reduce the YopO‐**9^.^** concentration to 90 nm, which still gave an SNR of 2 h^−1/2^ at a time window length of 2.5 μs (see Supporting Information, Section 7.7). Performing PELDOR measurements on YopO‐**9^.^** provided a SNR of only 155 h^−1/2^ (see Supporting Information, Section 7.5). This shows that the combination DQC/**9^.^** outperforms the PELDOR/MTSL combination.^11^ Notably, the bimodality seen for YopO‐MTSL is also resolved for YopO labeled with **9^.^**, and both the widths and weights of the two modes are very similar in both cases (see Supporting Information, Section 7.5).

Since the bimodality is observed for two different spin labels, MTSL and **9^.^**, and two PDS techniques, PELDOR and DQC, it can be related to two different conformers of the α‐helix, as previously discussed.^11^, ^39^ The peak at 4.5 nm is assigned to the straight form of α‐helix 14 (PDB‐ID: 2h7o) and the peak at 5.3 nm to its bent form (PDB‐ID: 4ci6).^11^, ^39^ In the crystal structures, the bent form is only found when actin is bound, whereas here, in frozen solution, both conformations of α‐helix 14 seem to be present even in the absence of the actin ligand. Interestingly, the addition of human platelet actin did not change the obtained distance distribution, strongly indicating that the conformation of α‐helix 14 is independent of the actin‐binding process (see Supporting Information, Section 7.6).

In contrast, the DQC experiment on YopO‐**8^.^** (SNR of 503 h^−1/2^) provides a broad trimodal distance distribution (Figure 4 e,f), which is attributed to the longer linker and thus a broader range of label conformers for **8^.^**, especially with shorter distances (see Supporting Information, Section 8.2.2). The differences in the conformer space of **8^.^** and **9^.^** can be quantified in silico^46^ via the accessible volume both labels sample.^47^, ^48^ This yielded 15 200 Å^3^ and 6940 Å^3^ for **8^.^** and **9^.^**, respectively, and thus, a reduction of the conformer space by 54 % upon going from **8^.^** to **9^.^**. This example thus nicely highlights the importance of a short linker group as provided by the new SLIM label.

### In‐Cell Measurements

To test the feasibility of **9^.^** for in‐cell structure elucidation, DQC measurements on the aforementioned Y588C/N624C‐YopO mutant were performed within eukaryotic *Xenopus laevis* oocytes. The rationale behind the choice of this type of cells as model system is twofold: first, oocytes exhibit the highest reducing activity of all cell types under study^36^ (Figure 3 c) and do, thus, serve as a true in‐cell benchmark test for **9^.^**. Second, although YopO is a prokaryotic protein, its full enzymatic function is only initiated upon translocation into eukaryotic immune cells through the *Yersinia* type‐3 secretion system, a needle‐like structure that penetrates the outer membrane of the innate immune cells.^49^, ^50^ Here, the oocytes serve as the eukaryotic species and their size enables mimicking this translocation process of YopO‐**9^.^** using a microinjection system (see Supporting Information, Section 6). In this way, samples with a bulk spin concentration of 11 μm were obtained and subjected to Q‐band DQC experiments.

**Figure 5:**
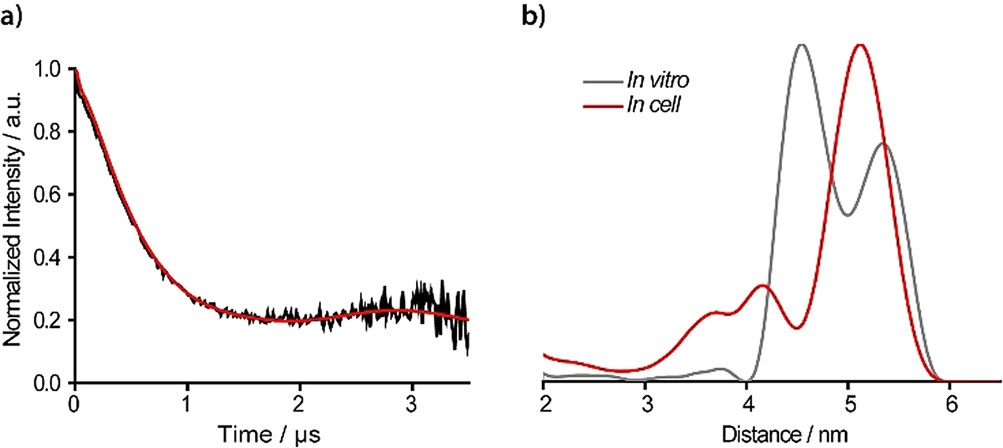
a) Background‐corrected in‐cell DQC time trace and the corresponding fit of YopO‐**9^.^** after an incubation period of 2 h. b) Distance distribution of the in cell experiment (red) overlaid with the distance distribution obtained in vitro (3.5 μs trace length, grey).

Due to the presence of Mn^II^ in oocytes and spin‐crowding effects, the phase‐memory time *T*_M_ is shortened compared to in‐vitro measurements (see Supporting Information, Section 7.8). However, an incubation of the injected oocytes over 2 h led to a more uniform distribution of the labeled protein within the oocytes enabling a dipolar‐evolution‐time window of 3.5 μs for the in‐cell DQC experiment. The obtained time trace (Figure 5 a) exhibits a SNR of 23 h^−1/2^ (2 h^−1/2^ μm ^−1^), which is considerably higher than previously reported for in‐cell measurements with nitroxide‐36, 51, 52 and trityl‐labeled^9^ biomolecules. Even in comparison to W‐band PELDOR/trityl and PELDOR/Gd^III^ measurements, the Q‐band DQC/SLIM combination is at least on par.^14^, ^53^, ^54^, ^55^, ^56^ Remarkably, the distance distribution from the in‐cell measurement differs from the in‐vitro‐derived ones (Figures 4 d and S38,S39). This can already be seen when comparing the time traces; the in‐cell time trace has a considerably longer oscillation period (3 μs) than the in‐vitro counterparts (2 μs). Accordingly, the long‐distance peak at 5.1 nm prevails within oocytes and is now the dominating peak, whereas the peak at 4.5 nm is strongly diminished. This data thus indicates a preferred selection of the bent form of α‐helix 14 of the GDI domain of YopO in the eukaryotic cytosol. The straight conformation of α‐helix 14 leads to shorter inter‐spin distances, which are well pronounced in the in‐vitro experiments but are strongly diminished in the in‐cell measurement (compare the Supporting Information, Section 7.8). This effect may be related to molecular crowding^57^, ^58^ and/or binding of regulatory proteins such as Rac1^59^ in the eukaryotic cytosol. More in‐depth studies on this will follow.

## Conclusion

In this work, the trityl spin label SLIM was introduced and probed for its suitability in PDS experiments. Its synthesis involved a Mitsunobu‐type transformation using a protected maleimide, which can be deprotected in a mild retro‐Diels–Alder reaction. Bioconjugation of SLIM to cysteines proceeds in high yields and site‐selectively. Its narrow spectral width enables high‐sensitivity distance measurements down to low nanomolar protein concentrations, and the short linker leads to narrow and, thus, more reliable distance distributions. Additionally, SLIM features a high stability towards reduction, making in‐cell PDS measurements at high SNRs feasible. In profit of this, it could be shown that the injection of YopO into a eukaryotic cell leads to a change in the conformational ensemble of the GDI domain. Thus, SLIM is a very promising label improving the capability to obtain structural information from biomolecules within their natural cellular environment.

## Conflict of interest

The authors declare no conflict of interest.

## Supporting information

As a service to our authors and readers, this journal provides supporting information supplied by the authors. Such materials are peer reviewed and may be re‐organized for online delivery, but are not copy‐edited or typeset. Technical support issues arising from supporting information (other than missing files) should be addressed to the authors.

Supplementary

## References

[1] B. B. Williams , H. J. Halpern , in Biomedical EPR—Part A: Free Radicals, Metals, Medicine and Physiology (Eds.: S. R. Eaton, G. R. Eaton, L. J. Berliner), Springer US, Boston, 2005, pp. 283–319.

[2] B. Epel , C. R. Haney , D. Hleihel , C. Wardrip , Med. Phys. 2010, 37, 2553–2559. 20632567 10.1118/1.3425787 PMC2881926

[3] A. A. Bobko , I. Dhimitruka , J. L. Zweier , V. V. Khramtsov , J. Am. Chem. Soc. 2007, 129, 7240–7241. 17511458 10.1021/ja071515u

[4] G. Mathies , M. A. Caporini , V. K. Michaelis , Y. Liu , K. N. Hu , D. Mance , J. L. Zweier , M. Rosay , M. Baldus , R. G. Griffin , Angew. Chem. Int. Ed. 2015, 54, 11770–11774; 10.1002/anie.201504292 PMC5407364 26268156

[5] J. H. Ardenkjær-Larsen , B. Fridlund , A. Gram , G. Hansson , L. Hansson , M. H. Lerche , R. Servin , M. Thaning , K. Golman , Proc. Natl. Acad. Sci. USA 2003, 100, 10158–10163. 12930897 10.1073/pnas.1733835100 PMC193532

[6] S. Andersson , A. Rydbeck , R. S. Mahno , US Patent 5728370, 1999.

[7] Z. Yang , Y. Liu , P. Borbat , J. L. Zweier , J. H. Freed , W. L. Hubbell , J. Am. Chem. Soc. 2012, 134, 9950–9952. 22676043 10.1021/ja303791p PMC3409244

[8] G. Y. Shevelev , O. A. Krumkacheva , A. A. Lomzov , A. A. Kuzhelev , O. Y. Rogozhnikova , D. V. Trukhin , T. I. Troitskaya , V. M. Tormyshev , M. V. Fedin , D. V. Pyshnyi , et al., J. Am. Chem. Soc. 2014, 136, 9874–9877. 24963806 10.1021/ja505122n

[9] J. J. Jassoy , A. Berndhäuser , F. Duthie , S. P. Kühn , G. Hagelueken , O. Schiemann , Angew. Chem. Int. Ed. 2017, 56, 177–181; 10.1002/anie.201609085 27918126

[10] A. Giannoulis , Y. Yang , Y.-J. Gong , X. Tan , A. Feintuch , R. Carmieli , T. Bahrenberg , Y. Liu , X.-C. Su , D. Goldfarb , Phys. Chem. Chem. Phys. 2019, 21, 10217–10227. 30860214 10.1039/c8cp07249c

[11] J. J. Jassoy , C. A. Heubach , T. Hett , F. Bernhard , F. R. Haege , G. Hagelueken , O. Schiemann , Molecules 2019, 24, 2735. 10.3390/molecules24152735 PMC6696014 31357628

[12] G. W. Reginsson , O. Schiemann , Biochem. Soc. Trans. 2011, 39, 128–139. 21265760 10.1042/BST0390128

[13] G. Jeschke , Annu. Rev. Phys. Chem. 2012, 63, 419–446. 22404592 10.1146/annurev-physchem-032511-143716

[14] Y. Yang , B. Pan , F. Yang , Y. Liu , X. Su , D. Goldfarb , J. Phys. Chem. Lett. 2020, 11, 1141–1147. 31951412 10.1021/acs.jpclett.9b03208 PMC7307952

[15] A. A. Kuzhelev , D. V. Trukhin , O. A. Krumkacheva , R. K. Strizhakov , O. Y. Rogozhnikova , T. I. Troitskaya , M. V. Fedin , V. M. Tormyshev , E. G. Bagryanskaya , J. Phys. Chem. B 2015, 119, 13630–13640. 26001103 10.1021/acs.jpcb.5b03027 PMC4830422

[16] G. W. Reginsson , N. C. Kunjir , S. T. Sigurdsson , O. Schiemann , Chem. Eur. J. 2012, 18, 13580–13584. 22996284 10.1002/chem.201203014

[17] N. C. Kunjir , G. W. Reginsson , O. Schiemann , S. T. Sigurdsson , Phys. Chem. Chem. Phys. 2013, 15, 19673–19685. 24135783 10.1039/c3cp52789a

[18] A. P. Jagtap , I. Krstic , N. C. Kunjir , R. Hänsel , T. F. Prisner , S. T. Sigurdsson , Free Radical Res. 2015, 49, 78–85. 25348344 10.3109/10715762.2014.979409

[19] M. Qi , A. Groß , G. Jeschke , A. Godt , M. Drescher , J. Am. Chem. Soc. 2014, 136, 15366–15378. 25325832 10.1021/ja508274d

[20] M. Azarkh , A. Bieber , M. Qi , J. W. A. Fischer , M. Yulikov , A. Godt , M. Drescher , J. Phys. Chem. Lett. 2019, 10, 1477–1481. 30864799 10.1021/acs.jpclett.9b00340 PMC6625747

[21] D. Goldfarb , Phys. Chem. Chem. Phys. 2014, 16, 9685–9699. 24429839 10.1039/c3cp53822b

[22] D. Abdullin , O. Schiemann , ChemPlusChem 2020, 10.10002/cplu.201900705. 31950648

[23] Z. Yang , M. D. Bridges , C. J. López , O. Y. Rogozhnikova , D. V. Trukhin , E. K. Brooks , V. Tormyshev , H. J. Halpern , W. L. Hubbell , J. Magn. Reson. 2016, 269, 50–54. 27214582 10.1016/j.jmr.2016.05.006 PMC4958593

[24] R. Igarashi , T. Sakai , H. Hara , T. Tenno , T. Tanaka , H. Tochio , M. Shirakawa , J. Am. Chem. Soc. 2010, 132, 8228–8229. 20513154 10.1021/ja906104e

[25] R. Roser , M. J. Schmidt , M. Drescher , D. Summerer , Org. Biomol. Chem. 2016, 14, 5468–5476. 27181459 10.1039/c6ob00473c

[26] H. Hintz , A. Vanas , D. Klose , G. Jeschke , A. Godt , J. Org. Chem. 2019, 84, 3304–3320. 30785294 10.1021/acs.joc.8b03234

[27] K. C. K. Swamy , N. N. B. Kumar , E. Balaraman , K. V. P. P. Kumar , Chem. Rev. 2009, 109, 2551–2651. 19382806 10.1021/cr800278z

[28] M. A. Walker , J. Org. Chem. 1995, 60, 5352–5355.

[29] E. H. Discekici , A. H. St. Amant , S. N. Nguyen , I. H. Lee , C. J. Hawker , J. Read De Alaniz , J. Am. Chem. Soc. 2018, 140, 5009–5013. 29613783 10.1021/jacs.8b01544 PMC6205238

[30] O. K. Farha , R. L. Julius , M. F. Hawthorne , Tetrahedron Lett. 2006, 47, 2619–2622.

[31] Z. Lu , R. Weber , R. J. Twieg , Tetrahedron Lett. 2006, 47, 7213–7217. 18584068 10.1016/j.tetlet.2006.07.142 PMC2440721

[32] N. Fleck , T. Hett , J. Brode , A. Meyer , S. Richert , O. Schiemann , J. Org. Chem. 2019, 84, 3293–3303. 30813730 10.1021/acs.joc.8b03229

[33] X. Tan , L. Chen , Y. Song , A. Rockenbauer , F. A. Villamena , J. L. Zweier , Y. Liu , Chem. Res. Toxicol. 2017, 30, 1664–1672. 28759716 10.1021/acs.chemrestox.7b00086

[34] C. Decroos , V. Balland , J. L. Boucher , G. Bertho , Y. Xu-Li , D. Mansuy , Chem. Res. Toxicol. 2013, 26, 1561–1569. 24010758 10.1021/tx400250a

[35] B. Driesschaert , A. A. Bobko , T. D. Eubank , A. Samouilov , V. V. Khramtsov , J. L. Zweier , Bioorg. Med. Chem. Lett. 2016, 26, 1742–1744. 26923698 10.1016/j.bmcl.2016.02.048 PMC4807691

[36] G. Karthikeyan , A. Bonucci , G. Casano , G. Gerbaud , S. Abel , V. Thomé , L. Kodjabachian , A. Magalon , B. Guigliarelli , V. Belle , et al., Angew. Chem. Int. Ed. 2018, 57, 1366–1370; 10.1002/anie.201710184 29227566

[37] T. S. Braun , P. Widder , U. Osswald , L. Groß , L. Williams , M. Schmidt , I. Helmle , D. Summerer , M. Drescher , ChemBioChem 2020, 21, 958–962. 31657498 10.1002/cbic.201900537 PMC7187341

[38] E. E. Galyov , S. Håkansson , Å. Forsberg , H. Wolf-Watz , Nature 1993, 361, 730–732. 8441468 10.1038/361730a0

[39] M. F. Peter , A. T. Tuukkanen , C. A. Heubach , A. Selsam , F. G. Duthie , D. I. Svergun , O. Schiemann , G. Hagelueken , Structure 2019, 27, 1416–1426. 31303480 10.1016/j.str.2019.06.007

[40] I. Marin-Montesinos , J. C. Paniagua , A. Peman , M. Vilaseca , F. Luis , S. Van Doorslaer , M. Pons , Phys. Chem. Chem. Phys. 2016, 18, 3151–3158. 26742686 10.1039/c5cp05767a

[41] S. Saxena , J. H. Freed , Chem. Phys. Lett. 1996, 251, 102–110.

[42] S. Saxena , J. H. Freed , J. Chem. Phys. 1997, 107, 1317–1340.

[43] A. D. Milov , K. M. Salikhov , M. D. Schirov , Fiz. Tverd. Tela 1981, 23, 975–982.

[44] A. Milov , A. Ponomarev , Y. Tsvetkov , Chem. Phys. Lett. 1984, 110, 67–72.

[45] M. Pannier , S. Veit , A. Godt , G. Jeschke , H. W. Spiess , J. Magn. Reson. 2000, 142, 331–340. 10648151 10.1006/jmre.1999.1944

[46] G. Hagelueken , R. Ward , J. N. Naismith , O. Schiemann , Appl. Magn. Reson. 2012, 42, 377–391. 22448103 10.1007/s00723-012-0314-0 PMC3296949

[47] K. Sale , L. Song , Y. Liu , E. Perozo , P. Fajer , J. Am. Chem. Soc. 2005, 127, 9334–9335. 15984837 10.1021/ja051652w

[48] G. Hagelueken , D. Abdullin , O. Schiemann , Methods Enzymol. 2015, 563, 595–622. 26478500 10.1016/bs.mie.2015.06.006

[49] G. V. Plano , K. Schesser , Immunol. Res. 2013, 57, 237–245. 24198067 10.1007/s12026-013-8454-3

[50] W. L. Lee , J. M. Grimes , R. C. Robinson , Nat. Struct. Mol. Biol. 2015, 22, 248–255. 25664724 10.1038/nsmb.2964 PMC4745138

[51] I. Krstić , R. Hänsel , O. Romainczyk , J. W. Engels , V. Dötsch , T. F. Prisner , Angew. Chem. Int. Ed. 2011, 50, 5070–5074; 10.1002/anie.201100886 21506223

[52] P. Widder , J. Schuck , D. Summerer , M. Drescher , Phys. Chem. Chem. Phys. 2020, 22, 4875–4879. 32072999 10.1039/c9cp05584c

[53] A. Martorana , G. Bellapadrona , A. Feintuch , E. Di Gregorio , S. Aime , D. Goldfarb , J. Am. Chem. Soc. 2014, 136, 13458–13465. 25163412 10.1021/ja5079392

[54] Y. Yang , F. Yang , Y. Gong , J. Chen , D. Goldfarb , X. Su , Angew. Chem. Int. Ed. 2017, 56, 2914–2918; 10.1002/anie.201611051 28145030

[55] Y. Yang , F. Yang , Y. Gong , T. Bahrenberg , A. Feintuch , X. Su , D. Goldfarb , J. Phys. Chem. Lett. 2018, 9, 6119–6123. 30277780 10.1021/acs.jpclett.8b02663

[56] F. Wojciechowski , A. Groß , I. T. Holder , L. Knörr , M. Drescher , J. S. Hartig , Chem. Commun. 2015, 51, 13850–13853. 10.1039/c5cc04234h 26236790

[57] H.-X. Zhou , FEBS Lett. 2013, 587, 1053–1061. 23395796 10.1016/j.febslet.2013.01.064 PMC3729036

[58] Y. Wang , M. Sarkar , A. E. Smith , A. S. Krois , G. J. Pielak , J. Am. Chem. Soc. 2012, 134, 16614–16618. 22954326 10.1021/ja305300m

[59] G. Prehna , M. I. Ivanov , J. B. Bliska , C. E. Stebbins , Cell 2006, 126, 869–880. 16959567 10.1016/j.cell.2006.06.056

