# Supplementary material for: SLIM: A Short‐Linked, Highly Redox‐Stable Trityl Label for High‐Sensitivity In‐Cell EPR Distance Measurements
Source: Angew Chem Int Ed Engl. 2020 Apr 30;59(24):9767–72. doi: 10.1002/anie.202004452 (PMC7318235; doi:10.1002/anie.202004452)
Supplement: Supplementary file 1 — Supplementary [file ANIE-59-9767-s001.pdf]

Angewandte Chemie
Eine Zeitschrift der Gesellschaft Deutscher Chemiker

Supporting Information

SLIM: A Short-Linked, Highly Redox-Stable Trityl Label for High-Sensitivity In-Cell EPR Distance Measurements

*Nico Fleck, Caspar A. Heubach, Tobias Hett, Florian R. Haege, Pawel P. Bawol, Helmut Baltruschat, and Olav Schiemann**

anie_202004452_sm_miscellaneous_information.pdf

Author Contributions

N.F. Investigation: Lead; Writing—Original Draft: Lead
T.H. Formal analysis: Lead; Investigation: Supporting; Writing—Original Draft: Supporting
F.H. Investigation: Supporting
P.B. Investigation: Supporting
H.B. Writing—Review & Editing: Supporting.

Table of Contents

1. General Procedures and Instrumentation 3

2. Synthesis 4

2.1 General Procedures Synthesis 4

2.2 Synthetic Protocols 4

2.2.1 Synthesis of **S2** 4

2.2.2 Synthesis of **S3** 5

2.2.3 Synthesis of **S4** 5

2.2.4 Retro Diels-Alder reaction: Kinetics 5

2.2.5 Synthesis of literature known compound **11** 6

2.2.6 Synthesis of **12** 7

2.2.7 Synthesis of **14** 8

2.2.8 Synthesis of **15**$^{\bullet}$ 9

2.2.9 Synthesis of **9**$^{\bullet}$ 9

2.3 Analytical Data Synthesis 10

2.3.1 NMR Spectra 10

2.3.2 HRMS-Data 15

2.3.3 UHPLC Data 18

3. Spin Labeling 19

3.1 Tetraethylnitroxide-DNA Construct 19

3.2 SLIM Dilution Series and Calibration Curve 19

3.3 *Yersinia* outer protein O (YopO) 20

3.3.1 Construct Design 20

3.3.2 YopO Expression 21

3.3.3 YopO Purification 21

3.3.4 YopO Spin Labeling 22
3.3.5 YopO Mass Spectrometry 24
4. Redox Stability 26
4.1 Cyclic Voltammetry 26
4.2 Reduction Stability 26
4.2.1 Cell Lysates Preparation 26
4.2.2. Stability Measurements 27
5. Simulation of *cw*-EPR spectra 28
6. *In Cell* Sample Preparation 30
7. Pulsed EPR 31
7.1 EPR Sample Preparation 31
7.2 Relaxation Time Measurements 31
7.3 Double Quantum Coherence (DQC) Experiments 32
7.4 Pulsed Electron-Electron Double Resonance (PELDOR) Experiments 33
7.5 YopO PDS Results 34
7.6 PDS on YopO with human platelet actin 36
7.7 Evaluation of Concentration Limit for SLIM / DQC 36
7.8 *In Cell* Pulsed EPR Measurements 38
8. Theoretical Results 40
8.1 Density Functional Theory Computations 40
8.2 *In Silico* Spin Labeling 44
8.2.1 Conformer Cloud Volume 44
8.2.2 YopO **8•** Label Conformer Selection 45
9. Literature 46

1. General Procedures and Instrumentation

NMR spectra were recorded on Avance I 300, Avance I 400, Avance III HD 500, or Avance III HD 700 spectrometers (Bruker BioSpin, Rheinstetten, Germany). Chemical shifts are reported referenced to hydrogen residual peaks of the NMR solvent.[5]

MALDI(+)-spectra were recorded in a Bruker Daltonics UltraFlex TOF/TOF spectrometer (Bruker, Rheinstetten, Germany). For organic compounds, DCTB was used as the matrix, whereas DHAP was used for protein samples. For ESI(+), a Synapt G2-Si spectrometer (Waters, Milford, USA) was employed. APCI-spectra were obtained on an Orbitrap XL spectrometer (Thermo Fisher Scientific, Bremen, Germany). Protein samples were subjected to mass spectrometric analysis in 20 mM PO_i buffer (pH = 6.8, 50 mM NaCl).

For analytical chromatography, an UHPLC system (PLATINblue-series, Knauer GmbH, Berlin, Germany) equipped with a Eurospher II 100-2 C18P, 2 µm, 2 x 100 mm column (Knauer GmbH, Berlin, Germany) and a UV detector working at 265 nm was used.

All continuous wave (*cw*) EPR measurements were performed at X-band frequencies (~9.4 GHz) either on an EMXmicro or EMXnano spectrometer (Bruker BioSpin, Rheinstetten, Germany). Measurements at room temperature were done on the EMXmicro using an ER 4122SHQ resonator. Measurements at 100 K were conducted using a Bruker 4119HS resonator in conjunction with a ER 4141VT temperature control system, which operates with a continuous flow of nitrogen gas. For all samples, X-band EPR-tubes with an outer diameter of 5 mm obtained from Wilmad Labglass (Brand of SP Industries, Warminster, USA) were used. Aqueous samples were, however, filled into 10 µL capillaries (Disposable Capillaries, Hirschmann Laborgeräte, Eberstadt, Germany), which were then transferred into the X-band tubes.

cw-EPR spectra of the pure SLIM label and after bioconjugation were recorded in frozen solution using a modulation amplitude of 0.2 G, a microwave power of 7.4 µW (44 dB attenuation), a time constant of 20.48 ms and a conversion time of 20.59 ms. 2000 data points were recorded, corresponding to a resolution of 25 points per G. Quantitative EPR measurements were conducted employing the on-board spin counting routine of the EMXnano spectrometer. *CW*-EPR measurements at room temperature were performed at an attenuation of 25 dB (603.1 µW) and a modulation amplitude of 0.1 G.

Pulsed EPR measurements were conducted at Q-band frequencies (~33.7 GHz) on a Bruker (Bruker BioSpin, Rheinstetten, Germany) ELEXSYS E580 EPR spectrometer equipped with an ER5106QT-II resonator and a 150 W TWT-amplifier (Applied Systems Engineering, Fort Worth, TX, USA). All data was acquired using quadrature detection. The temperature was adjusted to the appropriate value (between 10 K and 50 K) using a CF935 helium gas-flow cryostat (Oxford Instruments, Abingdon, UK) in conjunction with an Oxford Instruments iTC 503 temperature controller.

2. Synthesis

2.1 General Procedures Synthesis

Chemicals were purchased from commercial suppliers and used without further purification. Diethyl ether was dried over 4 Å molecular sieves, and THF was distilled over sodium wire with benzophenone. Where indicated, solvents were degassed by applying three freeze-pump-thaw cycles. Thin layer chromatography was conducted using 250 µm F254 silica plates provided by Merck, and spots were visualized with UV-light at 254 nm. Spots of trityl alcohols can be stained selectively by irradiating the TLC-plate for 5 min with UV-light at 254 nm (5 W). For column chromatography, silica gel (60 Å pore size, 40 – 63 µm particle size) purchased from Merck was used. Solvents were generally removed under reduced pressure by a rotary evaporator, products were further dried in oil-pump vacuum at 10^{-3} mbar. The concentration of *n*-butyl lithium solutions was determined prior to use by titration following the protocol of *Winkle et al.*[1] Trityl alcohol **10** was obtained following our previously published protocol.[2] 3aRS,4SR,7RS,7aSR-4,7-epoxy-3,3a,7,7a-tetrahydro-2H-isoindoli-1,3-dione (**13**) was prepared according to the literature.[3] Compound **S1** was obtained following the procedure of *Hintz et al.*[4] using DMF instead of N-formylpiperidine.

2.2 Synthetic Protocols

2.2.1 Synthesis of **S2**

2,2,6,6-tetramethyl-8-(hydroxymethyl)benzo[1,2-d;4,5-d]bis(1,3-dithiol)

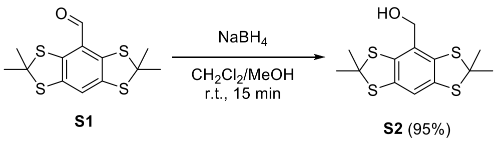

S1 (800 mg, 2.5 mmol) was dissolved in 15 mL dry dichloromethane and 20 mL dry methanol were added. Then, sodium borohydride (300 mg, 3.83 mmol, 1.50 eq.) was added, whereupon the yellow solution lost its color. After 15 minutes of stirring at room temperature, the solvents were removed under reduced pressure and water (30 mL) was added. The aqueous phase was extracted with ethyl acetate (3 x 20 mL) and the unified organic phases were dried over $MgSO_4$. After removal of the solvents, the crude product is obtained as an off-white solid, which was then purified by column chromatography on silica eluting with CyH/EtOAc (9:1) giving **S2** (R_f = 0.18) in a yield of 767 mg (95%).
^{1}H-NMR (400 MHz, $CDCl_3$, 298 K, δ in ppm): 7.01 (s, 1H), 4.56 (s, 2H), 1.89 (s, 12H).
^{13}C{^{1}H}-NMR (100 MHz, $CDCl_3$, 298 K, δ in ppm): 136.6, 136.0, 129.5, 116.5, 65.3, 65.2, 31.5.
HRMS (ESI+, *m/z,* $[M]^+$): calc. for $C_{13}H_{16}OS_4$, 316.0078; found 316.0077.

2.2.2 Synthesis of **S3**

2,2,6,6-tetramethyl-8-(1-methyl(3aRS,4SR,7RS,7aSR-4,7-epoxy-3,3a,7,7a-tetrahydro-2H-isoindoli-1,3-dione-2-yl)- benzo[1,2-d;4,5-d]bis(1,3-dithiol)

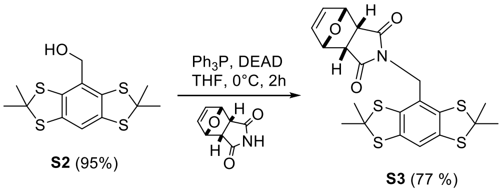

S2 (300 mg, 0.95 mmol, 1.25 eq.), **13** (125 mg, 0.76 mmol), and Ph_3P (200 mg, 0.76 mmol) were dissolved in 10 mL dry THF. Then, DEAD (132 mg, 120 µL, 0.76 mmol) was added and the reaction mixture was stirred for 1 h at 0°C. Subsequently, the reaction mixture was poured onto water (50 mL) and extracted with CH_2Cl_2 (2 x 30 mL). The unified organic phases were dried over $MgSO_4$ and solvents were removed under reduced pressure. The product (R_f = 0.3) was isolated by column chromatography on silica eluting with CyH/EtOAc (2:1) in a yield of 338 mg (77%).
^{1}H-NMR (500 MHz, $CDCl_3$, 298 K, δ in ppm): 6.99 (s, 1H), 6.44 (bs, 2H), 5.29 (m, 2H), 4.37 (s, 2H), 3.54 (m, 2H), 1.86 (s, 12H).
^{13}C{^{1}H}-NMR (125 MHz, $CDCl_3$, 298 K, δ in ppm): 173.9, 136.6, 136.6, 134.9, 124.1, 116.6, 79.4, 65.4, 46.1, 43.1, 31.2.
HRMS (APCI, *m/z,* $[M+H]^+$): calc. for $C_{21}H_{22}NO_3S_4$, 464.0477; found 464.0474.

2.2.3 Synthesis of **S4**

2,2,6,6-tetramethyl-8-(1-methyl(3,4-dehydropyrolidin-1,3-dione-2-yl)benzo[1,2-d;4,5-d]bis(1,3-dithiol)

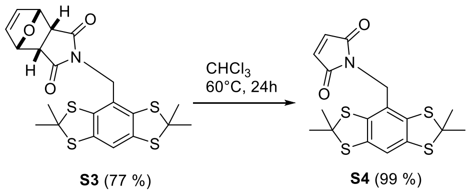

S3 (100 mg) was dissolved in 20 mL chloroform and stirred at 60 °C overnight and under an argon atmosphere. Afterwards, all volatiles were removed and **S4** was obtained in a quantitative yield of 85 mg as a colorless solid.
^{1}H-NMR (500 MHz, $CDCl_3$, 298 K, δ in ppm): 7.01 (s, 1H), 6.68 (s, 2H), 4.57 (s, 2H), 1.87 (s, 12H).
^{13}C{^{1}H}-NMR (125 MHz, $CDCl_3$, 298 K, δ in ppm): 169.73, 136.76, 136.16, 134.15, 124.94, 116.48, 65.62, 42.06, 31.25.
HRMS (ESI+, *m/z,* $[M+H]^+$): calc. for $C_{21}H_{22}NO_3S_4$, 316.0215; found 316.0213.

2.2.4 Retro Diels-Alder reaction: Kinetics

Since **15$^•$** is paramagnetic and therefore not suitable for NMR-measurements, model compound **S3** was used in order to examine the Retro-Diels-Alder reaction leading to **9$^•$**. For this, 100 mg of **S3** were dissolved in 5 mL $CDCl_3$ and heated to 60 °C under argon. Then, samples of 0.5 mL were drawn out of the reaction mixture with a syringe, cooled, and subjected to ^{1}H-NMR analysis immediately. Since **S3** and its Retro-Diels-Alder product **S4** can be differentiated by NMR, it was possible to extract the reaction kinetics, which follows a first order rate law as shown in Figure S1. After 1380 min (23 h), complete conversion to the free maleimide was observed.

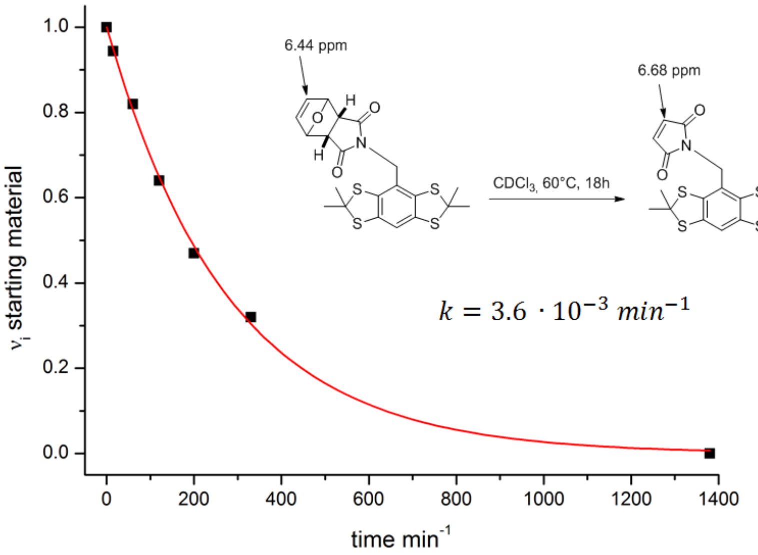

Figure S1: *Kinetics for Retro-Diels-Alder reaction based on ¹H-NMR. Experimentally determined molar ratio of starting material (black dots) with exponential fit (red line).*

Based on a first-order rate law, a rate constant of 0.0036 min^{-1} was found by least-square fitting. This value compared well to rate constants obtained for similar furane-maleimide adducts.[6] From this, it was concluded that the tetrathioaryl-substituent does not influence the Retro-Diels-Alder reactivity. Therefore, the same conditions were applied in the synthesis of **9•**.

2.2.5 Synthesis of literature known compound **11**

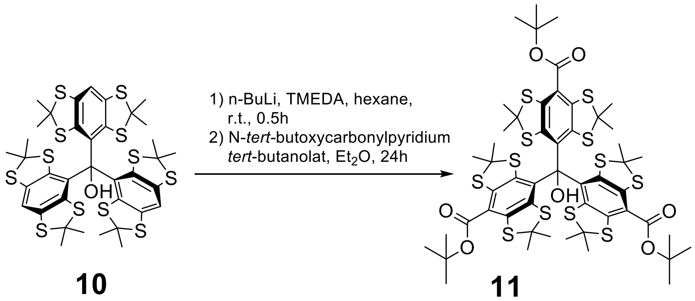

10 **11**

Tris[2,2,6,6-tetramethyl-8-(carboxy-tert-butyl)benzo[1,2-d;4,5-d]bis(1,3-dithiol)-4-yl]methanolTrityl alcohol **10** (3.00 g, 3.40 mmol) was dissolved in 100 mL dry Et_2O and N,N,N',N'-tetramethylethylendiamin (5.0 mL, 3.87 g, 34.0 mmol, 10 eq.) were added under argon. Then, the solution was cooled down to 0°C and *n*-butyl lithium (2.5 M in hexane, 13.6 mL, 34.0 mmol, 10 eq.) was added slowly. The yellowish solution obtained by this was stirred for 2 h at room temperature. In parallel, a solution of N,N-dimethylamino pyridine (12,44 g, 102.0 mmol, 30 eq.) and di-tert-butyl dicarbonate (18.42 g, 18.36 mL, 84.7 mmol, 25 eq.) in 300 mL dry Et_2O was prepared and stirred for 90 min at room temperature. Then, the solution of the lithiated trityl alcohol was transferred to the activated Boc_2O, whereupon the reaction mixture turned greenish and was stirred overnight in order to complete the carbonylation. After quenching with 300 mL water, the organic phase was separated and the aqueous one extracted with 100 mL Et_2O. The unified organic phases were dried over $MgSO_4$ and the solvent was removed. The yellow-orange crude product was purified by column chromatography on silica eluting with CyH/Et_2O (3:1, *v/v*) giving **11** as an orange solid (R_f = 0.38) in a yield of 1.88 g (16 mmol, 47 %). The analytical data is in accordance with the literature.[2]

2.2.6 Synthesis of **12**

Bis{[2,2,6,6-tetramethyl-8-(carboxy-tert-butyl)benzo[1,2-d;4,5-d]bis(1,3-dithiol)-4-yl]}-{[2,2,6,6-tetramethyl-8-(hydroxymethyl)benzo[1,2-d;4,5-d]bis(1,3-dithiol)-4-yl]}methanol

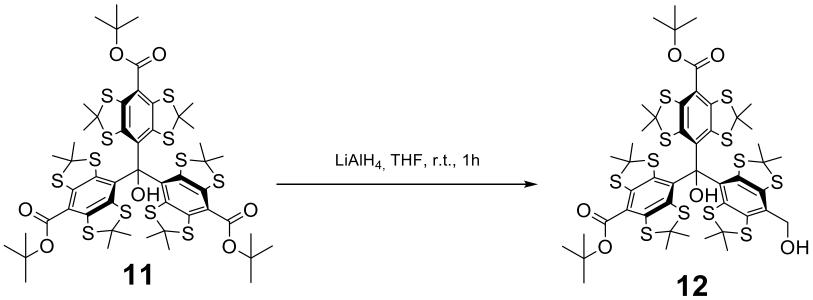

Trityl alcohol **11** (345 mg, 0.29 mmol) was dissolved in 15 mL THF, in parallel a solution of lithium aluminum hydride (170 mg, 4.47 mmol in 10 mL THF, 0.45 mmol mL^{-1}) was prepared. Then, the reduction was started by addition of 0.5 mL (0.23 mmol, 0.78 eq.) of the $LiAlH_4$ solution to the reaction mixture. After stirring for 5 min, the conversion was checked by TLC on SiO_2 eluting with CyH/EtOAc (2:1), where the product appears at R_f = 0.78 below the starting material. It was continued with a repetitive addition of 0.25 mL $LiAlH_4$-solution and evaluation by TLC after 5 min, until significant amounts of the twofold reduction product occur. Then, the reaction mixture was quenched by slow addition of 30 mL water and extracted with diethyl ether. The organic phase was separated and dried over $MgSO_4$, after removal of the solvents, an orange crude product was obtained, which was purified by column chromatography on silica eluting with CyH/EtOAc (2:1, *v/v)*. The product **12** was isolated as a yellow solid (R_f = 0.78) in a yield of 136 mg (42 %, 58 % brsm). Note, that the starting material (93 mg, 0.08 mmol, 27 mol%) could be recovered and used again in order to maximize the yield of this statistical transformation.

^{1}H-NMR (700 MHz, $CDCl_3$, 298 K, δ in ppm): 6.68 (s, 1H), 4.71 (d, $^2J_{H,H}$ = 12.4 Hz, 1H), 4.66 (d, $^2J_{H,H}$ = 12.4 Hz, 1H), 1.79 (s, 6H), 1.78 (s, 3H), 1.77 (s, 3H), 1.77 (s, 3H), 1.73 (s, 3H), 1.72 (s, 3H), 1.71 (s, 3H), 1.66 (s, 3H), 1.65 (s, 9H), 1.65 (s, 9H), 1.63 (s, 3H), 1.62 (s, 3H).

^{13}C{^{1}H}-NMR (176 MHz, $CDCl_3$, 298 K, δ in ppm): 165.58, 141.48, 141.43, 141.38, 141.05, 140.59, 140.50, 140.42, 140.20, 139.26, 138.53, 137.88, 137.72, 134.53, 134.42, 130.95, 130.56, 123.04, 122.98, 84.48, 84.39, 84.27, 66.04, 63.36, 62.57, 61.99, 61.07, 60.86, 60.83, 35.01, 34.86, 34.04, 33.36, 32.02, 31.17, 30.13, 29.69, 29.33, 28.55, 27.89, 27.86, 27.58.

HRMS (MALDI+, DCTB-matrix, *m/z,* $[M]^+$): calc. for $C_{48}H_{58}O_6S_{12}$, 1114.0876; found 1114.0884.

2.2.7 Synthesis of **14**

Bis{[2,2,6,6-tetramethyl-8-(carboxy-tert-butyl)benzo[1,2-d;4,5-d]bis(1,3-dithiol)-4-yl]}-{[2,2,6,6-tetramethyl-8-(1-methyl(3aRS,4SR,7RS,7aSR-4,7-epoxy-3,3a,7,7a-tetrahydro-2H-isoindoli-1,3-dione-2-yl)-(1[1,2-d;4,5-d]bis(1,3-dithiol)-4-yl]}methanol

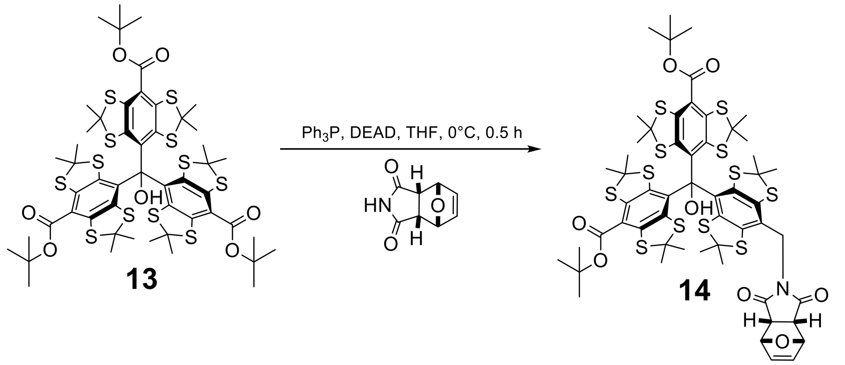

Trityl alcohol **12** (34 mg, 30.5 µmol), triphenyl phosphine (15 mg, 57.2 µmol, 1.87 eq.), and 3aRS,4SR,7RS,7aSR-4,7-epoxy-3,3a,7,7a-tetrahydro-2H-isoindoli-1,3-dione (10 mg, 60.6 µmol, 1.98 eq.) were dissolved in 1.5 mL dry THF under argon. Then, the reaction mixture was cooled down to 0 °C and 14 µL diethylazodicarboxylate (DEAD, 14 µL, 15.5 mg, 89.3 µmol, 2.92 eq.) were added, whereupon an immediate color change from orange to dark brown was observed. After stirring for 30 min at 0 °C, TLC (SiO_2, CyH/EtOAc 2:1, *v/v*) showed complete consumption of the starting material and water (10 mL) was added. The mixture was extracted with CH_2Cl_2 and the organic phase was separated and dried over $MgSO_4$. After removal of the solvents under reduced pressure, column chromatography on silica eluting with CyH/EtOAc (2:1 *v/v)* was performed in order to isolate the product (R_f = 0.43) as a yellow solid in a yield of 27 mg (70 %).

^{1}H-NMR (700 MHz, $CDCl_3$, 298 K, δ in ppm): 6.66 (s, 1H), 6.46 (dd, $^3J_{H,H}$ = 5.8, $^3J_{H,H}$ = 1.6 Hz, 1H), 6.43 (dd, $^3J_{H,H}$ = 5.9, $^3J_{H,H}$ = 1.6 Hz, 1H), 5.30 – 5.27 (m, 2H), 4.59 (d, $^2J_{H,H}$ = 14.6 Hz, 1H), 4.48 (d, $^2J_{H,H}$ = 14.6 Hz, 1H), 3.54 – 3.46 (m, 2H), 1.78 (s, 3H), 1.77 (s, 3H), 1.76 (s, 9H), 1.73 (s, 3H), 1.72 (s, 3H), 1.69 (s, 3H), 1.67 (s, 3H), 1.65 (s, 15H), 1.64 (s, 9H), 1.62 (s, 3H).

^{13}C{^{1}H}-NMR (176 MHz, $CDCl_3$, 298 K, δ in ppm): 173.90, 173.72, 165.60, 165.57, 141.51, 141.44, 141.36, 141.12, 140.72, 140.67, 140.53, 140.29, 139.33, 138.84, 138.57, 137.61, 134.97, 134.84, 134.50, 134.35, 130.65, 125.18, 123.02, 122.97, 84.47, 84.38, 84.28, 79.53, 63.39, 62.58, 62.14, 60.98, 60.88, 60.81, 46.15, 46.13, 44.00, 34.99, 34.92, 34.00, 33.27, 32.17, 31.41, 29.85, 29.81, 29.13, 28.55, 27.66, 27.54, 27.51.

HRMS (MALDI+, DCTB-matrix, *m/z,* [M-furane]$^+$): calc. for $C_{52}H_{59}NO_7S_{12}$, 1193.0935; found 1193.0922.

Note that **14** underwent Retro-Diels-Alder cleavage during mass-spectrometry, so that no M$^+$-peak was found neither in MALDI nor in ESI.

2.2.8 Synthesis of **15•**

Bis{[2,2,6,6-tetramethyl-8-carboxylbenzo[1,2-d;4,5-d]bis(1,3-dithiol)-4-yl]}-{[2,2,6,6-tetramethyl-8-(1-methyl(3aRS,4SR,7RS,7aSR-4,7-epoxy-3,3a,7,7a-tetrahydro-2H-isoindoli-1,3-dione-2-yl)-benzo[1,2-d;4,5-d]bis(1,3-dithiol)-4-yl]}methyl radical

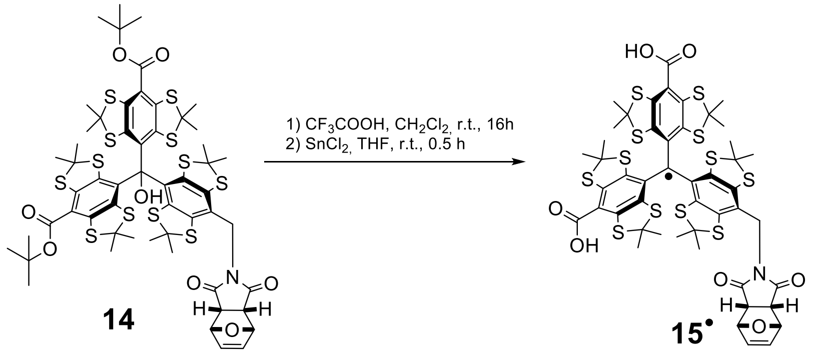

Trityl alcohol **14** (15 mg, 11.9 µmol) was dissolved in 100 µL dry CH_2Cl_2 under an argon atmosphere. Then, 2.0 mL CF_3COOH were added, whereupon the reaction mixture turned dark green. After stirring overnight, tin(II)-chloride (3.3 mg in 100 µL tetrahydrofurane, 17.8 mmol, 1.5 eq.) was added, whereupon a color change to brown was observable. After additional stirring for 15 min, all liquids were removed through high vacuum and the residue was taken up in 5 mL dichloromethane. The organic phase was washed with water, separated, and dried over $MgSO_4$. After removal of the solvents, a brown residue was obtained, which was purified by MPLC on RP-C18-silica eluting with CH_3CN/H_2O 4:1 (*v/v*). The first fraction was isolated and solvents were removed under reduced pressure obtaining **15** as a brown solid in a yield of 14 mg (95 %).
UHPLC (60 % CH_3CN, 40 % H_2O; each with 0.05 % CF_3COOH; t_r): 1.81 min
HRMS (MALDI+, DCTB-matrix, *m/z,* $[M]^+$): calc. for $C_{48}H_{46}NO_7S_{12}$, 1131.9917; found 1131.9906
cw-EPR (X-Band, DMSO, 200 µM, 298 K): g-value = 2.0034; A_N = 1.51 MHz, A_H = 3.39 MHz, A_H = 5.16 MHz, $A_{C,central}$ = 66.10 MHz, $A_{C,ipso}$ = 31.11 MHz, $A_{C,ortho}$ = 25.43 MHz, $A_{C,para}$ = 6.83 MHz, $A_{C,meta}$ = 3.51 MHz.

2.2.9 Synthesis of **9•**

Bis{[2,2,6,6-tetramethyl-8-carboxylbenzo[1,2-d;4,5-d]bis(1,3-dithiol)-4-yl]}-{[2,2,6,6-tetramethyl-8-(1-methyl(3,4-dehydropyrolidin-1,3-dione-2-yl)-benzo[1,2-d;4,5-d]bis(1,3-dithiol)-4-yl]}methyl radical

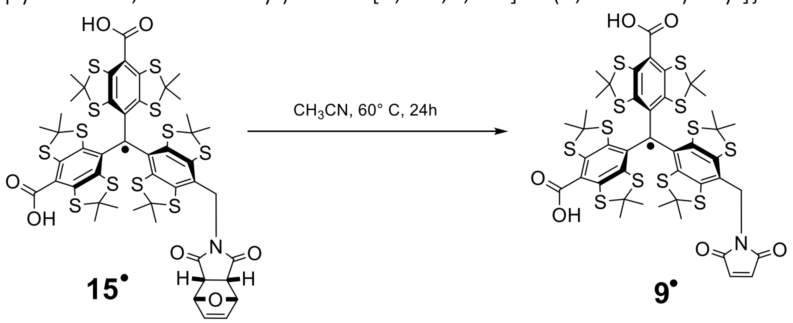

Trityl radical 15• (10 mg, 8.8 µmol) was suspended in 5 mL dry degassed acetonitrile and stirred at 60 °C for 24 h under argon atmosphere. All volatiles were removed in high vacuum and the product was obtained as a brown solid in quantitative yield of 9 mg.
UHPLC (60% CH_3CN, 40% H_2O; each with 0.05% CF_3COOH; t_r): 2.04 min
HRMS (MALDI+, DCTB-matrix, *m/z,* $[M\text{-furane}]^+$): calc. for $C_{44}H_{42}NO_6S_{12}$, 1063.966; found 1063.968
cw-EPR (X-Band, DMSO, 200 µM, 298 K): g-value = 2.0034; A_N = 1.48 MHz, A_H = 3.48 MHz, A_H = 6.06 MHz, $A_{C,central}$ = 66.16 MHz, $A_{C,ipso}$ = 31.22 MHz, $A_{C,ortho}$ = 25.45 MHz, $A_{C,para}$ = 6.86 MHz, $A_{C,meta}$ = 3.57 MHz.

2.3 Analytical Data Synthesis

2.3.1 NMR Spectra

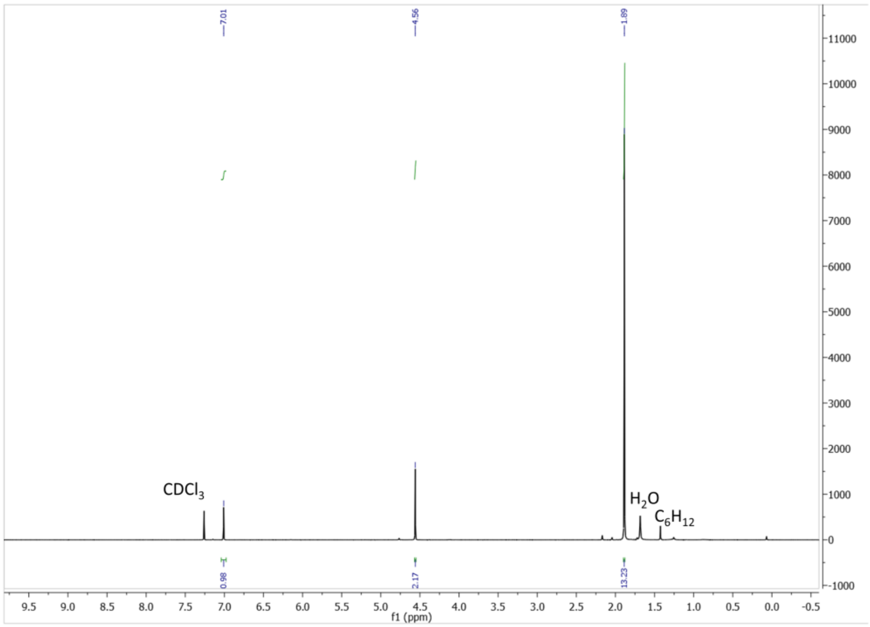

,

Figure S2: *^{1}H-NMR (500 MHz, 298 K, $CDCl_3$) of* ***S2.***

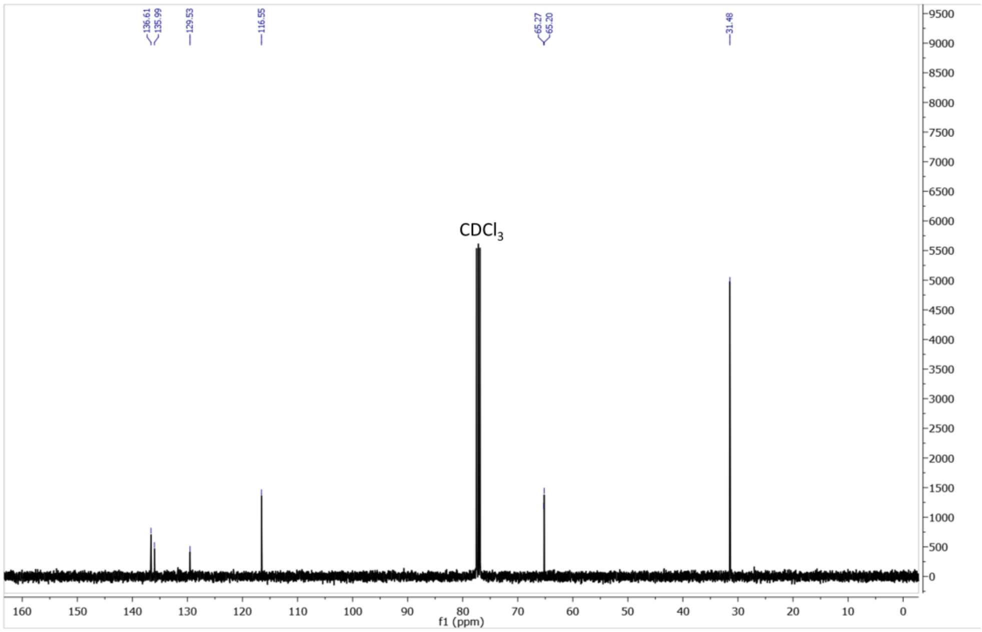

Figure S3: *^{13}C{^{1}H}-NMR (125 MHz, 298 K, $CDCl_3$) of* ***S2***

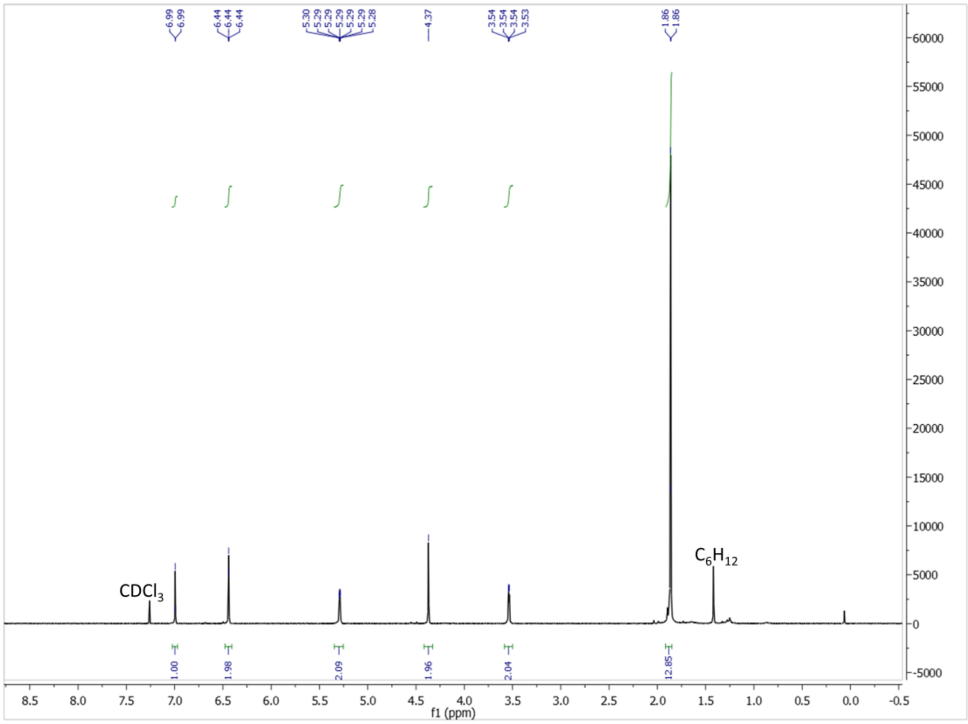

Figure S4: *^{1}H-NMR (500 MHz, 298 K, CDCl$_3$) of* ***S3.***

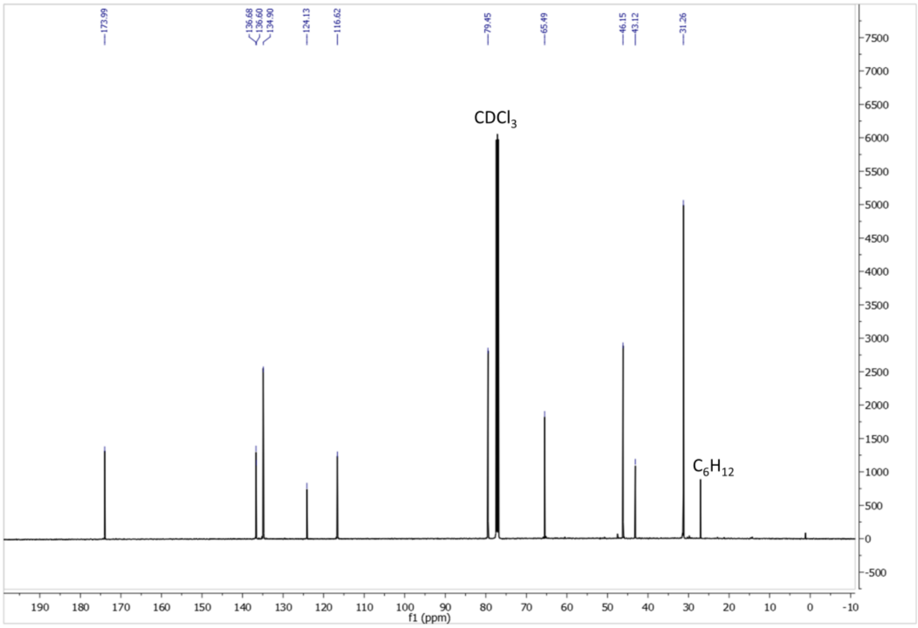

Figure S5: *^{13}C{^{1}H}-NMR (125 MHz, 298 K, CDCl$_3$) of* ***S3.***

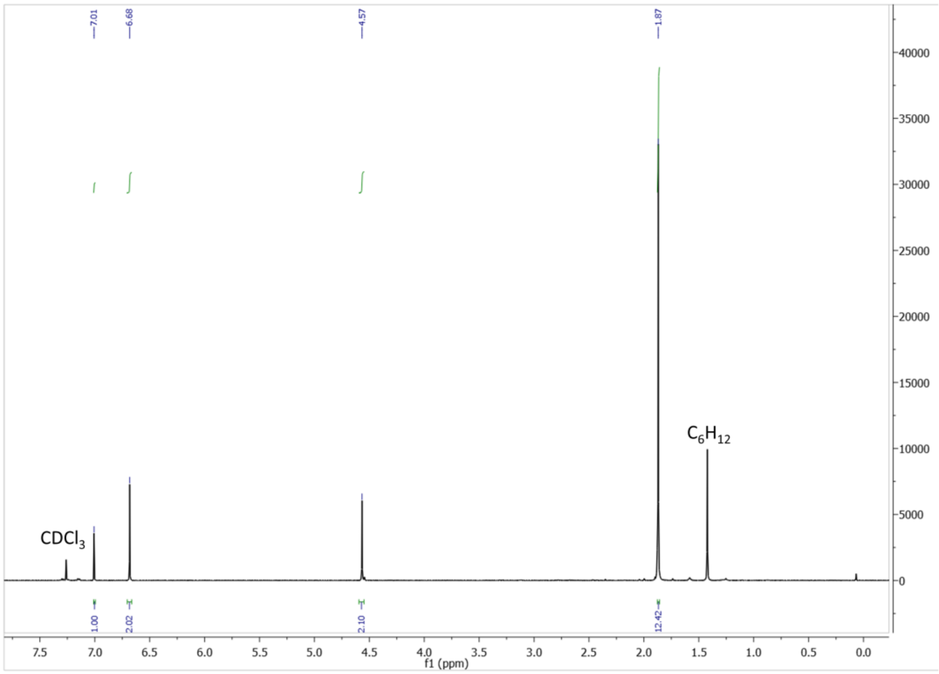

Figure S6: *^{1}H-NMR (500 MHz, 298 K, $CDCl_3$) of* ***S4.***

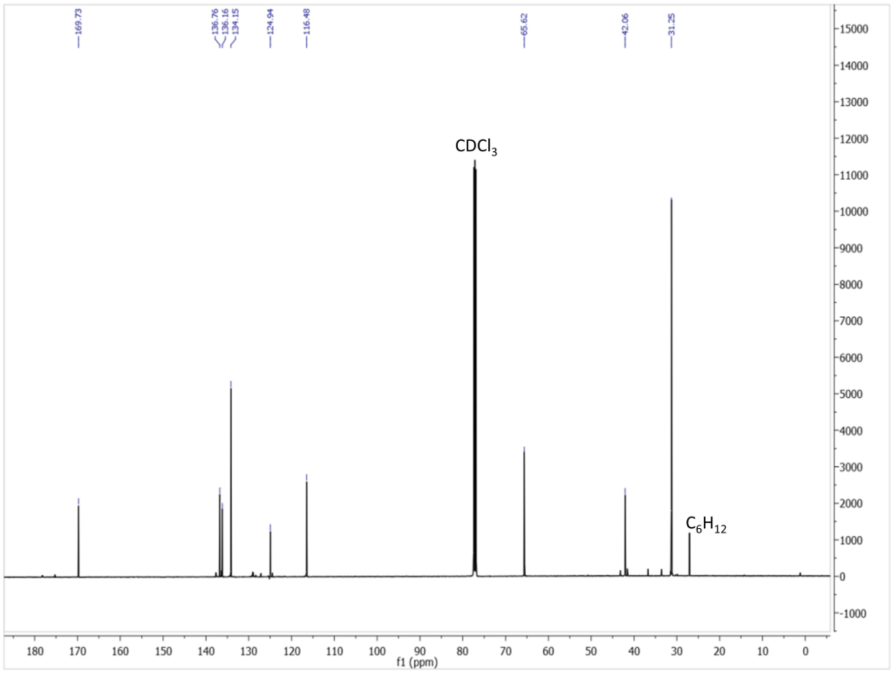

Figure S7: *^{13}C{^{1}H}-NMR (125 MHz, 298 K, $CDCl_3$) of* ***S4.***

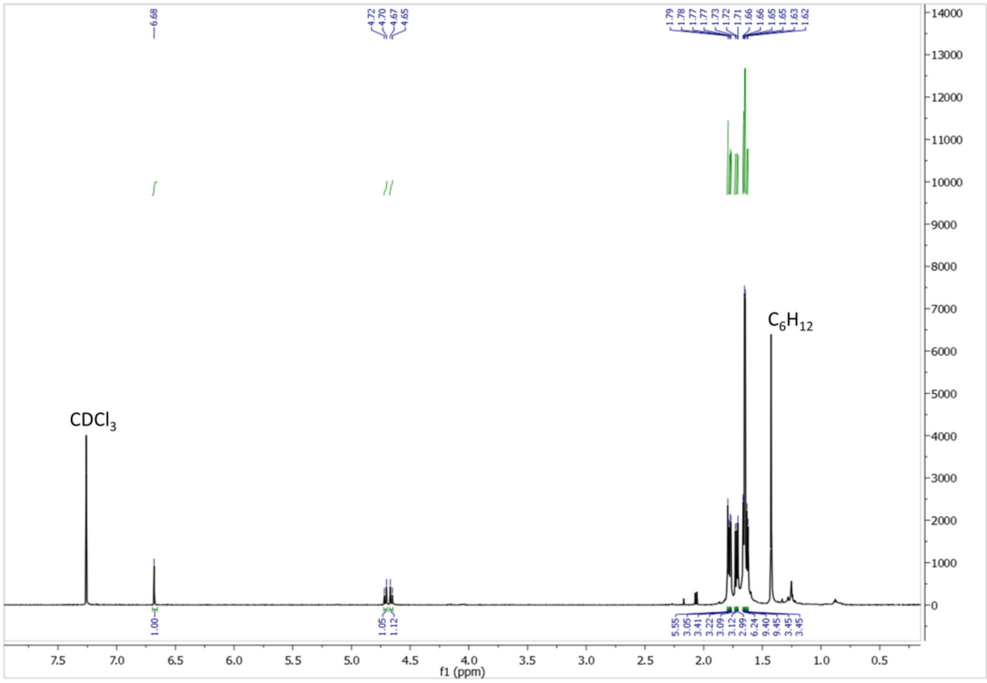

Figure S8: *^{1}H-NMR (700 MHz, 298 K, CDCl$_3$) of* ***12****.*

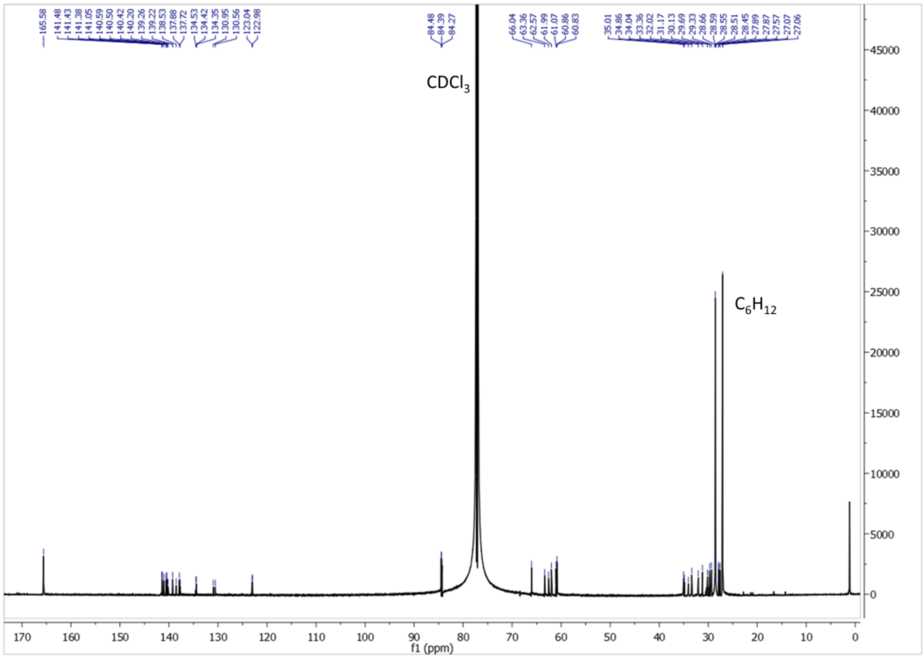

Figure S9: *^{13}C{^{1}H}-NMR (176 MHz, 298 K, CDCl$_3$) of* ***12****.*

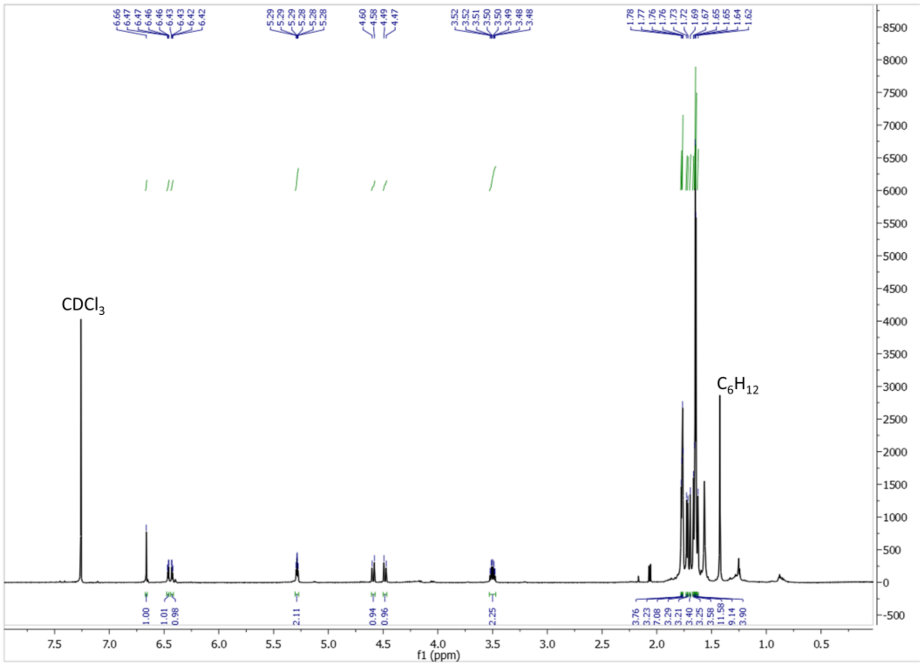

Figure S10: *^{1}H-NMR (700 MHz, 298 K, CDCl$_3$) of* ***14****.*

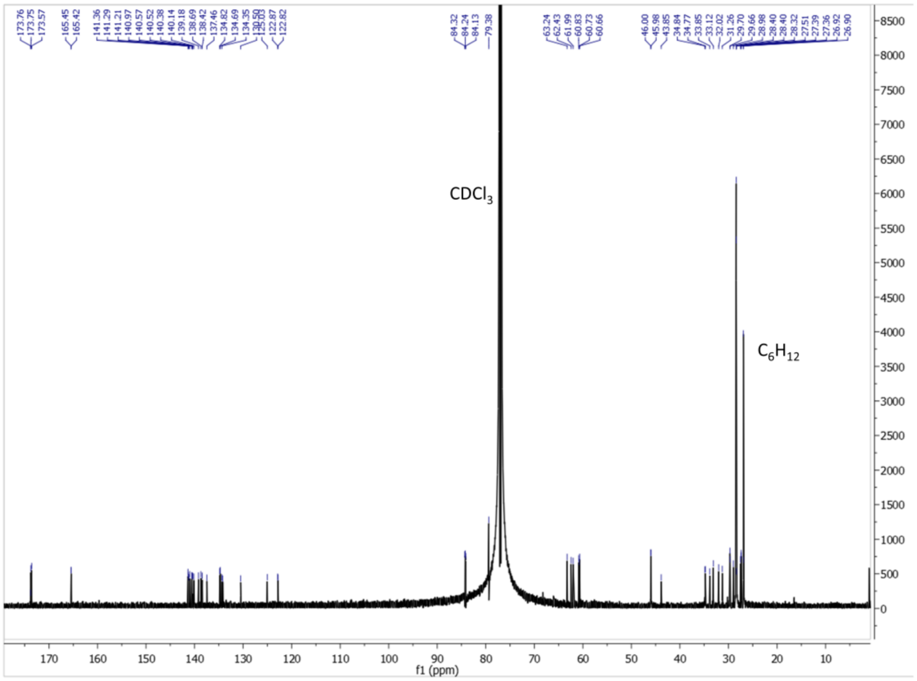

Figure S11: *^{13}C{^{1}H}-NMR (176 MHz, 298 K, CDCl$_3$) of* ***14****.*

2.3.2 HRMS-Data

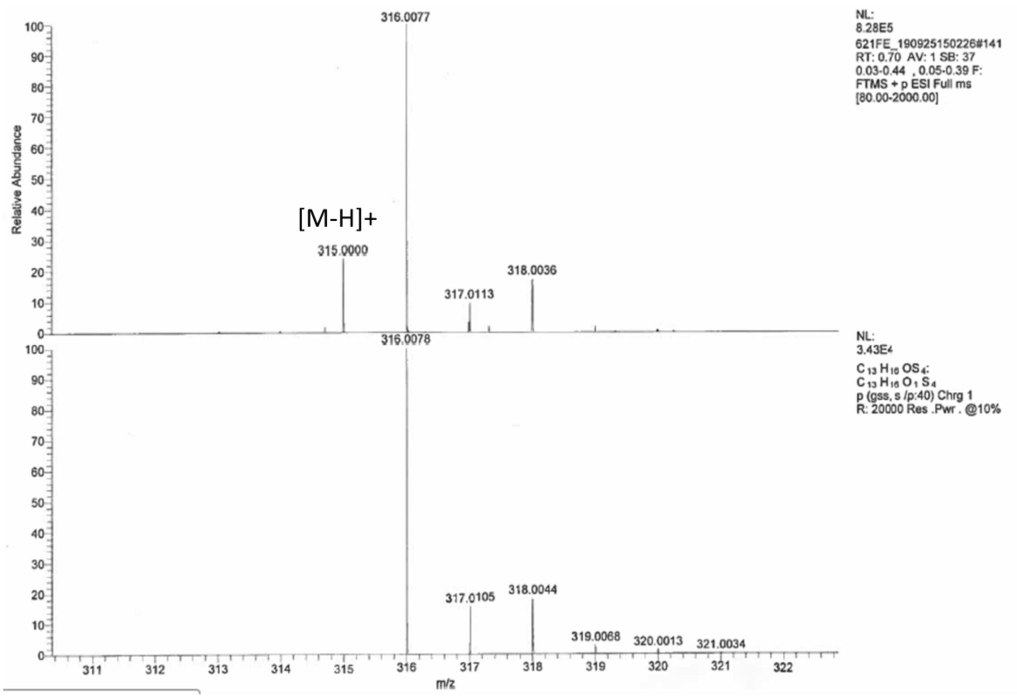

Figure S12: *ESI(+)-HRMS (top) of **S2** and calculated isotope pattern for M$^+$ (bottom).*

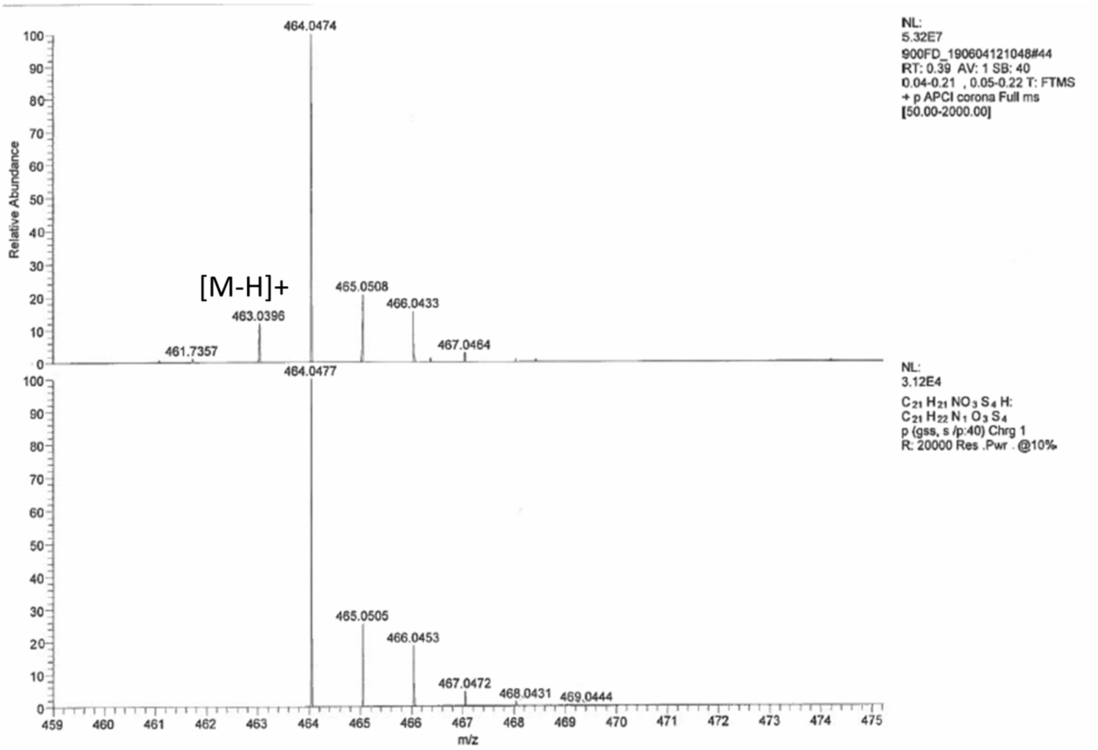

Figure S13: *APCI-HRMS (top) of **S3** and calculated isotope pattern for [M+H]$^+$ (bottom).*

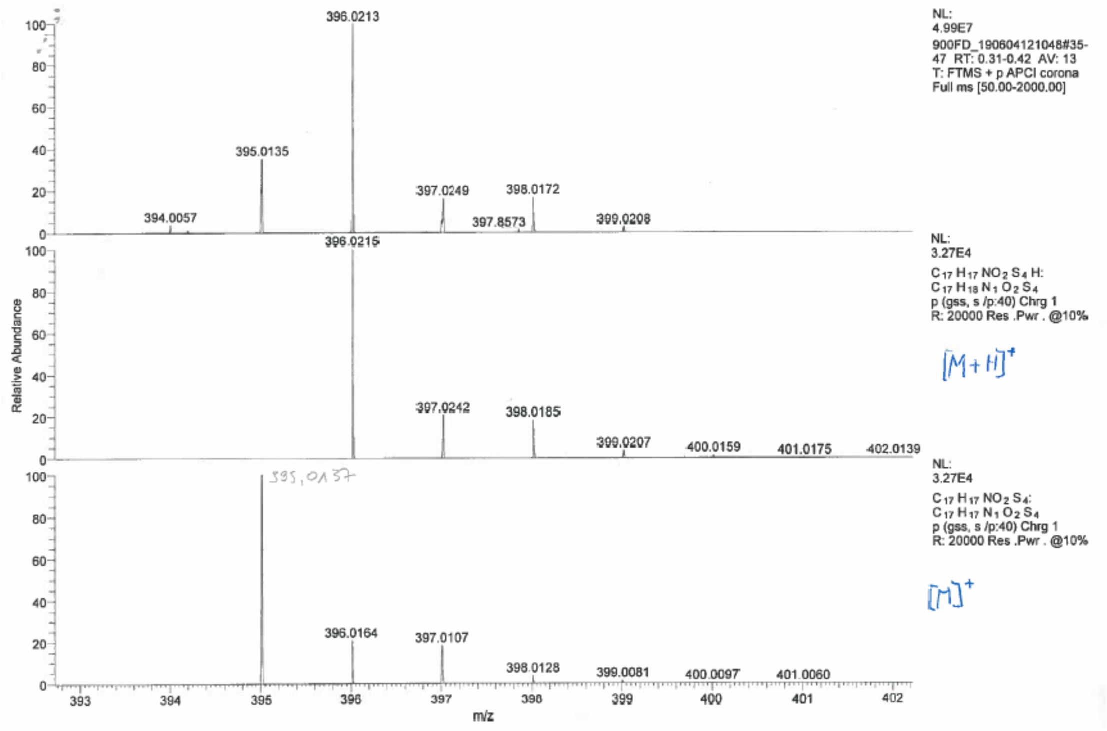

Figure S14: *APCI-HRMS (top) of **S4** and calculated isotope patterns (bottom).*

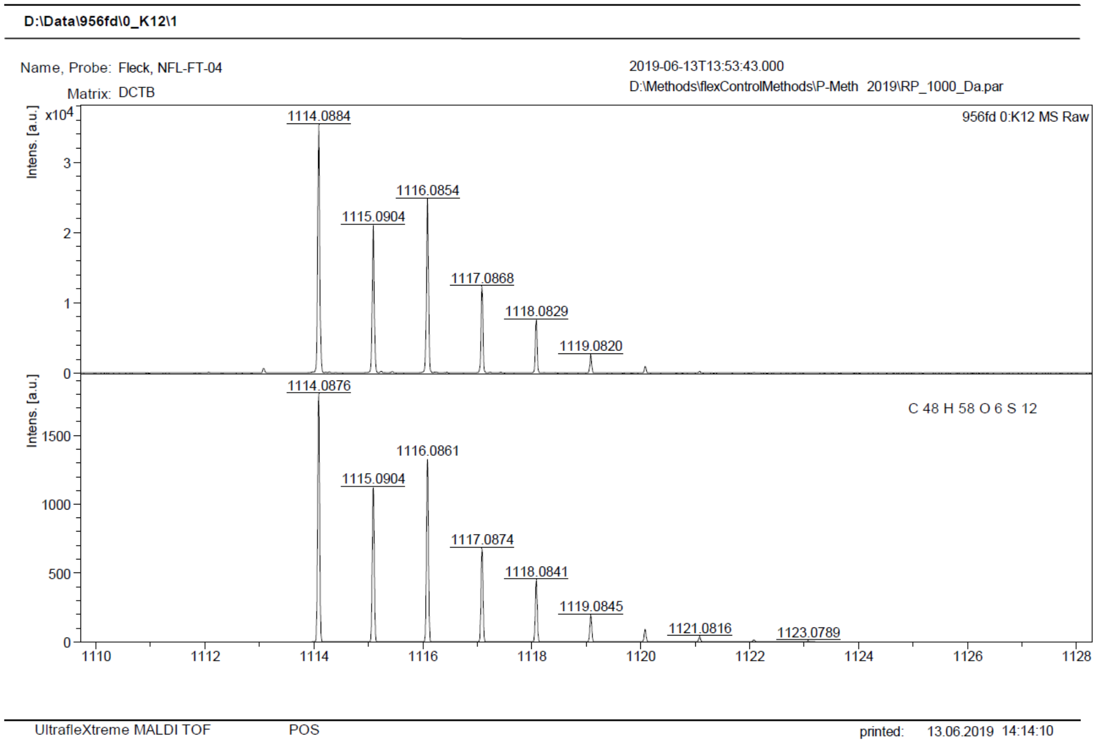

Figure S15: *MALDI(+)-HRMS (top) of **12** and calculated isotope pattern for M+ (bottom).*

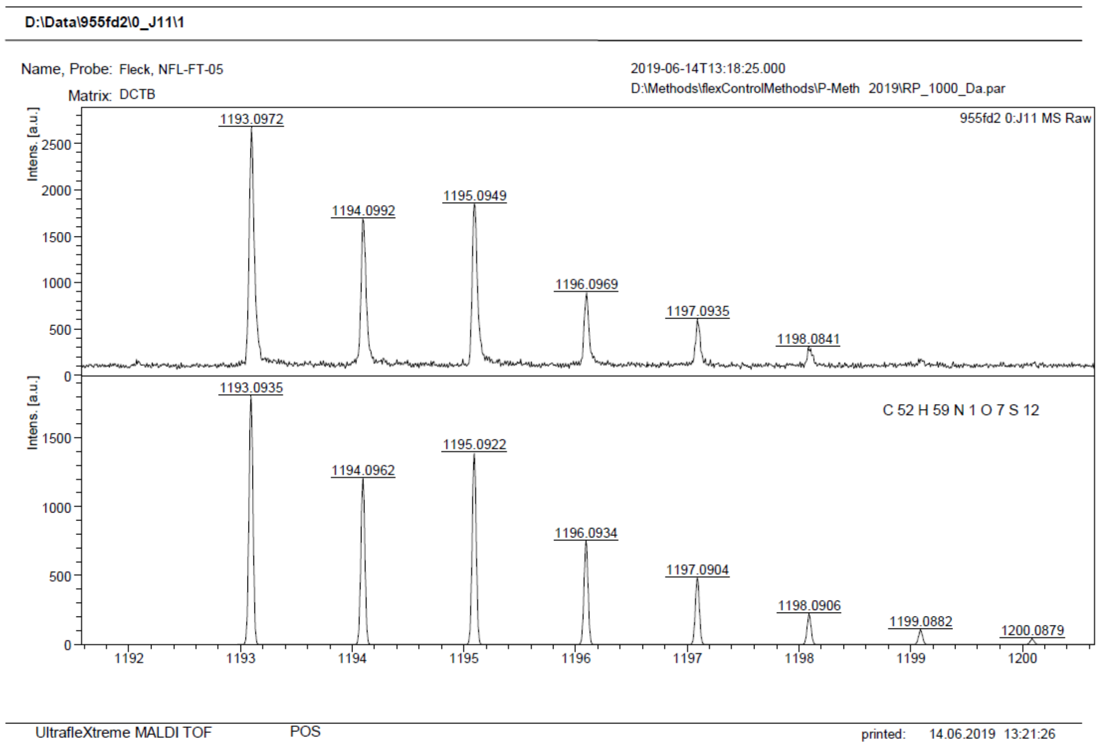

Figure S16: *MALDI(+)-HRMS (top) of* ***14*** *and calculated isotope pattern for M*$^+$ *(bottom).*

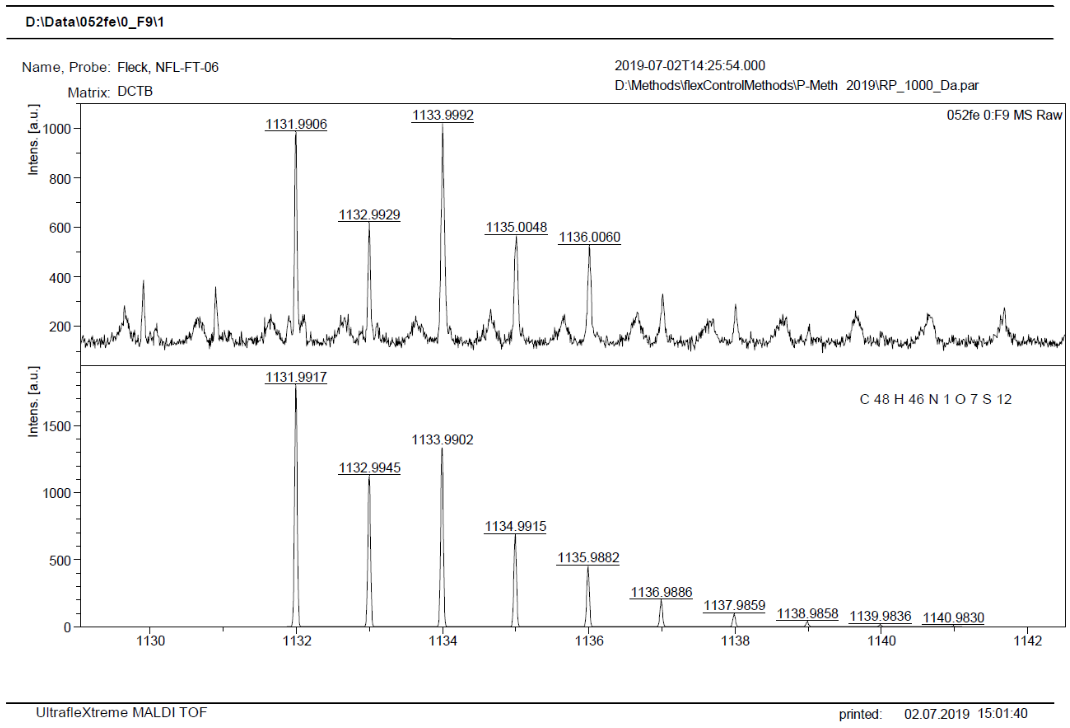

Figure S17: *MALDI(+)-HRMS (top) of* ***15•*** *and calculated isotope pattern for M*$^+$ *(bottom). Note, that the [M]*$^+$*-peak shown here is not the base peak due to Retro-Diels-Alder fragmentation in the mass spectrometer and therefore suffers from low intensity. The regularly appearing noisy peaks belong to the spectrometer background.*

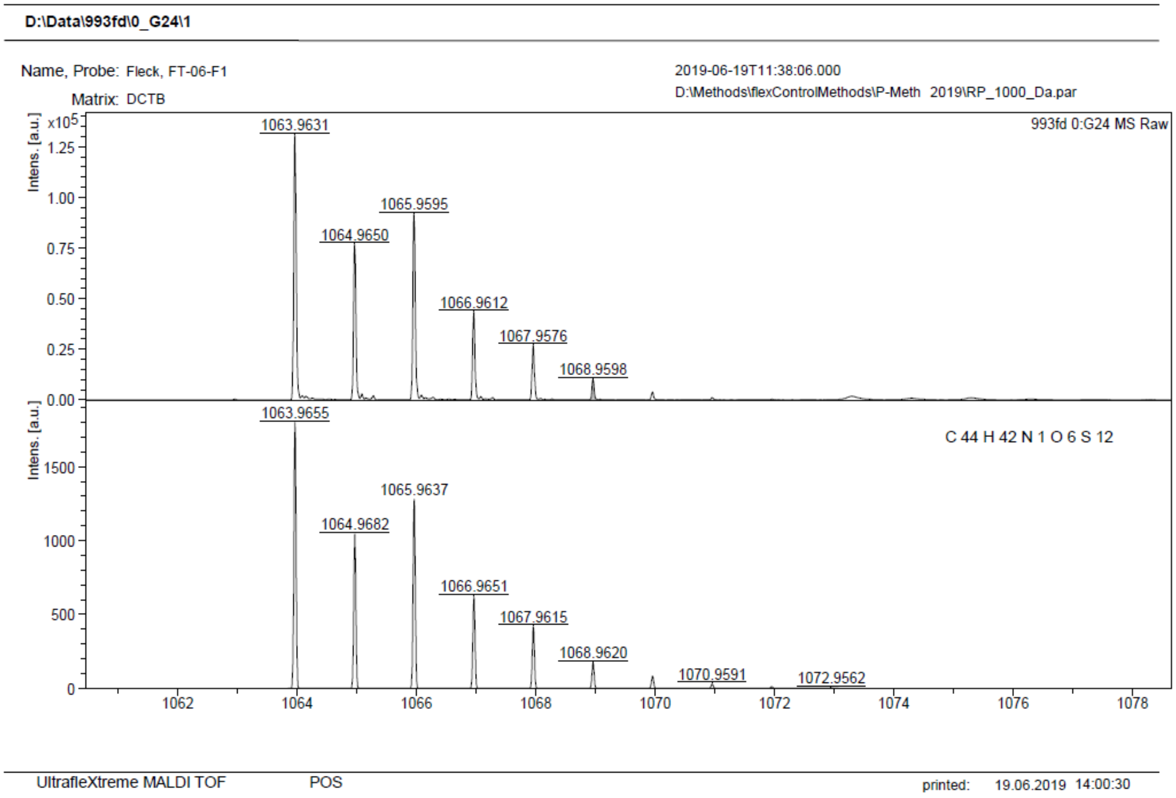

Figure S18: *MALDI(+)-HRMS (top) of **9•** and calculated isotope pattern for M⁺ (bottom).*

2.3.3 UHPLC Data

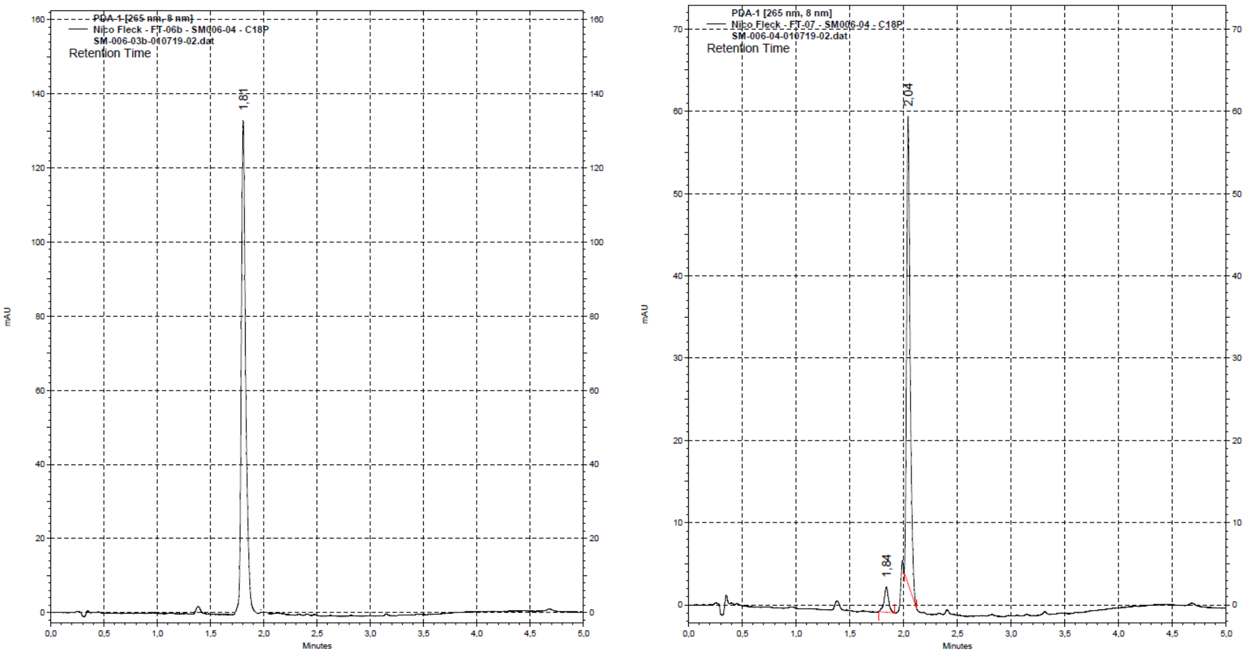

Figure S19: *UHPLC chromatogram of **15•** (left) and of **9•** (right).*

3. Spin Labeling

3.1 Tetraethylnitroxide-DNA Construct

Since the isoindoline based tetraethylnitroxide **S5** does not possess sufficient solubility in aqueous environments, it was conjugated to a DNA strand. This yielded a water-soluble construct, which is suitable for stability measurements.

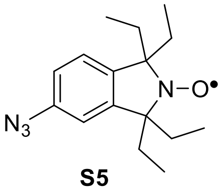

S5

For this, a DNA strand (5’ GGG TGX CTG GTA CCC 3’, obtained from *Metabion,* X = 5-ethynyl-2’dU) was labeled with **S5**[7] using our recently published protocol.[8] Annealing of the labeled strand with the complementary unmodified strand provided the singly labeled DNA duplex used for the stability studies.

3.2 SLIM Dilution Series and Calibration Curve

To create a stock solution of **9•** for subsequent protein labeling, the free label **9•** was dissolved in DMSO. Since potential weighing errors may have severe impact on the correctness of all following quantification routines, five independent EPR spin count experiments with the DMSO stock diluted 1:100 in POi buffer (20 mM POi pH 6.8, 50 mM NaCl) were performed on an EMXnano EPR spectrometer. The final concentration based on the mean value of these measurements accounted to 2.58 mM of **9•**.
A dilution series of **9•** in POi buffer was set up and measured on a Cary 100 UV-vis spectrometer (Agilent Technologies, Santa Clara, CA, USA) and the molar extinction coefficients of **9•** at 271 nm and 459 nm were determined via linearization according to the *Lambert-Beer law*.

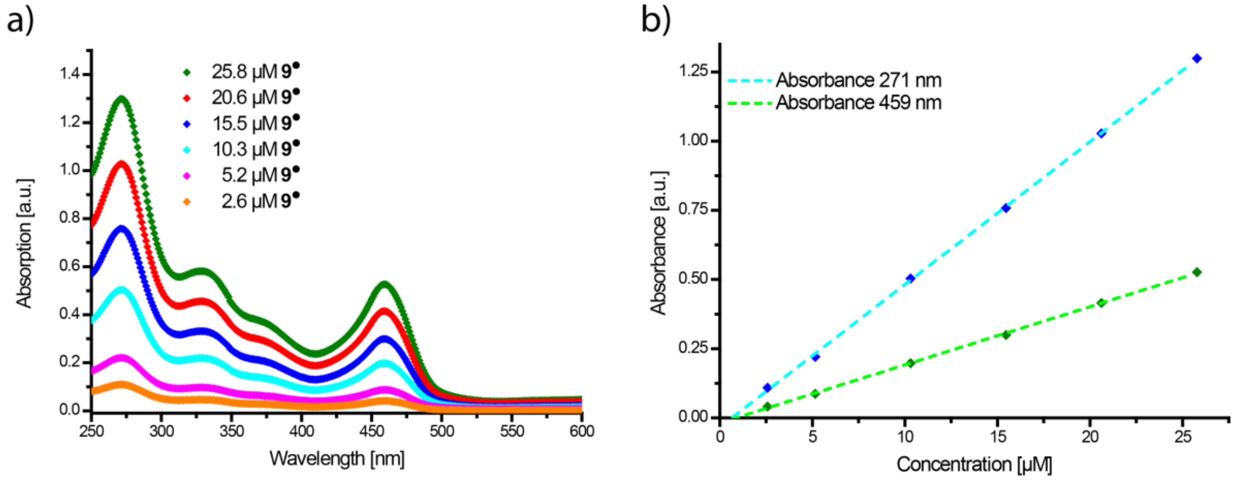

Figure S20: *UV-vis dilution series of **9•** (a) and the linear fits of the absorbance at 271 nm (cyan) and 459 nm (green) (b).*

Table S1*: Extinction coefficients of* ***9•*** *at 271 nm and 471 nm.*

Wavelength [nm]	Extinction coefficient **ε** $\left[\frac{L}{\mu mol \cdot cm}\right]$
271	0.05152
459	0.02099

The labeling efficiencies of YopO in each labeling experiment using trityls **9•** or **8•** were determined using the previously implemented technique.[9] Since the protein itself only absorbs at ~280 nm and both trityl labels show a second absorption maximum at 459 nm (**9•**) or 467 nm (**8•**), the protein and respective trityl concentrations can be determined as follows:

$$c_{\text{trityl}} = \frac{A_{459/467}}{d \cdot \varepsilon_{\text{trityl}}}$$

$$c_{\text{YopO}} = \frac{A_{271} - c_{\text{trityl}} \cdot d \cdot \varepsilon_{271,\text{trityl}}}{d \cdot \varepsilon_{280,\text{YopO}}}$$

Although slight shifts in the absorption maxima wavelengths are seen for both trityl labels upon bioconjugation, the errors resulting from these shifts are negligible. The extinction coefficient of YopO was calculated using the computing tool ProtParam.[10] All utilized extinction coefficients are listed in Table S2.

Table S2*: Extinction coefficients of YopO,* ***8•*** *and* ***9•****.*

Protein/Trityl	**ε** $\left[\frac{L}{\mu mol \cdot cm}\right]$
YopO	ε_{280} = 0.04939
**9•**	ε_{271} = 0.05152
	ε_{459} = 0.02099
**8•**	ε_{280} = 0.01930
	ε_{467} = 0.00750

3.3 *Yersinia* outer protein O (YopO)

3.3.1 Construct Design

Truncated YopO$_{89\text{-}729}$ C219A (YopO-WT) from *Yersinia enterocolitica* was cloned in frame into the pGex6p1 vector (GE Healthcare, Chicago, IL, USA), transformed into *E.coli* DH5α cells and amplified. This construct served as the template for further mutagenesis and cysteine introduction via *QuickChange* mutagenesis[11] for YopO N624C and YopO Y588C/N624C. The primers are listed in Table S3.

Table S3*: YopO primer pairs*

	Sequence
Y588C *fwd*	5'-CAAGGGCAGCCCGTGTCCTCTGAAACCTGTAGCTTCC-3'
Y588C *rev*	5'-GACCTTAGCCTCAGCTAATCGATTCAGGAAGCTACAGGTTTCAG-3'
N624c *fwd*	5'-GAGAGTGCGAAAGCGCAACTATCTATTCTGATTTGTCGTTCAG-3'
N624C *rev*	5'-GAGCAACATCAGCCCAAGAACCTGAACGACAAATCAGAATAG-3'

After PCR, the new constructs were transformed into *E.coli* DH5α cells and the successful mutagenesis was confirmed *via* Sanger sequencing.

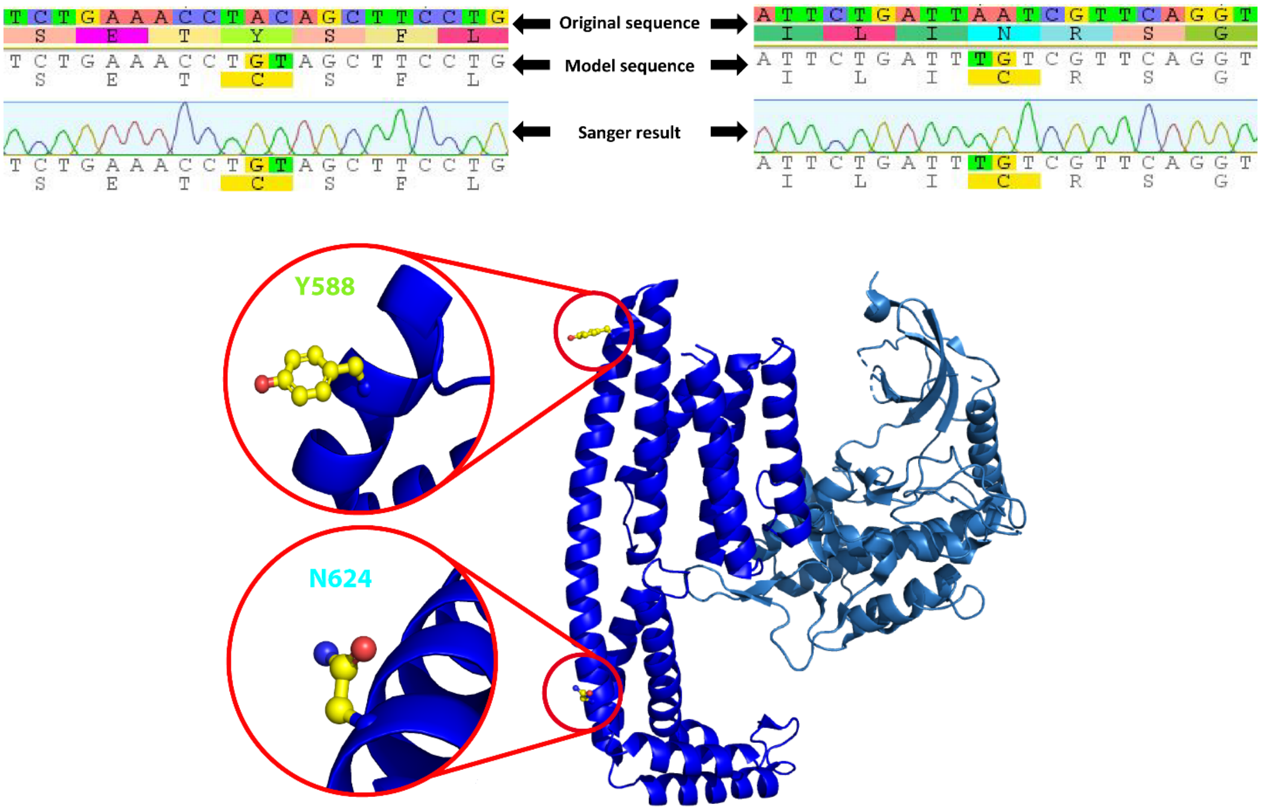

Figure S21: *Sequencing results for YopO Y585C/N624C (top, excerpt taken from Geneious (Biomatters Ltd., Auckland, New Zealand)). PyMOL (Schrödinger, New York, NY, USA) representation showing the locations of the unmutated amino acid positions in YopO (PDB-ID: 4ci6) (bottom).*

3.3.2 YopO Expression

All YopO constructs were expressed in *E.coli* Rosetta (DE3) cells. From an agar plate, a single colony was picked and grown overnight at 37 °C / 180 rpm in 20 mL LB medium containing 0.3 mM ampicillin and 0.1 mM chloramphenicol. Main-cultures were set up in 1 L LB medium (0.3 mM ampicillin, 0.1 mM chloramphenicol) using 15 mL pre-culture and were incubated at 37 °C / 180 rpm until an OD_{600} of ~0.8 – 1.0 was reached. Protein expression was induced upon the addition of 0.1 mM IPTG (isopropyl β-D-1-thioglactopyranoside) and the cultures were incubated for ~16 h at 16 °C before being harvested after centrifugal separation (4000 rcf, 20 min, 4 °C).

3.3.3 YopO Purification

The cell pellet was re-suspended in 5 time v/w lysis buffer (50 mM Tris-Cl pH 8.0, 150 mM NaCl, 3 mM DTT), lysed twice in a cell disruptor (Constant Systems Limited, Northampton, UK) and the lysate was spun down to remove the insoluble cell debris (48,500 rcf, 20 min, 4 °C). GST sepharose beads were equilibrated with lysis buffer and incubated with the centrifugal supernatant for 1 h at room temperature under slight agitation. The GST-suspension was ran over a gravity column and the flow-through was passed over the settled beads an additional time to maximize the protein yield. The beads were washed with 50 mL of lysis buffer and then incubated in 20 mL elution buffer (50 mM Tris-Cl pH 8.0, 150 mM NaCl, 1 mM DTT, 100 U PreScission protease) overnight at 4 °C whilst slowly shaking. The suspension was filled back into the gravity column and the protein containing flow-through was diluted with 100 mL no-salt buffer (50 mM Tris-Cl pH 8.0) and 125 µL of a 2 M DTT stock were added. An ion-exchange chromatography on an EnrichQ 10/100

column (Bio-Rad Laboratories GmbH, Feldkirchen, Germany) was performed against a linear gradient of high-salt buffer (50 mM Tris-Cl pH 8.0, 1 M NaCl) and the protein containing fractions were pooled and concentrated down below 3.0 mL. The sample was loaded onto a HiLoad Superdex 200 16/600 (GE Healthcare, Chicago, IL, USA) and size-exclusion chromatography was performed in gel filtration buffer (50 mM Tris pH 8.0, 50 mM NaCl). Pure protein (based on SDS-PAGE) was pooled, flash frozen and stored in 100 µL aliquots at –80 °C.

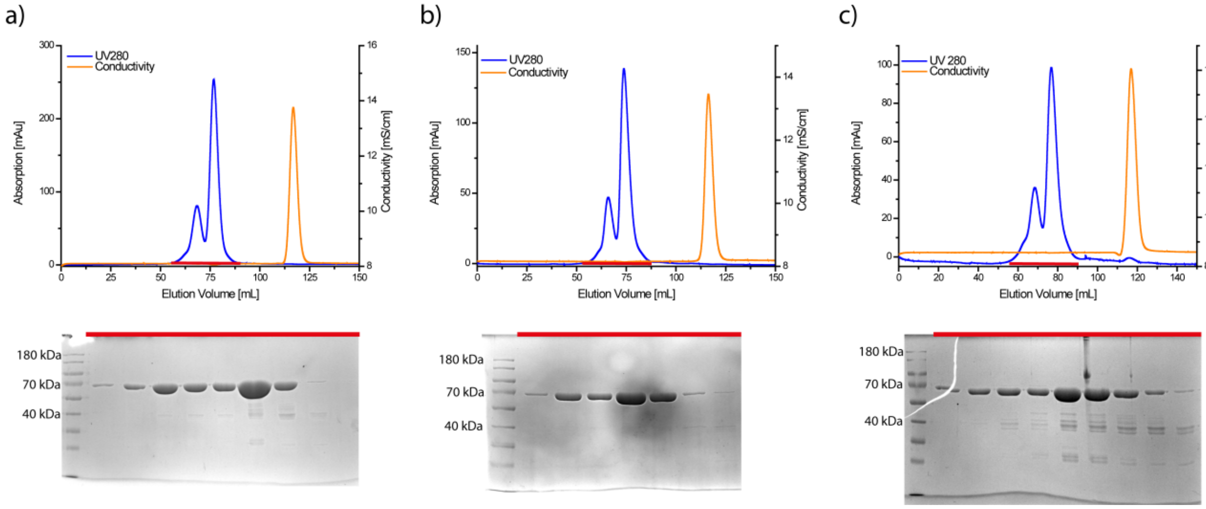

Figure S22: *HiLoad Superdex 200 16/600 gel filtration chromatographs of YopO 0-Cys (a), YopO N624C (b) and YopO Y588C/N624C (c) (top) and the corresponding SDS-PAGE gels of the indicated peaks (red) in the elugram. Note that in each gel filtration chromatograph two distinct absorption peaks are seen with the first one corresponding to non-covalent, homomeric YopO dimers (~140 kDa) and the latter to monomeric YopO proteins (~70 kDa) as also proven by non-reducing SDS-PAGE (not shown here).*

3.3.4 YopO Spin Labeling

Prior to each labeling experiment, 20 nmol of YopO were incubated for 1.5 h at 4 °C in 2.5 mL phosphate buffer (20 mM POi pH 6.8, 50 mM NaCl) containing a five-fold molar excess of TCEP to cleave any YopO multimers. The reducing agent was removed using a PD-10 desalting column and labeling reactions were set up immediately afterwards.

To the 3.5 mL PD-10 protein eluate, a 5-fold molar excess per cysteine (or 5-fold molar excess per YopO for the YopO-WT C219A construct) of SLIM **9•** pre-diluted in 3 mL POi buffer was added resulting in a final concentration of 3.1 µM YopO and 31 µM SLIM trityl respectively. The labeling reaction was incubated for 16 h at 4 °C. Next, the incubation solution was split into five equal fractions of 1.3 mL and passed successively over a PD-10 desalting column using the protocol for volumes less than 2.5 mL to remove any unbound free label remnants. All PD-10 eluate fractions were pooled and concentrated using a VivaSpin 6/50k MWCO (Sartorius, Göttingen, Germany) to less than 2.5 mL. For enhanced separation of any aggregation seeds or unbound label, the concentrated fractions were additionally purified using a HiPrep 26/10 desalting column (GE Healthcare, Chicago, IL, USA). Protein containing fractions were pooled, concentrated down to ~3.0 mL and a UV-vis spectrum was recorded on a Cary 100 UV-vis spectrometer. The labeling efficiencies were calculated as described in SI section 3.2.

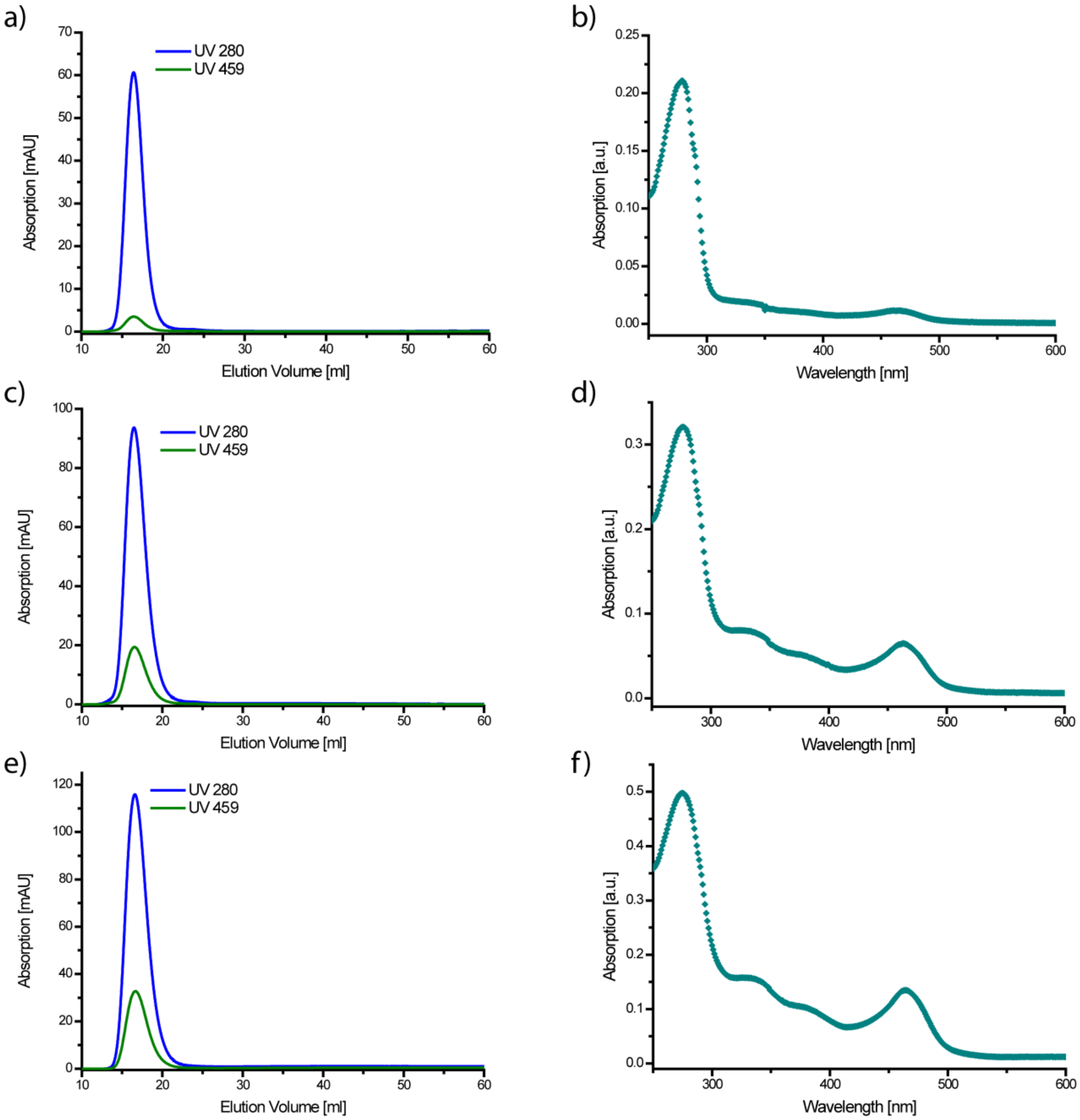

Figure S23*: Elution chromatographs of the HiPrep 26/10 runs and UV-vis spectra for YopO 0-Cys (a+b), YopO N624C (c+d) and YopO Y588C/N624C (e+f).*

Table S4*: Labeling efficiencies for YopO labeled with SLIM.*

Construct	Eq. SLIM per protein	Labeling efficiency [%]
YopO-WT	0.071 ± 0.006	7.1 ± 0.6
YopO N624C	0.955 ± 0.088	95.5 ± 8.8
YopO Y588C/N624C	1.916 ± 0.162	95.8 ± 8.8

The YopO Y588C/N624C reference sample labeled with **8•** were generated in exactly the same manner as the SLIM trityl samples, only using **8•** as the trityl species instead. For the **MTSL** reference sample, 20 nmol YopO were incubated with 3 mM DTT in 2.5 mL gel filtration buffer (50 mM Tris-Cl pH 8.0, 50 mM NaCl) for 2 h before removing the reducing agent via a PD-10 desalting column. Labeling reactions were set up

immediately afterwards by addition of a ten-fold molar excess of **MTSL** per cysteine. The labeling solution was incubated for 16 h at 4 °C. Excess free label was removed via a PD-10 desalting column and the pooled protein fractions were concentrated using a VivaSpin 6/10k MWCO.

All samples that were subjected to *in vitro* pulsed EPR experiments were rebuffered thrice in deuterated PDS buffer (100 mM TES pH 7.4, 100 mM NaCl) using a VivaSpin 6/50k MWCO.

3.3.5 YopO Mass Spectrometry

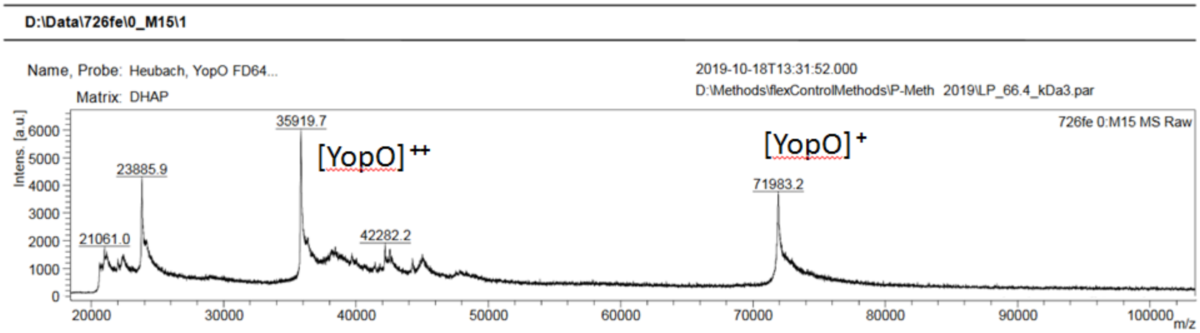

Figure S24: *MALDI(+)-spectrum of YopO-WT after incubation with SLIM **9•**.*

Figure S24 shows the MALDI(+)-spectrum of YopO-WT after incubation with SLIM as described beforehand. While the peak for the unlabeled protein at 71,983 Da (calc. 72,108 for $C_{3176}H_{5085}N_{897}O_{989}S_{14}$) appears very clear, no signal of unspecifically labeled protein occurs, which would be at least 1,063 Da heavier.

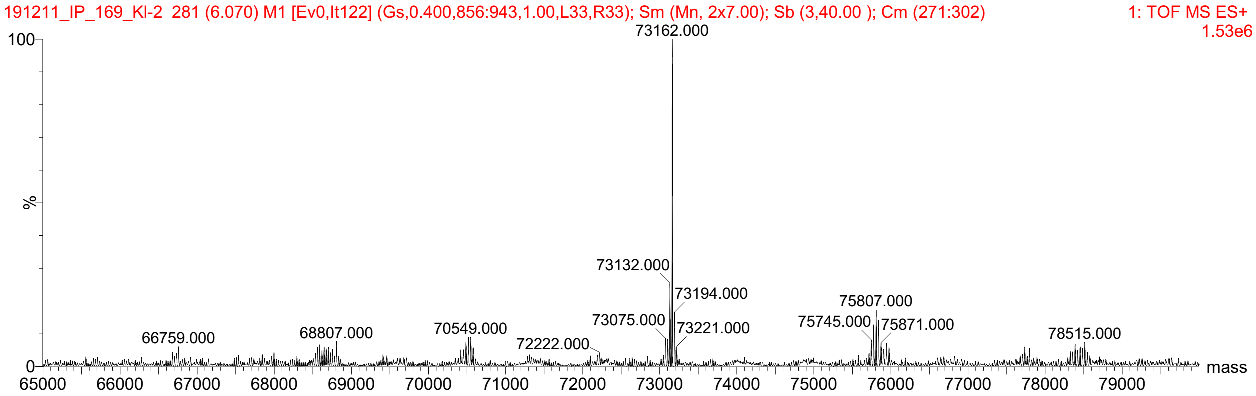

Figure S25: *High-resolution ESI(+) of YopO N624C labeled with SLIM **9•**.*

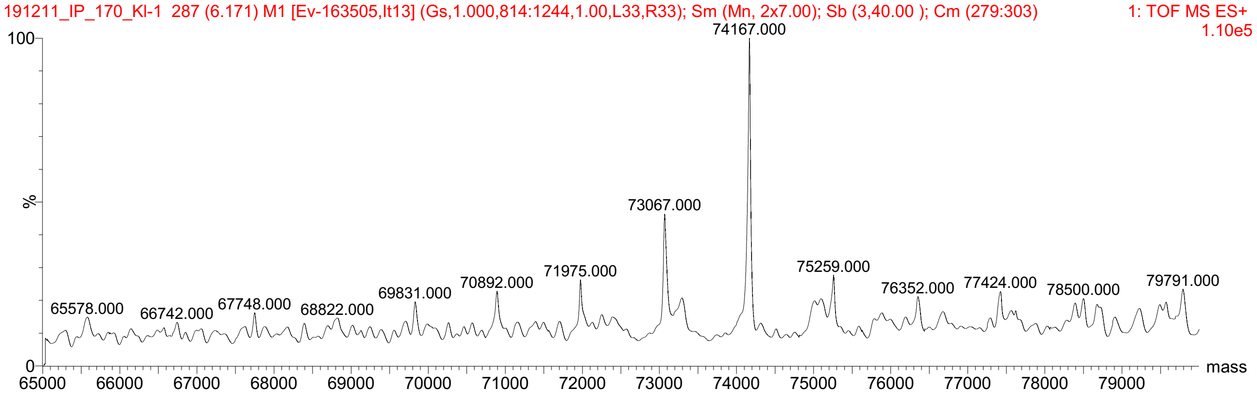

__Figure S26__: High-resolution ESI(+) of YopO Y588C/N624C labeled with SLIM __9•__.

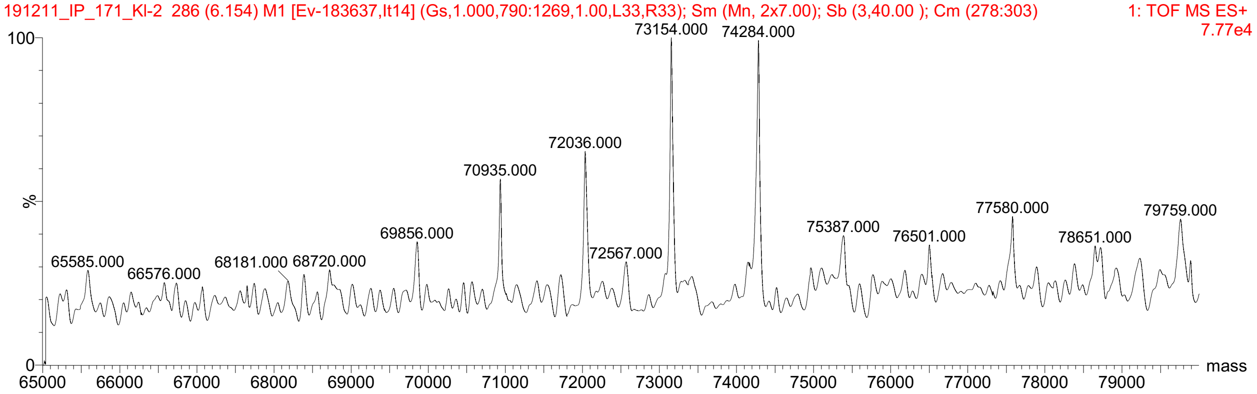

__Figure S27__: High-resolution ESI(+) of YopO Y588C/N624C labeled with __8•__.

The identity of the labeled proteins was confirmed by high-resolution ESI(+) mass spectrometry as shown in Figures S25 – S27. These spectra clearly prove the chemoselective and successful labeling of the YopO mutants within in an accuracy exceeding 15 ppm.

__Table S5__: Calculated and measured masses for YopO mutants

Mutant	Sum formula	Calc. mass [Da]	Exp. mass [Da]
YopO N624C /w **9•**	$C_{3219}H_{5126}N_{897}O_{994}S_{27}$	73,162	73,162
YopO Y588C/N624C /w **9•**	$C_{3257}H_{5164}N_{898}O_{999}S_{40}$	74,167	74,167
YopO Y588C/N624C /w **8•**	$C_{3261}H_{5168}N_{898}O_{1003}S_{40}$	74,283	74,284

4. Redox Stability

4.1 Cyclic Voltammetry

Cyclic voltammetry was carried out in a typical three electrode cell equipped with a Luggin capillary and a capacity of 30 mL. The working electrode was a glassy-carbon disc electrode with a surface area of 24.7 mm^2, and a gold sheet with an area of 50 mm^2 was used as the counter electrode. As the reference electrode, a reversible hydrogen electrode (RHE) was constructed from a platinum wire in PBS buffer by forming an H_2-bubble at the wire by electrolysis. For the measurement itself, a Bipotentiostat (Model AFCBP1, PINE research, Durham, NC, USA) and an AD converter connected to LabVIEW (National Instruments, Austin, TX, USA) were used. The cyclic voltammogram was also recorded via LabVIEW.

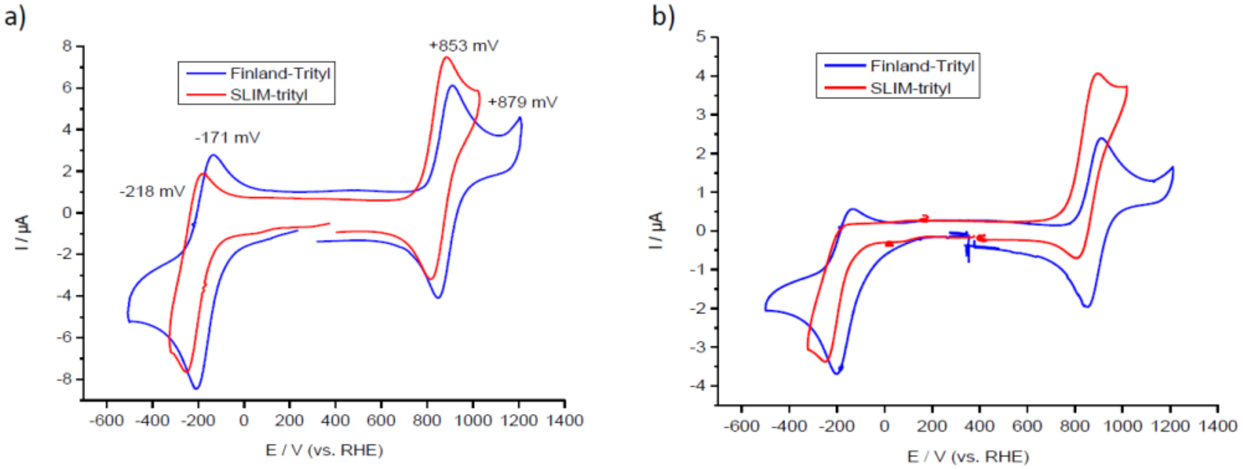

***Figure S28**: Cyclic voltammogram of SLIM **9•** (red) and Finland-Trityl **1•** (blue), each 500 µM in PBS buffer at pH 7.4 with a RHE at pH 7.4 as reference. A scan rate of a) 50 mV/s and b) 10 mV/s was applied in clockwise direction. Compared to the Finland-Trityl (blue), the reoxidation of the trityl-anion becomes less reversible, especially at 10 mV/s, for the SLIM-case (red), which means, that the quenching process with H^+ is faster in that case. The reason for this lies in the increased reactivity of the SLIM-anion, since it is thermodynamically less favored.*

4.2 Reduction Stability

4.2.1 Cell Lysates Preparation

Oocytes of the African claw frog *Xenopus laevis* were obtained from EcoCyte Bioscience (Dortmund, Germany) and lysate was prepared following a protocol of *Karthikeyan et al.*[13]. Within this procedure, the oocytes were mechanically disrupted and solid ingredients as well as the lipid layer was separated off by centrifugation. In total, 70 µL of lysate were obtained from 100 oocytes.

For HeLa cell lysate, HeLa S3 (ATCC® CCL-2.2, human cervical adenocarcinoma) were suspended in PBS buffer (600 µL per 10^8 cells) and the suspension was frozen in ethanol/$CO_{2(s)}$ for 5 min and subsequently thawed in and 37 °C water bath. This procedure was done three times, whereafter the cells were lysed with rapid oscillation within 5 minutes. Membranes were separated by centrifugation at 15000 rpm at 4 °C and the lysate was collected as the supernatant. It was separated into several aliquots and immediately shock frozen in liquid nitrogen and stored at –80 °C.

4.2.2. Stability Measurements

As outlined in the main text, the stability of SLIM **9•** and representatives of other spin label classes were assessed. For this, the respective label was diluted to a concentration of 200 µM in the corresponding medium and filled into a 10 µL capillary, which was then sealed with superglue and transferred into a 3 mm Q-band tube. In analogy to this, the labeled YopO N624C mutant was studied at 50 µM. The tube was then inserted into the spectrometer (EMXmicro) and the spectrometer was tuned properly. After this, a *cw*-EPR spectrum was recorded every 15 minutes over 15 hours. For reproducible results, it was crucial to warm up the spectrometer in advance to the measurement. Additionally, a home-written bash script was used to monitor the microwave frequency and power throughout the entire measurement. The signal intensities representing the spin concentration are obtained as the double integral over the spectrum for each time point. The dead time, measured from addition of the radical to the respective medium to the beginning of measurement, was kept below 6 minutes.

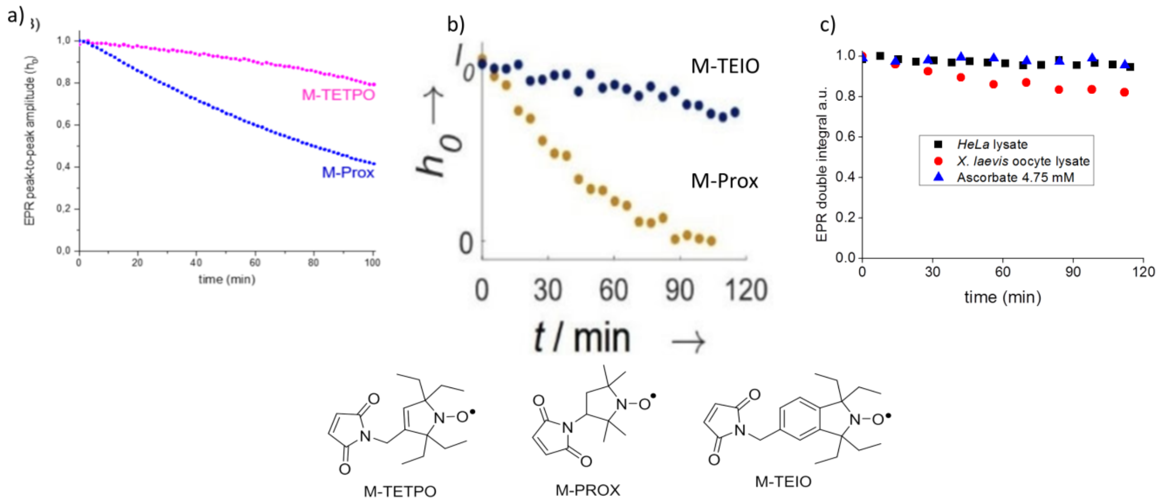

Figure S29: *Stability of various spin labels compared to* ***9•****. a) Spin labels M-TETPO and M-PROX in Xenopus laevis oocyte lysate. Excerpt from Karthikeyan et al., Angew. Chem.* ***2018****, 57, 1366 – 1370. © 2018 Wiley VCH Verlag GmbH & Co. KGaA, Weinheim, Germany. b) Spin labels M-TEIO and M-PROX in HEK (humane embryonal kidney cells) lysate. Excerpt from T. S. Braun et al., ChemBioChem* ***2019****, accepted manuscript, DOI: 10.1002/cbic.201900537. © to the authors, published by Wiley-VCH Verlag GmbH & Co. KGaA, Weinheim, Germany. c) Stability of* ***9•*** *in various media.*

5. Simulation of *cw*-EPR spectra

cw-EPR spectra were simulated using the EasySpin[14] toolbox for MATLAB (MathWorks, Natick, MA, USA). All obtained g-values, hyperfine coupling constants and peak-to-peak linewidths are summarized in Table S6. The spectra of the free (i.e. non-bioconjugated) trityl radicals in liquid solution (SLIM in PBS-buffer, Figure 2a in the main text; SLIM in DMSO, Figure S30a; **15•** in DMSO, Figure S30b) have been fitted using the "garlic" routine of EasySpin. Appropriate starting values for the "esfit" routine of EasySpin were taken from the study of *Bowman et al.*[15]

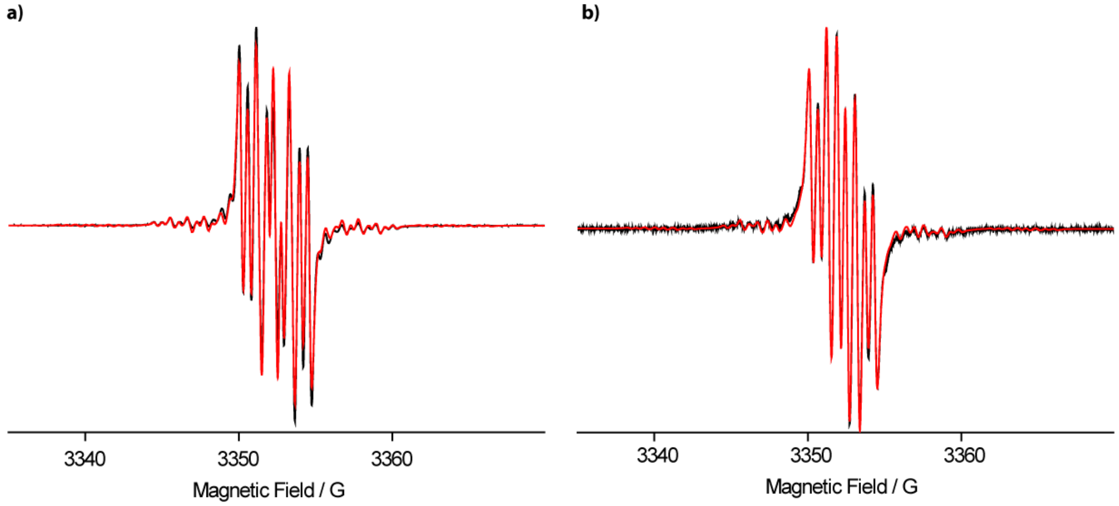

Figure S30*: cw-EPR spectra of* ***9•*** *in DMSO (a) and of* ***15•*** *in DMSO (b) recorded at 298 K. The EasySpin simulation is overlaid as a red line.*

The spectrum of free SLIM in frozen solution at 100 K (Figure 2b in the main text) has been simulated with the "pepper" routine of EasySpin. The starting values of the anisotropic g- and A-tensors have been obtained as described in the following. To start, the percentage contributions pc_{xx}, pc_{yy}, pc_{zz} of g_{xx}, g_{yy} and g_{zz} to the isotropic g-value obtained from a DFT computation (SI, section 8.1) have been determined.

$$pc_{xx} = \frac{g_{XX}^{DFT}}{g_{iso}^{DFT}}\,;\, pc_{yy} = \frac{g_{YY}^{DFT}}{g_{iso}^{DFT}}\,;\, pc_{zz} = \frac{g_{ZZ}^{DFT}}{g_{iso}^{DFT}}$$

This procedure was also applied to DFT-derived hyperfine coupling constants A. With these percentages at hand, appropriate starting values (guesses) for the EasySpin fitting could be obtained by multiplying the percentage values with the isotropic g-value and hyperfine coupling constants extracted from the solution spectrum of free SLIM.

$$g_{XX}^{guess} = pc_{XX} \cdot g_{iso}^{fit} \cdot 3$$

The factor 3 in this equation is relevant to finally fulfill the following condition.

$$\frac{g_{xx} + g_{yy} + g_{zz}}{3} = g_{iso}$$

The spectrum of SLIM upon bioconjugation to YopO shown in Figure 2c the main text has been fitted with the "pepper" routine of EasySpin. The results obtained from the simulation of free SLIM in the frozen state were taken as starting values.

Table S6: cw X-band EPR Fitting Parameters

Sample	Fitting Results	
(a) **9•**<br>298 K, DMSO	g = 2.0034<br>A_N = 1.48 MHz<br>A_{H1} = 3.48 MHz<br>A_{H2} = 6.06 MHz<br>LWPP = (0.007, 0.029) mT	$A_{C,central}$ = 66.16 MHz<br>$A_{C,ipso}$ = 31.22 MHz<br>$A_{C,ortho}$ = 25.45 MHz<br>$A_{C,para}$ = 6.86 MHz<br>$A_{C,meta}$ = 3.57 MHz
(b) **9•**<br>298 K, PBS	g = 2.0033<br>A_N = 1.71 MHz<br>A_{H1} = 2.96 MHz<br>A_{H2} = 6.00 MHz<br>LWPP = (0.006, 0.018) mT	$A_{C,central}$ = 66.91 MHz<br>$A_{C,ipso}$ = 31.63 MHz<br>$A_{C,ortho}$ = 25.65 MHz<br>$A_{C,para}$ = 7.27 MHz<br>$A_{C,meta}$ = 3.90 MHz
(c) **15•**<br>298 K, DMSO	g = 2.0034<br>A_N = 1.51 MHz<br>A_{H1} = 3.39 MHz<br>A_{H2} = 5.16 MHz<br>LWPP = (0.002, 0.034) mT	$A_{C,central}$ = 66.10 MHz<br>$A_{C,ipso}$ = 31.11 MHz<br>$A_{C,ortho}$ = 25.43 MHz<br>$A_{C,para}$ = 6.83 MHz<br>$A_{C,meta}$ = 3.51 MHz
(d) **9•**<br>100 K, PBS	g = (2.0028, 2.0036, 2.0040)<br>A_N = (2.69, 0.64, 1.44) MHz<br>A_{H1} = (4.14, 0.75, 2.86) MHz<br>A_{H2} = (7.28, 7.55, 7.49) MHz<br>LWPP = (0.089, 0.025) mT	$A_{C,central}$ = (3.45, 6.24, 185.27) MHz<br>$A_{C,ipso}$ = (37.42, 37.64, 16.24) MHz<br>$A_{C,ipso}$ = (38.11, 35.00, 21.01) MHz<br>$A_{C,ipso}$ = (34.80, 34.87, 23.92) MHz
(e) YopO N624C /w **9•**<br>298 K, PBS	g = (2.0034, 2.0034, 2.0035)<br>A_N = (2.19, 0.14,0.94) MHz<br>A_{H1} = (3.64, 1.25, 2.36) MHz<br>A_{H2} = (6.96, 5.05, 7.99) MHz<br>LWPP = (0.051, 0.079) mT	$A_{C,central}$ = (3.92, 6.53, 184.78) MHz<br>$A_{C,ipso}$ = (36.92, 37.14, 15.75) MHz<br>$A_{C,ipso}$ = (37.61, 34.50, 21.49) MHz<br>$A_{C,ipso}$ = (34.30, 34.37, 24.42) MHz

The observed ^{13}C-hyerfine coupling constants are similar to those reported for the Finland trityl[15].

6. *In Cell* Sample Preparation

Stage IV *Xenopus laevis* oocytes were purchased from EcoCyte Bioscience (Dortmund, Germany). All oocytes were visually checked for signs of apoptosis or other cell defects prior to use and only intact oocytes were used for subsequent experiments. Up to 30 oocytes were aligned on a plate with the darker animal hemisphere pointing towards the injection needle and kept moist using MBS buffer (5 mM HEPES pH 7.8, 88 mM NaCl, 1 mM KCl, 1 mM $MgSO_4$, 2.5 mM $NaHCO_3$, 0.7 mM $CaCl_2$). Injection needles were pulled over a Bunsen burner using glass capillaries with an outer diameter of 0.3 mm. Sufficiently thin needles were then filled with light silicone oil, mounted onto a Drummond Nanoject II microinjector (Broomall, PA, USA) and filled with SLIM-labeled YopO Y588C/N624C protein solution (~400 µM protein). For injection, oocytes were placed onto a homemade acryl glass plate with 1.5 mm x 1.5 mm grooves, which was covered with Parafilm (Bemis, Neenah, WI, USA) in advance. Each oocyte was carefully injected with 59.8 nL of protein solution, paying attention to not destroy the outer membrane. The injection itself was carried out into the dark animal hemisphere. If leakage occurred or the oocyte was damaged in other fashion during the injection process, it was discarded using a pipette. After injection of all oocytes, the oocytes were washed off the injection plate into a petri dish using MBS buffer where additional buffer was used to wash the oocyte's surface. Each oocyte was visually checked again for damage and then transferred into a Q-band tube filled to the top with MBS buffer. After up to 20 oocytes were transferred into the tube, excess buffer was removed using a syringe and the oocytes were incubated at 18° C for two hours to allow the protein to equilibrate and homogeneously distribute within the cell. After incubation, the sample was visually checked again and all oocytes appeared with intact spherical shape and the surrounding buffer was clear. Damaged oocytes can be recognized by loss of spherical shape and a nearby turbidity of the surrounding buffer caused by leaking cytosol. Subsequently, the Q-band tube was flash-frozen in liquid nitrogen. Key steps of the preparation and injection procedure are displayed in Figure S31.

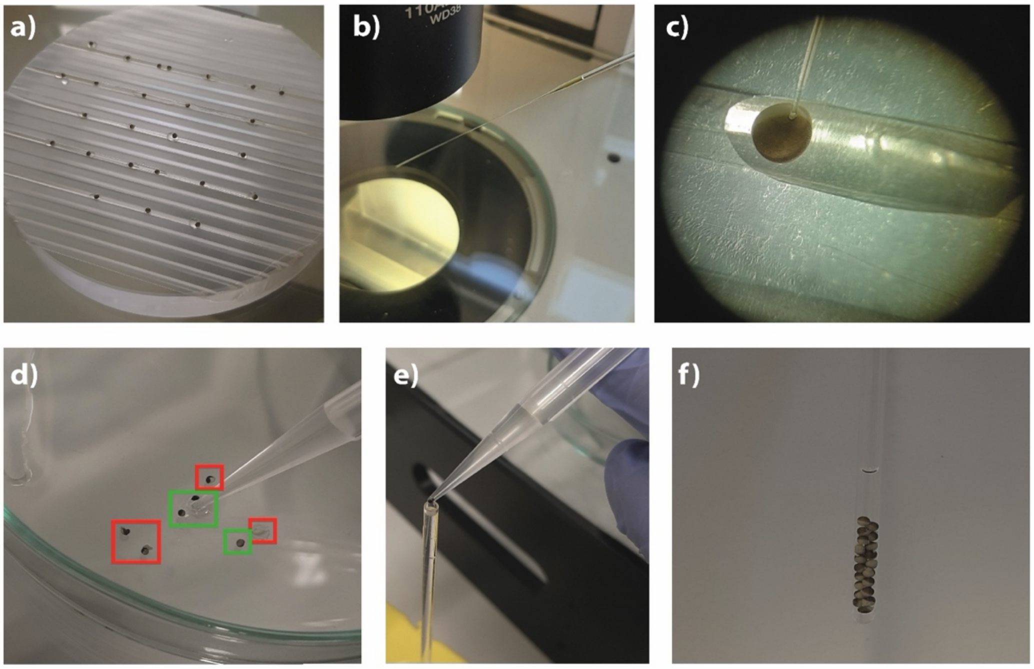

***Figure S31**: Preparation of *Xenopus laevis* oocytes for in-cell measurement. a) Alignment of the oocytes on the Parafilm-covered acryl glass plate. b) Injection needle with pulled up YopO protein. c) Penetration and injection process of an oocyte, picture taken with a smartphone through the lens of the microscope. d) Visual inspection of the oocytes after washing. Cells in the red square provide the instance of damage and were sorted-out subsequently whereas the ones in the green boxes were considered suitable for further experiments. e) Oocyte transfer into the Q-band tube with an Eppendorf pipette. f) Oocytes filled into the Q-band tube after incubation for 2h.

The maximum bulk concentration limit of spins was estimated using the following equation:

$$c_{\text{spin bulk}} = \frac{N_{\text{oocytes}} * c_{\text{Spin}} * 60\,nL}{V_{\text{Q-band tube}}} = \frac{20 * 800\ \mu M * 60\,nL}{70\mu L} = \mathbf{13.7\mu M}$$

Note that this estimation does not account for label degeneration during the two hours of incubation. The final spin concentration is estimated to be approximately 11 µM taking into account the findings of the label stabilities assessed by *cw*-EPR shown in Figure S29.

7. Pulsed EPR

7.1 EPR Sample Preparation

YopO samples were diluted 1:1 in ethylene glycol-d_6 to a final protein concentration of 25 µM, transferred into a Q-band EPR tube (O.D. 3 mm, Wilmad LabGlass, Vineland, NJ, USA) and flash-frozen in liquid nitrogen.

7.2 Relaxation Time Measurements

For the sake of comparison with other spin labels, the relaxation behavior of **9•** bound to the N624C YopO single cysteine mutant was evaluated with respect to T_1 and T_M obtained by inversion recovery (IR, Figure S32a) and two-pulse electron spin echo envelope modulation (2pESEEM, Figure S32b) experiments. The pulse sequences were applied at the maximum of the echo-detected field swept EPR spectrum and included phase cycling, two steps for 2pESEEM and four steps for IR. Acquisition parameters are given in Table S7.

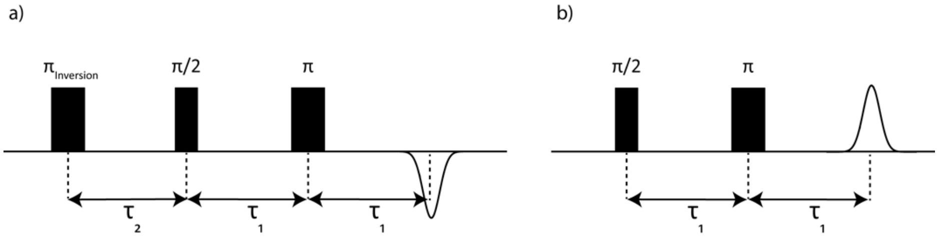

Figure S32: *Pulse sequence applied for the inversion recovery (a) and the two-pulse ESEEM (b) experiment.*

Table S7: *Pulse sequence parameters for relaxation time measurements.*

Inversion Recovery		Two-Pulse ESEEM	
**Variable**	**Value**	**Variable**	**Value**
π/2	12 ns	π/2	12 ns
π	24 ns	π	24 ns
$\pi_{\text{Inversion}}$	24 ns	–	–
τ_1	200 ns	τ_1	200 ns
τ_2	400 ns	–	–
τ_2 increment	1 ms	τ_1 increment	8 ns
Shots per Point	1	Shots per Point	1
Shot Repetition Time	1 s (10 K, 30 K)<br>500 ms (50 K)	Shot Repetition Time	500 ms

The results of the relaxation time measurements are summarized in Figure S33.

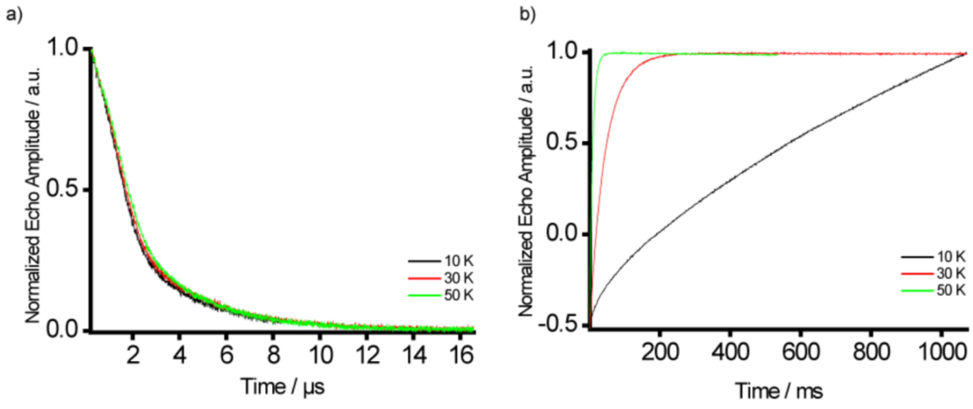

Figure S33: *Hahn echo decay curves (a) and inversion recovery traces (b) recorded on **9•** bound to the N624C YopO single cysteine mutant.*

7.3 Double Quantum Coherence (DQC) Experiments

The six-pulse Double Quantum Coherence (DQC) sequence (Figure S34) was applied at the magnetic field position corresponding to the maximum of the trityl field-swept EPR spectrum. The phase of the microwave radiation was adjusted on the Hahn echo sequence as to yield a maximally positive amplitude in the real signal channel of the quadrature detector. Proper phase adjustment was checked by summing amplitudes of the Hahn echo obtained from $(\pi/2)_{+x}/(\pi)_{+x}$ and $(\pi/2)_{-x}/(\pi)_{-x}$ pulses which averages out the signal. Pulse lengths and interpulse delays used for the DQC experiment are given in Table S8. The shot repetition time (SRT) was set to 15.3 ms.[16] A 64–step phase cycle was applied to remove undesired echoes and thus extract the pure double quantum coherence pathway contributions.[17,18] In order to eliminate deuterium ESEEM from the dipolar traces, a modulation averaging procedure was applied (τ_1 and τ_2 in 8 steps of 16 ns).[19] DQC measurements on YopO double cysteine mutants labeled with either SLIM or **8•** were performed at 50 K. In order to eliminate the background of the PDS time traces, a background correction[20] was performed. In the case of DQC (in cell and in vitro), polynomials of third order were used to fit the background contribution. Background correction was done using the DeerAnalysis package, and the "background start" value was chosen such that the corrected time trace was flat at long dipolar evolution times (last quarter of the time trace). In order to obtain an initial guess of the "background start" value, we used the "!" button in DeerAnalysis. The influence of the background on the distance distributions was checked by means of the validation tool in DeerAnalysis. In the present work, only the "background start" parameter was varied, and the variation ranges were set from the first local minimum of the time trace to the point when the oscillations were entirely damped.

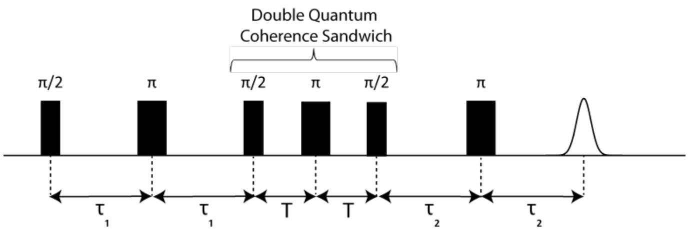

Figure S34: Pulse sequence applied for the DQC experiment.

DQC data were mirrored at the zero-time origin. Next, background correction was performed by DeerAnalysis[21] using a third order polynomial. Subsequently, the background-corrected dipolar traces were translated into distance distributions via Tikhonov regularization with the optimal regularization parameter α determined according to the L-curve corner criterion. Finally, uncertainty estimations of the distance distributions were computed using the validation routine of DeerAnalysis.[21]

Table S8: Parameters of the DQC experiment.

Variable	Value
π/2	12 ns
π	24 ns
τ_1	250 ns
τ_2	4000 ns
T	50 ns
Shots per Point	3
Shot Repetition Time	15.3 ms

7.4 Pulsed Electron-Electron Double Resonance (PELDOR) Experiments

Pulsed Electron-Electron Double Resonance (PELDOR, Figure S35) Spectroscopy was performed both on trityl- and nitroxide-labeled samples of YopO. Pulse lengths and interpulse delays are listed in Table S9. All PELDOR measurements were conducted at 50 K.

For the trityl-labeled YopO samples (**8**$^\bullet$ and **9**$^\bullet$), the pump pulse $(\pi)_B$ was set to the maximum of the field-swept EPR spectrum and the observer pulses were applied at a frequency offset of –15 MHz relative to the pump frequency. The length of the pump pulse $(\pi)_B$ was determined by a transient nutation experiment. Regarding the suppression of deuterium ESEEM, an 8-step modulation averaging procedure was applied with a time increment of 16 ns. Additionally, a two-step phase cycle was used in order to remove undesired echoes and to correct for receiver baseline offsets.

For the PELDOR experiment on **R1**-labeled YopO, the pump pulse was applied at the magnetic field position which yields the maximal signal amplitude. The detection sequence was applied at a frequency offset of -100 MHz with respect to the pump frequency. The optimal length of the $(\pi)_B$ pump pulse was determined by a transient nutation experiment. As mentioned above, a modulation averaging procedure (8 steps with a 16 ns increment) and a two-step phase cycle was used to average out deuterium ESEEM and to remove unwanted echoes as well as baseline offsets. Background correction of the PELDOR time traces was done assuming a 3-dimensional homogeneous distribution. Validation of the background was done in the same way as described above for the DQC data.[22]

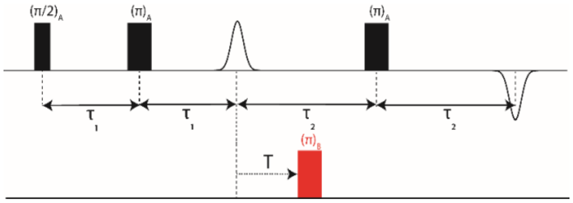

Figure S35: Pulse sequence applied for the PELDOR experiment.

Table S9: Parameters of the PELDOR experiments

Variable	**8**$^\bullet$ / **9**$^\bullet$	MTSL
$(\pi/2)_A$	32 ns	12 ns
$(\pi)_A$	64 ns	24 ns
$(\pi)_B$	60 ns	16 ns
τ_1	260 ns	260 ns
τ_2	4000 ns	4000 ns
Shots per Point	3	3
Shot Repetition Time	15.3 ms	1 ms

7.5 YopO PDS Results

Primary data of the YopO double mutant Y588C/N624C obtained by the different PDS techniques mentioned above are summarized in Figure S36.

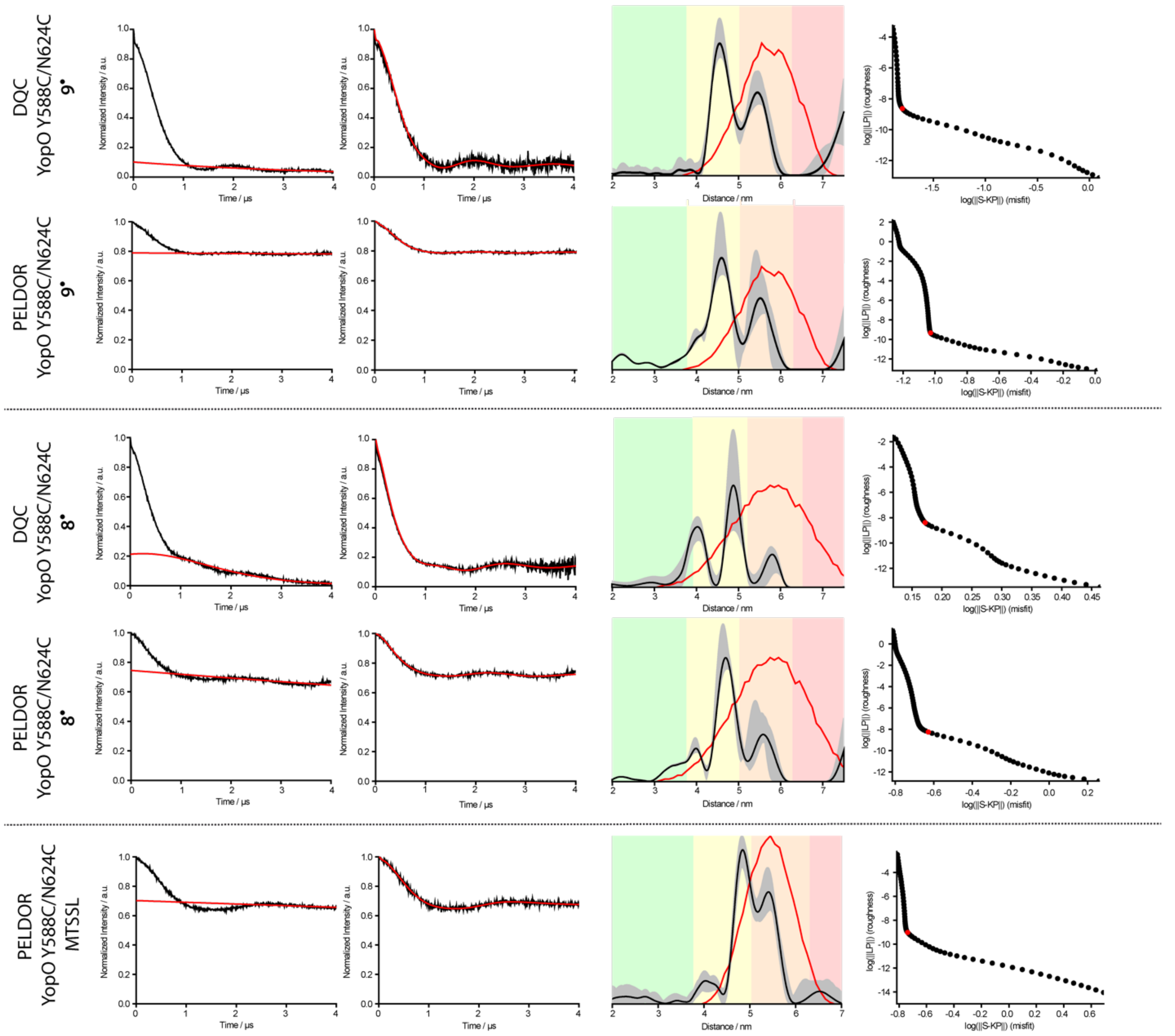

Figure S36: *PDS data obtained for YopO Y588C/N624C. The red curves in the third column show the in silico predictions of the distance distributions computed by mtsslWizard using the PDB-ID 2h7o.*

It should be noted, that the DQC modulation depth should be 100 % according to theory. However, a in experiments the modulation depth is usually less (80% - 90%) as in the presents case and also as reported for model compounds. This can be attributed to the experimental conditions, where slight imperfections in the phase cycling reduce its efficiency as a double-quantum filter.[23]

The signal-to-noise ratio (SNR) of the dipolar traces can be defined as

$$SNR = \frac{\lambda}{\sigma_N} \cdot \frac{1}{\sqrt{t}}$$

with modulation depth of the trace λ, the acquisition time of the experiment t and the standard deviation of the noise of the trace σ_N. The noise has been deconvolved from the signal by fitting a polynomial of 8th order to the raw dipolar traces. The pure noise contributions have been obtained by subtracting the fit from the raw data. The SNR has been computed from the primary data prior to background correction by means of the software SnrCalculator.[24] The SNR values obtained by this method are summarized in Table S10.

***Table S10**: Signal-to-Noise Ratios obtained for the PDS Experiments on YopO Y588C/N624C.*

**Experiment**	**YopO Y588C/N624C 9•**	**YopO Y588C/N624C 8•**	**YopO Y588C/N624C R1**
DQC	674 $h^{-1/2}$	503 $h^{-1/2}$	–
PELDOR	155 $h^{-1/2}$	131 $h^{-1/2}$	248 $h^{-1/2}$

In order to quantify the widths of the bimodal distance distributions obtained using SLIM/DQC and MTSL/PELDOR, the distance distributions were fitted as a sum of two Gaussian functions:

$$y = y_0 + \frac{A}{w\sqrt{\pi/2}} exp\left(-2\left(\frac{x-x_c}{w}\right)^2\right)$$

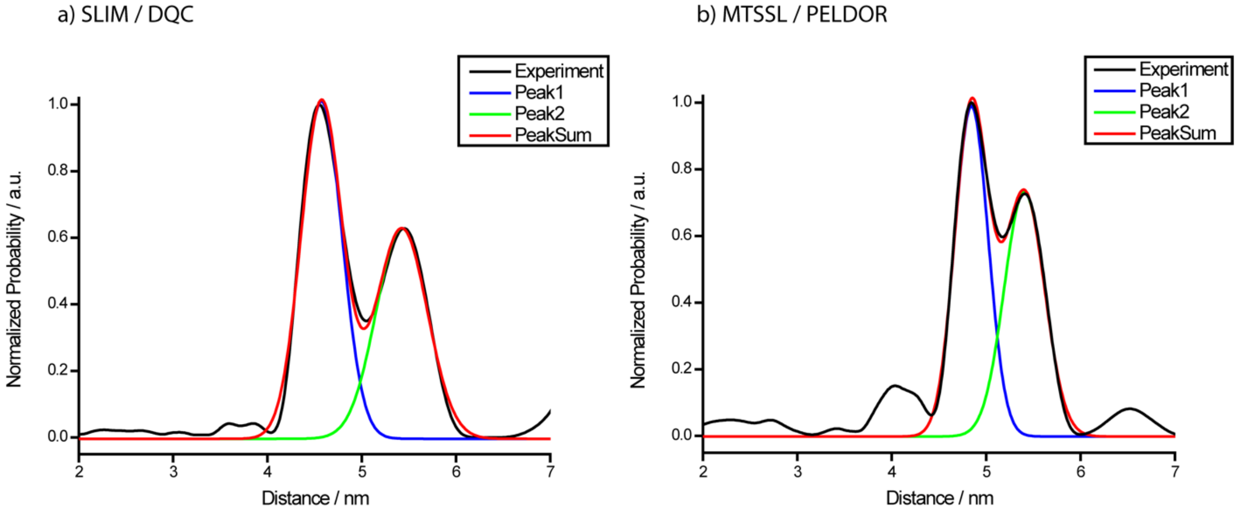

***Figure S37**: Analysis of the distance distribution bimodality with Gaussian functions.*

***Table S11**: Gaussian function parameters obtained for the fits shown in Figure S46.*

	**SLIM** / DQC	**MTSL** / PELDOR
Peak 1	Area: A = 0.55<br>Center: x_c = 4.57 nm<br>Width: w = 0.43 nm<br>Height: 1.01	Area: A = 0.44<br>Center: x_c = 4.84 nm<br>Width: w = 0.35 nm<br>Height: 0.99
Peak 2	Area: A = 0.43<br>Center: x_c = 5.42 nm<br>Width: w = 0.54 nm<br>Height: 0.63	Area: A = 0.39<br>Center: x_c = 5.41 nm<br>Width: w = 0.43 nm<br>Height: 0.73

7.6 PDS on YopO with human platelet actin

To validate the influence of actin binding to the α-helix 14 conformation, the YopO Y588C/N624C construct labeled with **9**$^{\bullet}$ was incubated with human platelet actin. For this purpose, 1 mg human platelet actin (Cytoskeleton, Inc., Denver, CO, USA) was dissolved in 100 µL D_2O. Next, 50 µM **YopO-9**$^{\bullet}$ was incubated with a 2-fold molar excess of actin and a 10-fold molar excess of the actin polymerization inhibitor latrunculin B (Merck KGaA, Darmstadt, Germany) in deuterated YopO-actin Buffer (4 mM TES pH 7.5, 0.4 mM ATP, 0.2 mM $CaCl_2$, 2 mM NaN_3) for 2 h on ice prior to the addition of 50 % deuterated ethylene glycol and flash freezing. Subsequently, the DQC experiment was performed as described in section 7.3 (Figure S38).

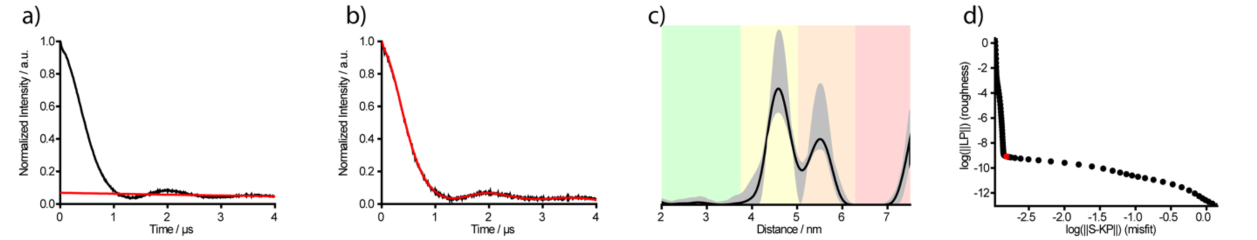

Figure S38: *a) Mirrored DQC time trace of YopO Y588C/N624C labeled with* ***9***$^{\bullet}$ *incubated with human platelet actin. The background function is indicated as a red line. b) Background-corrected time trace of (a) with the fit for Tikhonov regularization shown in red. c) Distance distribution obtained via Tikhonov regularization of (b) and the corresponding DeerAnalysis background validation shown in grey. d) L-curve with the chosen regularization parameter α shown in red.*

Interestingly, also in complex with human actin a bimodal distance distribution is obtained (Figure S38c). Overlaying both distance distributions obtained for **YopO-9**$^{\bullet}$ in the absence and presence of actin shows that the conformation of α-helix 14 is independent of the actin binding process since both distributions are almost identical (Figure S39a). These data are in contrast to the two deposited crystal structures of YopO in the absence (PDB-ID: 2h7o) and presence (PDB-ID: 4ci6) of actin that show either a straight or bent conformation (Figure S39b+c). Here, we could demonstrate that in solution the α-helix 14 of YopO always resembles both, the straight and bent conformation, statistically and independently of the presence of actin.

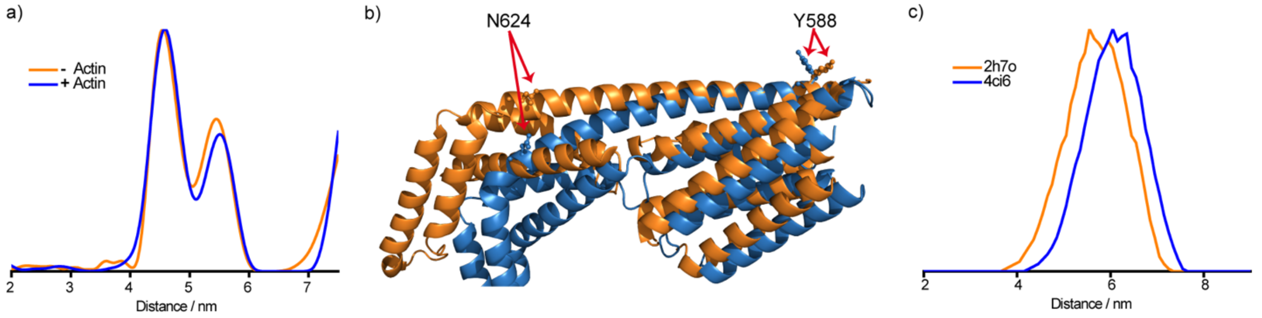

Figure S39: *a) Overlaid distance distributions of* ***YopO-9***$^{\bullet}$ *in the absence (orange) and presence (blue) of actin. b) Superposition of the GDI domains of the PDB-IDs 4ci6 (blue) and 2h7o (orange). b) Comparison of the in silico distance distributions of Y588C/N624C labeled with* ***9***$^{\bullet}$ *obtained for the different crystal structures of YopO (blue, PDB-ID: 4ci6) and YpkA (orange, PDB-ID: 2h7o).*

7.7 Evaluation of Concentration Limit for SLIM / DQC

In order to evaluate the lower concentration limit feasible for DQC experiments in combination with SLIM, YopO Y588C/N624C labeled with **9**$^{\bullet}$ was diluted in PDS buffer. Q-band PDS samples were prepared with the

corresponding final protein concentrations in 30 % glycerol-d_8. The final spin concentration was verified by comparing the normalized amplitudes of the echo-detected field swept EPR spectra (Figure S40) of the nanomolar samples to the respective values obtained from the doubly labeled 25 µM YopO Y588C/N624C-**9•** reference sample.

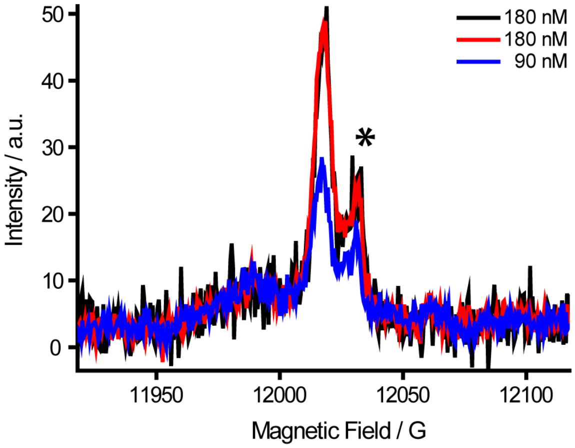

***Figure S40**: Field swept EPR spectra of nanomolar YopO Y588C/N624C-**9•** samples. The asterisk marks the E' signal stemming from the EPR tube.*

As can be seen in Figure S41, even at nanomolar concentration ranges, DQC experiments can be successfully performed, yielding time traces at a SNR of 2 $h^{-1/2}$ in both cases (note that the trace length at 90 nM was set to 2.5 µs instead of 4 µs). Both time traces recorded at nanomolar concentrations exhibit the same oscillation period as the 25 µM reference sample, thus underlining the reliability of the measurements and proving the possibility of DQC at nanomolar concentrations.

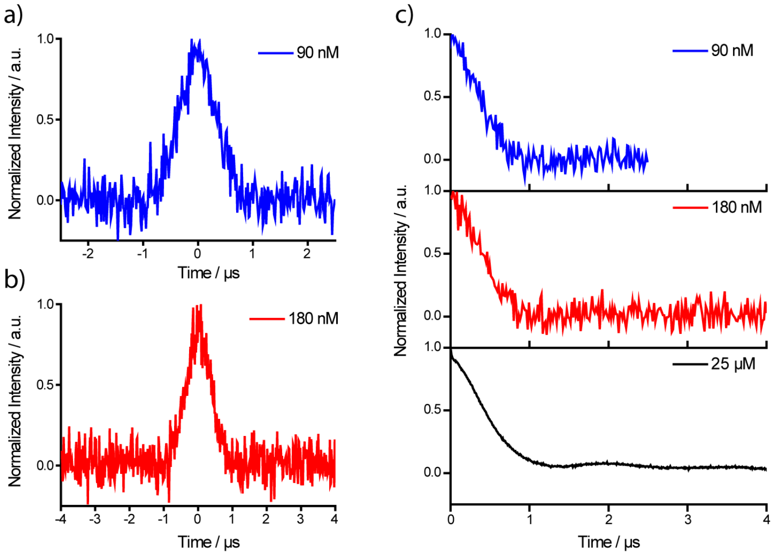

***Figure S41**: Original DQC time traces of YopO Y588C/N264C labeled with SLIM at a protein concentration of 90 nM (a) and 180 nm (b), respectively. c) Mirrored DQC time traces of the YopO samples at the given protein concentrations with the 25 µM reference sample (black, bottom) from section 7.5 as benchmark.*

7.8 *In Cell* Pulsed EPR Measurements

In order to check for the successful injection and the presence of trityl-labeled YopO within the oocytes, echo-detected field swept EPR spectra were recorded from untreated oocytes and after injection. Field swept spectra obtained from untreated oocytes show signals stemming from Mn(II) and an additional signal at g = 2.0054 which may stem from an organic radical endogenously present in oocytes (Figure S42a). After injection of SLIM-labeled protein, the trityl signal at g = 2.0037 dominates the spectrum and thus proves successful injection (Figure S42b).

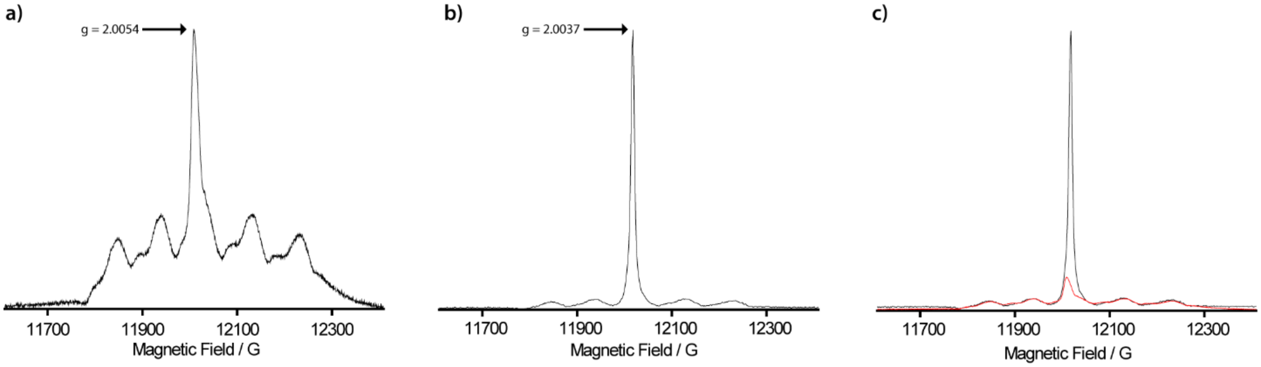

Figure S42*: a) Echo-detected field swept EPR Spectrum of pure oocytes prior to injection of spin-labeled protein. The spectrum clearly shows the presence of Mn(II) and of an organic radical giving rise to a signal at g = 2.0054. b) Echo-detected field swept EPR spectrum of oocytes after injection of trityl-labeled YopO with the spectrum being dominated by the trityl signal at g = 2.0037. c) Overlay of field swept EPR spectra before injection (red) and after injection (black) with the signal amplitude normalized to the Mn(II) signals.*

Longitudinal and transverse electron spin relaxation times (T_1, T_M) were studied for two reasons: Firstly, they provide insight into changes of the environment of the electron spin upon injection of the label into the oocyte cell, e.g. due to the presence of Mn(II) ions.[25] Secondly, especially the long T_1 relaxation time of trityl species unambiguously proves the presence of spin label within the cell. Therefore, T_1 and T_M were measured in untreated and injected oocytes at the magnetic field positions corresponding to the Mn(II) signal (11937 G), the dominant peak in the untreated oocytes (12009 G) and the trityl signal (12018 G). For details on relaxation measurement techniques, refer to SI section 7.2.

Figure S43 summarizes the results. Subfigures (S43a) and (S43b) contrast the relaxation behavior of SLIM bioconjugated to YopO *in vitro* and *in cell* after 120 minutes of incubation, showing that both longitudinal and transverse relaxation are enhanced in the cellular medium. This may be related to the endogenously present Mn(II) acting as a relaxation enhancer, but could as well be the result of crowding of spin-labeled protein in cell.[26]

Subfigures (S43c) and (S43d) show the relaxation behavior of the different species contributing to the field sweep spectrum. Comparing especially the longitudinal relaxation times at 12009 G pre- and post-injection shows clear differences related to the presence of trityl spins which are on resonance at this magnetic field. It also clearly points out that T_1 relaxation times can indicate the successful injection of labeled biomolecules into the cells. The fact that both T_1 and T_M are shorter at 12009 G (post injection) than at 12018 G shows the admixture of the fast-relaxing endogenous organic species to the trityl. This emphasizes that a careful inspection of the echo detected field swept EPR spectrum as well as the relaxation times associated with the different species is vital for choosing the correct magnetic field position for subsequent PDS experiments.

Subfigure (S43e) shows the effect of the incubation time of the oocytes after injection before flash-freezing on the transverse relaxation times. The Hahn echo decay curves clearly show that the phase memory time is extended with the incubation time, which is related to a more homogeneous distribution of the labeled biomolecule within the cell and thus reduced intermolecular dipolar coupling between electron spins.[27] However, when optimizing the incubation time, the optimal tradeoff between the beneficial homogeneous distribution of proteins in cell and the degeneration of the spin label has to be found.

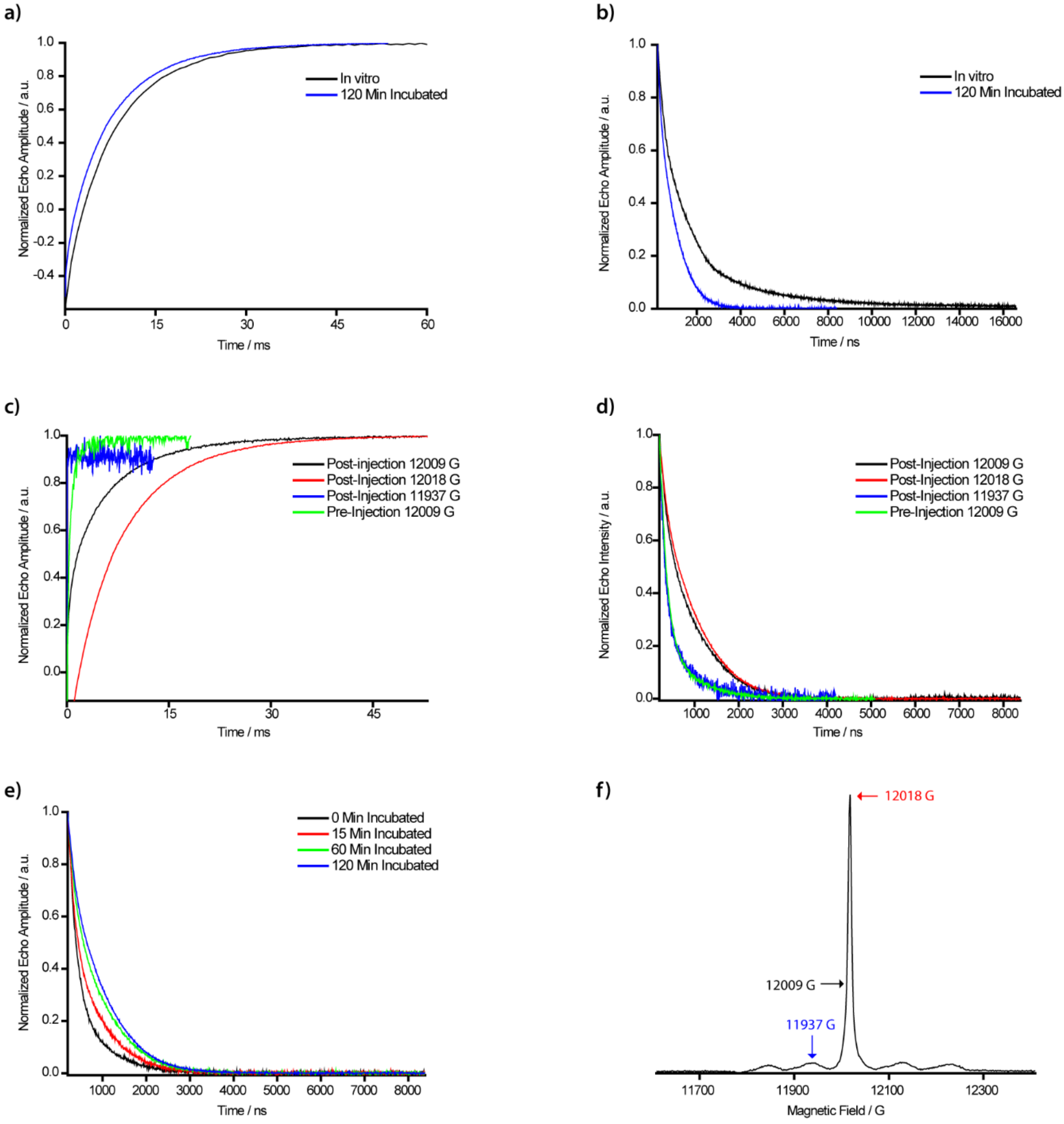

***Figure S43**: a, b) Comparison of inversion recovery traces (a) and Hahn echo decay curves (b) recorded on the trityl signal (12018 G) in vitro (black) and in oocytes after 120 minutes of incubation time. c,d) Comparison of inversion recovery traces (a) and Hahn echo decay curves at different field positions corresponding to the oocyte signal (12009 G), the trityl signal (12018 G) and the Mn(II) signal (11937 G). e) Hahn echo decay curved recorded on the trityl signal (12018 G) after different incubation times in oocytes. f) Echo-detected field swept EPR spectrum of SLIM-labeled YopO in oocytes with the relevant magnetic field positions marked by arrows.*

Due to the cellular environment and the non-deuterated buffer conditions, the acquisition parameter of the DQC experiment were adapted as summarized in Table S12 in order to optimize data acquisition. The thusly obtained *in cell* data are shown in Figure S44.

Table S12: *Parameters of the in cell DQC experiment.*

Variable	Value
$\pi/2$	12 ns
π	24 ns
τ_1	200 ns
τ_2	3500 ns
T	50 ns
Shots per Point	20
Shot Repetition Time	15.3 ms

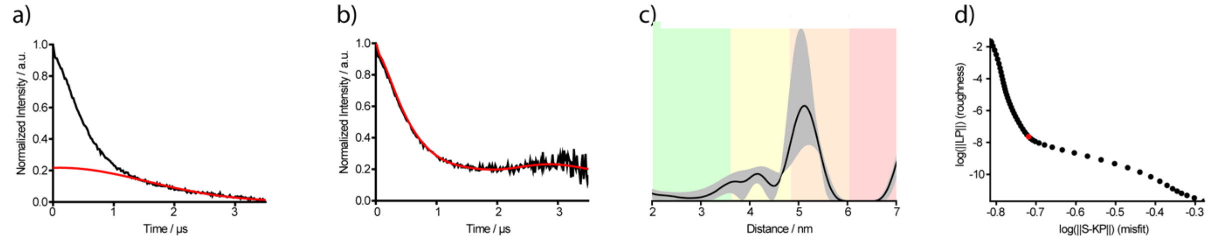

Figure S44: *a) Mirrored DQC time trace of YopO Y588C/N624C labeled with* ***9•*** *and injected into oocytes with the background function indicated as a red line. b) Background-corrected time trace of (a) with the fit for Tikhonov regularization shown in red. c) Distance distribution obtained via Tikhonov regularization of (b) and the corresponding DeerAnalysis background validation shown in grey. d) L-curve with the chosen regularization parameter α shown in red.*

The *in cell* measurement (Figure S44) shows a monomodal distribution peaking at the position corresponding to the longer distance observed in the *in vitro* measurement (5.1 nm) (Figure S36). The marginal distance shift in the *in cell* sample by 0.2 nm as compared to the *in vitro* sample may be related to uncertainties in data analysis.

8. Theoretical Results

8.1 Density Functional Theory Computations

Density Functional Theory computations on the molecules were performed on a 64-core computer cluster using the ORCA quantum chemistry software package.[28] The structure of the SLIM label has been optimized on the B3LYP/def2-TZVP[29] level of theory using the RIJCOSX approximation (def2/J as auxiliary basis[30]) and the D3 dispersion correction.[31,32] Subsequent computations of the hyperfine coupling constants of the SLIM label **9•** were done using the eprnmr module of ORCA on the B3LYP/def2-TZVP level of theory. Again, the D3 correction was applied to account for dispersion interactions. The molecular coordinates of SLIM after geometry optimization are given in Table S13.

Table S13: Atomic coordinates obtained by the geometry optimization of the SLIM label **9•** (B3LYP/def2-TZVP D3).

Atom #	x	y	z
0C	0.822437	-4.468512	0.846234
1C	0.836906	-3.378944	-0.033744
2C	-0.277385	-2.513170	-0.122785
3C	-1.465085	-2.914959	0.518484
4C	-1.507815	-4.027049	1.359900
5C	-0.345252	-4.788269	1.572933
6S	2.365651	-5.290236	1.077728
7S	2.363681	-3.021817	-0.827466
8S	-3.066299	-4.385054	2.087791
9S	-2.999576	-2.146905	0.165851
10C	3.199071	-4.608758	-0.407731
11C	-3.839912	-2.756752	1.672734
12C	-3.637991	-1.758404	2.807972
13C	-5.315318	-2.985467	1.364753
14C	3.080261	-5.596215	-1.564262
15C	4.658581	-4.321717	-0.064033
16C	1.052094	-1.455055	-4.241579
17C	0.302897	-1.640030	-3.076607
18C	0.536094	-0.875037	-1.915668
19C	1.513713	0.146944	-1.990743
20C	2.320287	0.287510	-3.130385
21C	2.103083	-0.520609	-4.262503
22S	0.563319	-2.412574	-5.634332
23S	-1.039701	-2.764592	-3.193954
24S	3.615455	1.481568	-3.001868
25S	1.925037	1.188523	-0.634814
26C	-0.356778	-3.662932	-4.644912
27C	2.837734	2.418794	-1.633010
28C	1.878102	3.465694	-2.188782
29C	3.910655	3.054649	-0.757862
30C	0.597941	-4.754896	-4.172463
31C	-1.498490	-4.226462	-5.481646
32C	-0.456815	1.303197	2.177858
33C	-0.179045	0.121516	1.477338
34C	-0.656535	-0.055615	0.165471
35C	-1.534653	0.912754	-0.342675
36C	-1.903673	2.029597	0.410410
37C	-1.333478	2.263844	1.665453
38S	0.509321	1.577533	3.628031
39S	0.987282	-0.964955	2.223533
40S	-3.174283	3.036474	-0.289618
41S	-2.355277	0.696468	-1.876441
42C	0.950376	-0.184780	3.892194
43C	-2.954208	2.432386	-2.017966
44C	-1.928818	3.294730	-2.746032
45C	-4.304013	2.431863	-2.726321
46C	-0.090524	-0.870184	4.771318
47C	2.345594	-0.269754	4.504228
48C	-1.651454	3.513353	2.451517
49C	-0.182285	-1.167104	-0.679941
50N	-1.331366	4.738927	1.735103
51C	-0.108966	5.031568	1.139118
52C	-0.311418	6.319197	0.395878
53C	-1.567304	6.720862	0.567024
54C	-2.272457	5.728718	1.439150
55O	0.898963	4.371152	1.216331
56O	-3.410552	5.759731	1.832853
57C	-0.433515	-5.909518	2.523218
58O	-1.464673	-6.459360	2.838037
59O	0.753301	-6.275861	3.058266
60C	2.906121	-0.402151	-5.496115
61O	2.507925	-0.664279	-6.607399
62O	4.175983	0.011064	-5.277491
63H	-0.992139	3.324557	-2.195284
64H	-2.304475	4.314706	-2.852359
65H	-1.733415	2.885239	-3.739103
66H	-5.028164	1.825935	-2.185254
67H	-4.196559	2.032594	-3.736678
68H	-4.685154	3.451496	-2.807886
69H	-1.086391	-0.763576	4.346240
70H	-0.092883	-0.423299	5.767702
71H	0.135831	-1.935087	4.860390
72H	3.082504	0.210701	3.864096
73H	2.626662	-1.315373	4.645177
74H	2.357191	0.213327	5.483184
75H	-1.125465	3.500500	3.403873
76H	-2.719920	3.576766	2.668455
77H	0.581135	-7.044949	3.623056
78H	4.593132	0.104008	-6.148042
79H	-2.577131	-1.595014	2.990014
80H	-4.097248	-2.131301	3.726300
81H	-4.090710	-0.799500	2.545940
82H	-5.828978	-3.367062	2.249842
83H	-5.438609	-3.702948	0.555488
84H	-5.790928	-2.045798	1.076118
85H	2.037172	-5.817407	-1.780383
86H	3.584867	-6.530087	-1.305844
87H	3.543547	-5.185410	-2.464058
88H	4.734037	-3.606491	0.752264
89H	5.176816	-3.914816	-0.935154
90H	5.163910	-5.244581	0.229331

91H	1.123182	2.997107	-2.818803
92H	2.427607	4.191896	-2.792650
93H	1.384033	3.982716	-1.365307
94H	4.579778	2.300275	-0.346790
95H	3.436322	3.596418	0.062307
96H	4.495029	3.767811	-1.343944
97H	1.028552	-5.275478	-5.030951

98H	0.066827	-5.475829	-3.546476
99H	1.405871	-4.321608	-3.587487
100H	-2.171059	-3.438137	-5.815087
101H	-2.066678	-4.953913	-4.898135
102H	-1.097025	-4.741364	-6.356653
103H	0.486133	6.784077	-0.161815
104H	-2.065948	7.598343	0.186946

For implementing the new SLIM spin label into mtsslWizard, a geometry optimization (PBE/def2-SVP) on the SLIM label attached to a cysteine residue was performed in advance.

The hyperfine coupling constants obtained by DFT on the B3LYP/def-TZVP D3 level of theory are summarized in Table S14. Only hyperfine coupling constants to those nuclei which have also been observed experimentally are listed. For comparison, the isotropic coupling constants obtained by the EasySpin[14] fit of the *cw* EPR spectrum recorded in DMSO at 298 K are indicated. The numbering of the atoms within the structure is shown in Figure S45.

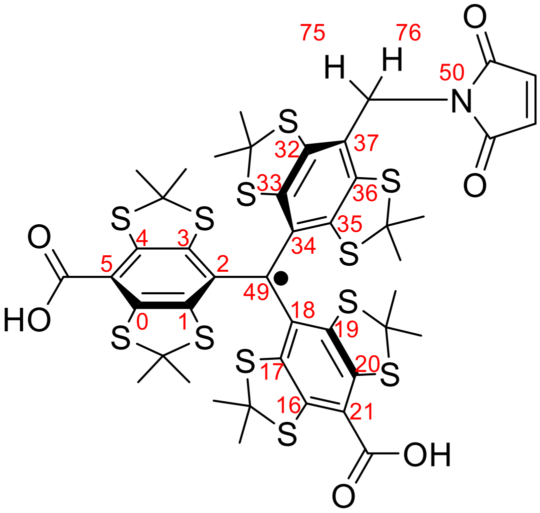

Figure S45: *Structure of* ***9•*** *with atom enumeration applied for calculation of HFC-tensors.*

***Table S14**: Hyperfine coupling constants obtained by DFT (B3LYP/def-TZVP D3) computations and fitting of the cw-EPR spectrum recorded in DMSO at 298 K.*

	Atom #	DFT B3LYP/def-TZVP D3				Experimental A_{iso} / MHz
		A_{XX} / MHz	A_{YY} / MHz	A_{ZZ} / MHz	A_{iso} / MHz	
	50N	0.8868	0.9107	1.2518	1.0164	1.48
	75H	-0.1694	-0.2959	1.2142	0.2496	3.48
	76H	4.9565	5.1968	6.4721	5.5418	6.06
	49C	4.5936	4.7467	165.4203	58.2535	66.16
ipso	2C	-24.9686	-26.7889	-42.7462	-31.5012	31.22
	18C	-24.5157	-26.3190	-42.0361	-30.9569	31.22
	34C	-24.0005	-25.0611	-34.8107	-27.9575	31.22
ortho	1C	22.6342	24.0242	43.8507	30.1697	25.45
	3C	11.4835	13.2499	33.6639	19.4658	25.45
	17C	14.2218	15.7777	35.8105	21.9367	25.45
	19C	19.5899	20.9609	43.1771	27.9093	25.45
	33C	20.0554	21.6460	34.2153	25.3056	25.45
	35C	15.3674	17.2719	28.6021	20.4138	25.45
para	5C	0.1572	1.0402	20.5164	7.2379	6.86
	21C	0.1456	1.0618	22.5647	7.9240	6.86
	37C	0.2124	0.9007	14.8309	5.3147	6.86
meta	0C	-1.9590	-2.9847	-12.0474	-5.6637	3.57
	4C	-4.8282	-5.7410	-13.6552	-8.0748	3.57
	16C	-4.1915	-5.1499	-14.3902	-7.9105	3.57
	20C	-2.8695	-3.9180	-12.8359	-6.5411	3.57
	32C	0.1930	-0.8838	-6.3693	-2.3534	3.57
	36C	-2.0127	-2.9963	-8.3120	-4.4403	3.57

8.2 *In Silico* Spin Labeling

Distance distributions were computed based on the two crystal structures of YopO deposited in the Protein Data Bank (PDB-IDs: YpkA 2h7o and YopO 4ci6) using the mtsslWizard[31] plugin for PyMOL. The maximal number of conformers to be found was 200 and the clashscore criteria were set to “loose”, implying that a *van-der-Waals* cutoff of 2.5 Å was set and 5 clashes were allowed.[32,33]

8.2.1 Conformer Cloud Volume

Conformer ensembles of **8•** and SLIM **9•** were generated on YopO N624C. The size of the conformer clouds was determined as described in the following paragraph and schematically shown in Figure S46. Basis of all computations was the prediction of the conformer cloud provided by mtsslWizard (Figure S46a+b). In order to obtain the ensemble of points in space describing the outer hull of the conformer cloud, the Cartesian coordinates of the atom which is the furthest away from the C_β-atom of the cysteine residue were extracted. Herein, the C_β-atom of cysteine serves as a fixed anchor point (Figure S46c).
Next, the overall point cloud containing the atomic coordinates of the carboxylic carbon atom and those of the hull atoms was collected (Figure S46d) which served as a basis for the computation of a so-called α-shape (Figure S46e). This α-shape obtained by the MATLAB command “alphaShape” encompasses the whole point cloud. The smallest possible alpha radius was determined automatically resulting in a tight fit of the 3D sphere to the data points. Finally, the volume of the α-shape was computed using the “volume” command of MATLAB providing a good approximation to the volume of the label conformer cloud.

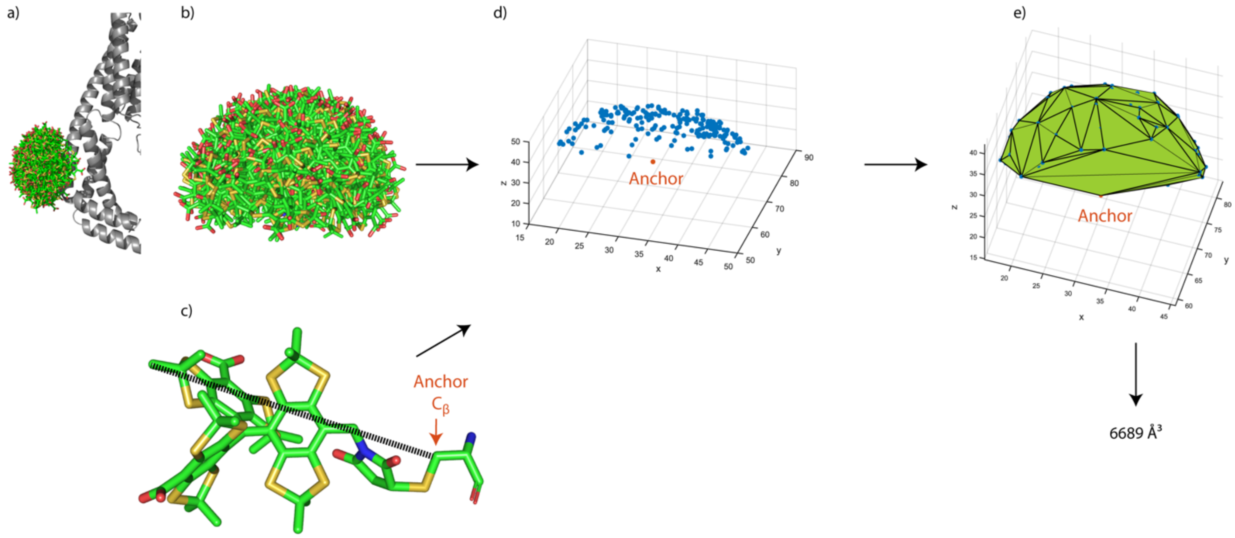

***Figure S46**: Computation of the volume of an in silico generated conformer cloud. a) YopO in silico labeled with SLIM at amino acid position N624. b) In silico generated conformer ensemble for SLIM. c) Structure of **9•** with the carboxylic carbon atom of cysteine shown as anchor point and the distance to the furthest atom indicated by a dashed line. d) Point cloud symbolizing the positions of the furthest atoms in blue and the anchor point in orange. e) Alpha-shape generated from the ensemble of coordinates shown in (d) with the resulting volume obtained by integration of the alpha-shape. The whole analysis was performed on 10 individually and independently generated conformer clouds of each of the labels SLIM and **8•**. Results are shown in Table S15.*

Table S15: *Computed volumes of the conformer clouds obtained by in silico spin labeling of YopO with* ***9***$^{\bullet}$ *and* ***8***$^{\bullet}$*.*

YopO N624C-9$^{\bullet}$		YopO N624C-8$^{\bullet}$	
Cloud #	Volume / Å^3	Cloud #	Volume / Å^3
1	6589	1	16232
2	6951	2	16637
3	6649	3	15081
4	7512	4	15316
5	7116	5	15986
6	7339	6	12855
7	6684	7	14415
8	7444	8	15539
9	6476	9	14272
10	6642	10	15669
Ø	6940	Ø	15200

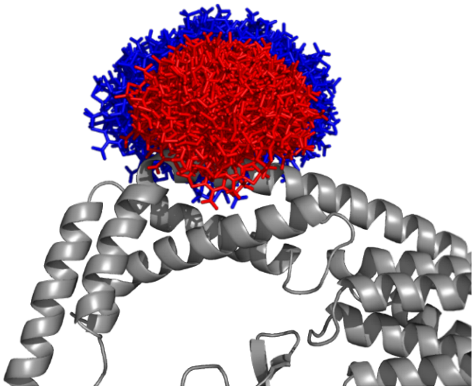

8.2.2 YopO **8**$^{\bullet}$ Label Conformer Selection

As outlined in the main text, attractive interactions between the protein's α–helix and the hydrophobic surface of the trityl radical are conspicuous to cause the short-distance artefacts for **YopO-8**$^{\bullet}$. Label conformers representative for this are provided in Figure S47 below. It becomes evident that these conformers can only emerge due to the long linker of **8**$^{\bullet}$, which bends over into the propagation direction of the helix.

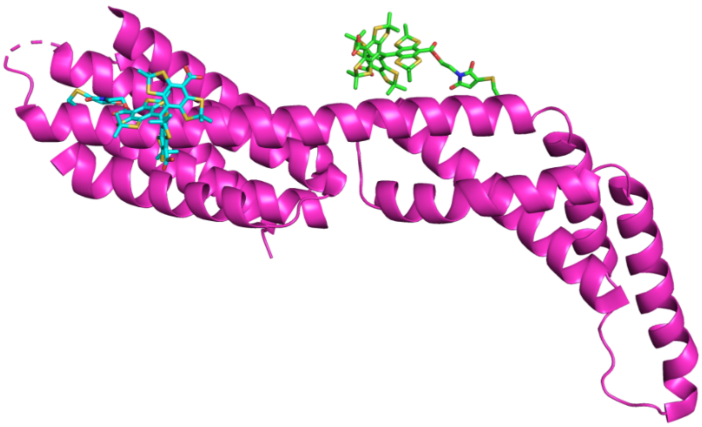

Figure S47: *Selected label conformers for* ***YopO-8***$^{\bullet}$*.*

9. Literature

[1] M. Winkle, J. Lansinger, R. C. Ronald, *J. Chem. Soc. Chem. Commun.* **1980**, 87–88.

[2] J. J. Jassoy, A. Berndhäuser, F. Duthie, S. P. Kühn, G. Hagelueken, O. Schiemann, *Angew. Chem. Int. Ed.* **2017**, *56*, 177–181.

[3] E. H. Discekici, A. H. St. Amant, S. N. Nguyen, I. H. Lee, C. J. Hawker, J. Read De Alaniz, *J. Am. Chem. Soc.* **2018**, *140*, 5009–5013.

[4] H. Hintz, A. Vanas, D. Klose, G. Jeschke, A. Godt, *J. Org. Chem.* **2019**, *84*, 3304–3320.

[5] G. R. Fulmer, A. J. M. Miller, N. H. Sherden, H. E. Gottlieb, A. Nudelman, B. M. Stoltz, J. E. Bercaw, K. I. Goldberg, *Organometallics* **2010**, *29*, 2176–2179.

[6] V. Froidevaux, M. Borne, E. Laborbe, R. Auvergne, A. Gandini, B. Boutevin, *RSC Adv.* **2015**, *5*, 37742–37754.

[7] M. M. Haugland, A. H. El-Sagheer, R. J. Porter, J. Peña, T. Brown, E. A. Anderson, J. E. Lovett, *J. Am. Chem. Soc.* **2016**, *138*, 9069–9072.

[8] C. Wuebben, S. Blume, D. Abdullin, D. Brajtenbach, F. Haege, S. Kath-Schorr, O. Schiemann, *Molecules* **2019**, *24*, 4482.

[9] J. J. Jassoy, C. A. Heubach, T. Hett, F. Bernhard, F. R. Haege, G. Hagelueken, O. Schiemann, *Molecules* **2019**, *24*, 2735.

[10] https://web.expasy.org/protparam/

[11] H. Liu, J. H. Naismith, *BMC Biotechnol.* **2008**, *8*, 91.

[12] M. Zehl, G. Allmaier, *Rapid Commun. Mass Spectrom.* **2004**, *18*, 1932–1938.

[13] G. Karthikeyan, A. Bonucci, G. Casano, G. Gerbaud, S. Abel, V. Thomé, L. Kodjabachian, A. Magalon, B. Guigliarelli, V. Belle, et al., *Angew. Chemie Int. Ed.* **2018**, *57*, 1366–1370.

[14] S. Stoll, A. Schweiger, *J. Magn. Reson.* **2006**, *178*, 42–55.

[15] M. K. Bowman, C. Mailer, H. J. Halpern, *J. Magn. Reson.* **2005**, *172*, 254–267.

[16] U. Stoll, S. (2018) (Goldfarb, D., Stoll, S., Eds.), Chapter 11, Wiley VCH

[17] P. P. Borbat, J. H. Freed, *Chem. Phys. Lett.* **1999**, *313*, 145–154.

[18] S. Saxena, J. H. Freed, *J. Chem. Phys.* **1997**, *107*, 1317–1340.

[19] D. Akhmetzyanov, P. Schöps, A. Marko, N. C. Kunjir, S. T. Sigurdsson, T. F. Prisner, *Phys. Chem. Chem. Phys.* **2015**, *17*, 24446–24451.

[20] P. P. Borbat, J. H. Freed in: *Methods in Enzymology*, Volume 423: Two-Component Systems, Part B. Eds: M. I. Simon, B. Crane, A. Crane, **2007**, Elsevier.

[21] G. Jeschke, V. Chechik, P. Ionita, A. Godt, H. Zimmermann, J. Banham, C. Timmel, D. Hilger, H. Jung, *Appl. Magn. Reson.* **2006**, *30*, 473–498.

[23] A.Meyer, J.J. Jassoy, S. Spicher, A. Berndhäuser, O. Schiemann, Phys. Chem. Chem. Phys., **2018**, *20*, 13858-13869

[23] https://github.com/dinarabdullin/SnrCalculator

[24] G.Jeschke, Y. Polyhach, *Phys. Chem. Chem. Phys*., **2007**, *9*, 1895–1910.

[25] F. Wojciechowski, A. Groß, I. T. Holder, L. Knörr, M. Drescher, J. S. Hartig, *Chem. Commun.* **2015**, *51*, 13850-13853.

[26] I. Krstic, R. Hänsel, O. Romainczyk, J. W. Engels, V. Dötsch, T. F. Prisner, *Angew. Chem. Int. Ed.* **2011**, *50*, 5070-5074.

[27] F. Neese, *WIREs Comput. Mol. Sci.* **2012**, *2*, 73–78.

[28] F. Weigend, R. Ahlrichs, *Phys. Chem. Chem. Phys.* **2005**, *7*, 3297–3305.

[29] F. Weigend, *Phys. Chem. Chem. Phys.* **2006**, *8*, 1057–1065.

[30] S. Grimme, S. Ehrlich, L. Goerigk, *J. Comput. Chem.* **2011**, *32*, 1456–1465.

[31] S. Grimme, J. Antony, S. Ehrlich, H. Krieg, *J. Chem. Phys.* **2010**, *132*, 154104.

[32] G. Hagelueken, R. Ward, J. H. Naismith, O. Schiemann, *Appl. Magn. Reson.* **2012**, *42*, 377–391.
